# Characteristic of Clinical Studies on Baduanjin during 2000–2019: A Comprehensive Review

**DOI:** 10.1155/2020/4783915

**Published:** 2020-10-16

**Authors:** Jing Zhou, Yunyang Yu, Biwei Cao, Xiaoya Li, Miao Wu, Tao Wen, Yuan Xiong, Jian Jia, Yan Zhao

**Affiliations:** ^1^First Clinical Medical College, Hubei University of Chinese Medicine, Wuhan 430061, China; ^2^Department of Tuina and Rehabilitation Medicine, Hubei Provincial Hospital of Traditional Chinese Medicine, Wuhan 430061, China; ^3^Department of Tuina and Rehabilitation Medicine, Hubei Province Academy of Traditional Chinese Medicine, Wuhan 430074, China; ^4^Department of Rehabilitation Medicine, Yichang Yiling Hospital, Yichang 443100, China; ^5^College of Acupuncture and Orthopedics, Hubei University of Chinese Medicine, Wuhan 430065, China

## Abstract

To date, a growing number of clinical studies have demonstrated the safety and health benefits from Baduanjin intervention. Based on this, our objective is to systematically retrieve and summarize the clinical studies on Baduanjin, with a view to providing more evidence-based evidence in support of the application of Baduanjin for healthcare, and to identify the shortcomings of existing research and provide feasibility suggest for further clinical research. Both four English language and four Chinese language electronic databases were used to search articles related to Baduanjin during 2000–2019. SPSS 22.0 software was used to analyze the data, and the risk of bias tool in the RevMan 5.3.5 software was used to evaluate the methodological quality of randomized controlled trials. A total of 810 publications were identified, including 43 (5.3%) systematic reviews, 614 (75.8%) randomized controlled trials, 66 (8.1%) nonrandomized controlled clinical studies, 84 (10.4%) case series, and 3 (0.4%) case reports. The top 10 diseases/conditions included diabetes, chronic obstructive pulmonary disease, hypertension, low back pain, neck pain, stroke, coronary heart disease, cognitive impairment, insomnia, and osteoporosis or osteopenia. The style of State General Administration of Sport of China in 2003 was the most commonly used version of Baduanjin, and Baduanjin was practiced with an average of 35 minutes, 1 or 2 times a day, 3–5 days per week, and a 18-week average duration. It is also worth noting that there were no serious adverse events related to Baduanjin intervention. Most studies were small sample size research, and the methodological quality of randomized controlled trials is generally low. The clinical studies of Baduanjin have a substantial quantity and evidence base. However, there are significant differences among different studies in the specific intervention measures such as style, intensity, duration, learning, and practice methods, which need to be further standardized and unified. Further high-quality designed and reporting studies are recommended to further validate the clinical benefits of Baduanjin.

## 1. Introduction

Baduanjin, also known as eight-section brocades, dates back to the Chinese Song Dynasty (10th–13th century A. D) and is a traditional Chinese sports method that uses the combination of human physical activity, breathing, and psychological regulation as elements [1]. As one is practicing the exercise, both the breathing and the body movement are slow, and the breathing is rhythmic and in harmony with the body movements. For hundreds of years, millions of Chinese have practiced Baduanjin to cultivate and maintain health. In recent years, due to its effectiveness for keeping fit, ease in learning, and economy of exercising time, Baduanjin has become popular worldwide as a promising low-intensity, physical and mental exercise.

To date, a growing number of clinical studies have demonstrated the safety and health benefits from Baduanjin intervention [2, 3]. Several systematic reviews have examined the evidence provided by Baduanjin in randomized controlled trials or nonrandomized clinical trials for various specific diseases and health conditions, such as diabetes mellitus [4–6], chronic obstructive pulmonary disease (COPD) [7], and cardiovascular disease [8, 9]. It is particularly worth noting the coronavirus disease 2019 (COVID-19) that broke out in Wuhan, China; some of the discharged patients in 2019 novel coronavirus (2019-nCoV) still have clinical manifestations such as fatigue, anorexia, and emotional abnormalities, with varying degrees of impaired lung function, changes in interstitial pneumonia, and the possibility of pulmonary fibrosis. National Health Commission and National Administration of Traditional Chinese Medicine of the People's Republic of China jointly issued the “Guidelines for Rehabilitation of Traditional Chinese Medicine in the Recovery Period of New Coronary Virus Pneumonia (Trial)” (referred to as “Rehabilitation Recommendations for Recovery Period”), suggesting that 2019-nCoV patients with light or ordinary type can adopt traditional exercises such as Baduanjin, Tai Chi Chuan, and Liuzijue after leaving hospital [10]. However, there is currently no direct evidence that Baduanjin, Tai Chi Chuan, and Liuzijue can promote the recovery of 2019-nCoV patients. In addition, with the rapid development of the peer-reviewed literature of Baduanjin, there is no comprehensive systematic review on its application in the fields of disease prevention, treatment, and rehabilitation.

Therefore, we systematically retrieved and summarized the clinical studies on Baduanjin, with a view to providing more evidence-based evidence in support of the application of Baduanjin for healthcare and to identify the shortcomings of existing research and provide feasibility suggest for further clinical research.

## 2. Methods

### 2.1. Data Sources and Searches

A comprehensive literature search was conducted by two review authors (JZ and YYY) through 3 January to 12 January 2020. Baduanjin-related articles published between 2000 and 2019 were retrieved from four well-respected English language electronic databases (PubMed, Cochrane Library, Web of Science, and Embase). The following keywords were used by the review authors: “Baduanjin,” “Baduanjin exercise,” and “eight-section Brocade.” No language restriction was applied. Taking a specific strategy as an example, the search terms in the PubMed database were as follows: (((Baduanjin[title/abstract]) or (Baduanjin exercise[title/abstract])) or (eight section brocades[title/abstract])) and ((“2000/01/01”[date-publication]: “2019/12/31”[date-publication])). In addition, the four highly respected Chinese academic databases, which included China National Knowledge Infrastructure (CNKI), SinoMed, Wanfang database, and Chinese Scientific Journal database (VIP), were also adopted to search Chinese literature by using the keyword “八段锦” (Baduanjin). Publication language is limited to Chinese or English.

### 2.2. Inclusion/Exclusion Criteria

We included all types of clinical studies with Baduanjin intervention, including systematic review (SR), randomized clinical trial (RCT), nonrandomized controlled clinical studies (CCS) (quasirandomized clinical trial or observational studies such as cohort or case-control study), case series (CS), and case report (CR), which included Baduanjin as the intervention for any disease/condition or healthy participants. Any type of Baduanjin, regardless of its style or training method, was included.

Anecdotes, newsletters, cross-sectional studies, study protocols, and duplicate publications were excluded. Reports published as abstracts and lack of research on basic information on Baduanjin interventions were excluded. Neither the reviews irrelevant to Baduanjin intervention nor the studies of complex interventions using Baduanjin as one of the intervention components, which did not provide a detailed description of Baduanjin intervention, were also excluded.

### 2.3. Literature Screening and Data Extraction

Two authors (YYY and JJ) independently screened the article titles and abstracts based on the inclusion and exclusion criteria, downloaded the full text for further screening, and also classified all eligible clinical studies according to their study designs. The screening and extraction results of the two research members were compared, and if there was any uncertainties or discrepancies, a third author (YZ) was consulted.

A structured data extraction form was designed by two authors (YYY and JJ) to extract data from the included articles independently. The form consisted of the following sections:

1. *Basic Information*. Including article title, author, country/region, year of publication, study design, publication type, and funding information if available
2. *Disease/Condition*. According to the International Classification of Diseases, 11th Revision (ICD-11), the names of directly extracted diseases/conditions were divided into different categories [11]. In addition, the sample size of subjects included in each clinical study was also recorded.
3. *Baduanjin Intervention*. We extracted the style of Baduanjin, single intervention/combined intervention, qualification of the instructor, practice methods, frequency, and treatment duration. If Baduanjin was used in combination with other therapies, other therapies were extracted. Besides, the interventions of the control group were specifically recorded.
4. *Outcomes and Conclusions*. We extracted all the outcomes directly and classified them into different categories. If shedding and adverse reactions were reported, the results were extracted if available. We also summarized the conclusions (positive, negative, or unclear) of the overall authors. “Positive” was defined if the study achieved its objective and statistically favored Baduanjin; “negative” was defined if the study failed to reach its objective or did not favor Baduanjin; “unclear” was defined if the study objective was unclear or the conclusions were inconclusive.

### 2.4. Methodological Quality Assessment

Using the risk of bias (ROB) tool in the Review Manager (RevMan) software (Version 5.3.5, Nordic Cochrane Center, Cochrane Collaboration, Copenhagen, Denmark) and referring to the ROB assessment criteria in the Cochrane Handbook [12], the included randomized controlled trials were evaluated “low risk of bias,” “unclear risk of bias,” and “high risk of bias” from these aspects that included random sequence generation (selection bias), allocation concealment (selection bias), blinding of participants and personnel (performance bias), blinding of outcome assessment (detection bias), incomplete outcome data (attrition bias), selective reporting (reporting bias), and other bias.

### 2.5. Data Analysis

The extracted data were analyzed using the SPSS software (Version 22.0, SPSS, Inc., Chicago, IL, USA) and were presented by counts, percentage, and frequency.

## 3. Results

### 3.1. Selection of Studies

**Figure 1:**
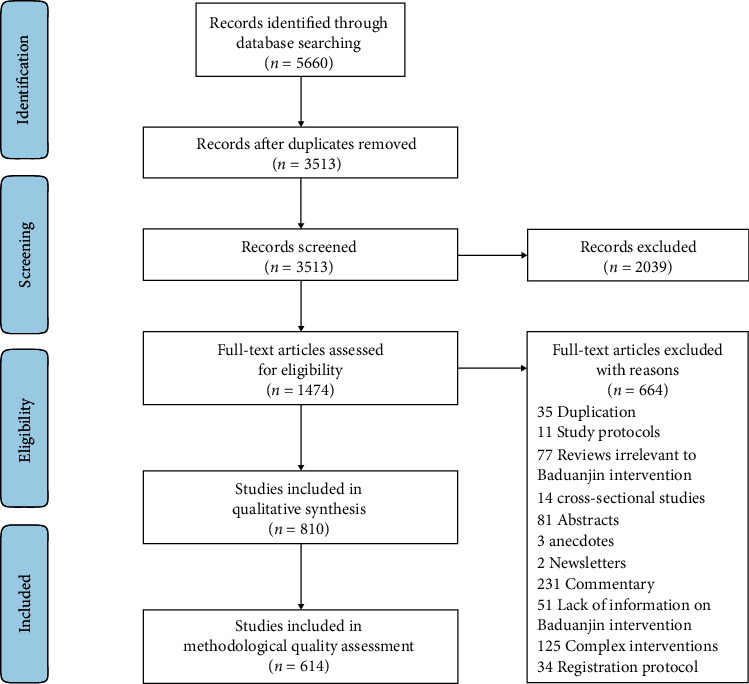
Flow diagram. Presentation of the procedure of study searching and selection with numbers of articles at each stage.

Flow of the study search and selection process is shown in Figure 1. 5660 articles were initially identified through electronic retrieval. After removing duplicates, 3513 articles were obtained, and the titles and abstracts of these literatures were screened to exclude 2039 articles that did not meet the inclusion criteria. According to the inclusion and exclusion criteria, the remaining 1474 articles were selected for full-text download, and 664 articles were excluded again, and then, 810 studies were left for further analysis. The characteristics of included clinical studies on Baduanjin are shown in Table S1.

### 3.2. General Characteristics of Included Studies

**Figure 2:**
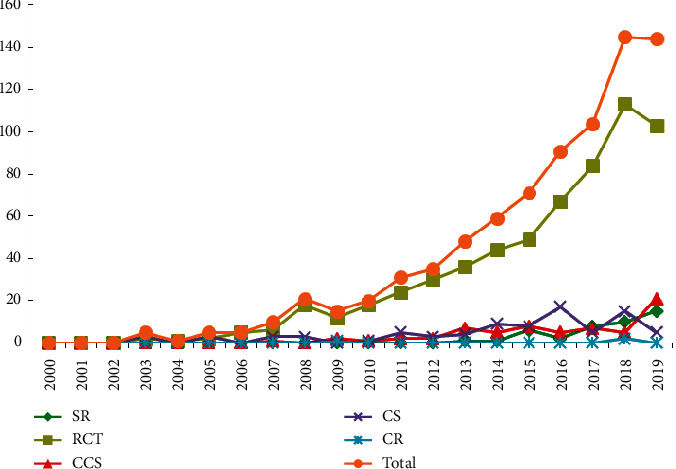
Study designs over time in the numbers of published clinical studies on Baduanjin. Abbreviations: SR, systematic review; RCT, randomized clinical trial; CCS, nonrandomized controlled clinical studies (quasirandomized clinical trial or observational studies such as cohort or case-control studies); CS, case series; CR, case report.

In the last 20 years, the publication of clinical studies on Baduanjin generally increased with years (Figure 2). Among the 810 studies, 737 (91.0%) were published in Chinese and 73 (9.0%) in English. Depending on the type of article published, it included 655 (80.9%) journal articles, 17 (2.1%) conference papers, and 138 (17.0%) master's and doctoral dissertations. Furthermore, 332 (41.0%) studies reported the support from a foundation, most of which were funded by government.

The included studies covered almost all types of clinical research, including 43 (5.3%) systematic reviews, 614 (75.8%) randomized controlled trials, 66 (8.1%) nonrandomized controlled clinical studies, 84 (10.4%) case series, and 3 (0.4%) case reports. The 810 studies were implemented in 9 countries/regions, and the majority of studies (789/810, 97.4%) were implemented in Chinese Mainland, followed by Hong Kong (10/810, 1.2%) and Taiwan (5/810, 0.6%) (Table 1).

### 3.3. Disease/Condition Categories

Of the 810 articles included, 152 (18.8%) studies explored the health-promoting effects of Baduanjin on healthy participants; 19 (2.3%) studies explored the health-recovery effects of Baduanjin on subhealthy participants; the remaining 639 (78.9%) studies observed the efficacy and safety of Baduanjin in 81 clinical diseases/conditions. According to ICD-11 categorization, Baduanjin was most commonly used in diseases of the musculoskeletal system or connective tissue, endocrine, nutritional or metabolic diseases, and diseases of the circulatory system (Table 2). The top 10 diseases/conditions included diabetes, chronic obstructive pulmonary disease (COPD), hypertension, low back pain, neck pain, stroke, coronary heart disease, cognitive impairment, insomnia, and osteoporosis or osteopenia (Table 3).

### 3.4. Sample Size

Excluding systematic reviews and case reports, the study sample size of 764 articles including randomized controlled trials, nonrandomized controlled clinical studies, and case series was analyzed. The average sample size of all studies was 82 cases, which belonged to small sample size studies, and the sample size fluctuated greatly. The sample size varies significantly between different types of studies, and the sample size fluctuations between the same types of studies are also large (Table 4).

### 3.5. Baduanjin Intervention

We removed systematic reviews to avoid duplication of information and performed statistical analysis on the Baduanjin intervention in 767 articles including randomized controlled trials, nonrandomized controlled clinical studies, case series, and case reports.  Table 5 shows the Baduanjin styles applied in the 767 clinical studies, of which 332 (43.3%) studies identified the style of Baduanjin, and the style of State General Administration of Sport of China in 2003 was the most commonly used.

324 (42.2%) studies used Baduanjin alone as intervention, while Baduanjin was applied in combination with other therapies in the remaining 443 (57.8%) studies, which include conventional medications, acupuncture, herbal medications, health education, diet and lifestyle guidance, psychological, and other physical therapies. 440 (57.4%) studies reported that participants learned Baduanjin under the guidance of instructors, but rarely described the qualification of instructors. There were 339 (44.2%) studies that mentioned the practice methods of Baduanjin, in which participants practiced Baduanjin under the guidance and supervision of instructors (132/767, 17.2%), or by themselves at home with the support of videos (71/767, 9.3%), or both of the two methods (136/767, 17.7%).

Baduanjin practice in 747 (97.4%) studies varied from 5 minutes [13] to 120 minutes [14] each session, with an average of 35 minutes. Most studies practice 1 or 2 times a day, 3–5 days per week, and five 30 minutes sessions per week (73/767, 9.5%) were most popular. The duration of Baduanjin intervention varied from 5 days [15] to 3 years [16], with an average of 18 weeks, and the most common duration was 12 weeks (261/767, 34.0%), followed by 24 weeks (159/767, 20.7%).

### 3.6. Control Intervention

The control interventions of 680 randomized clinical trials and nonrandomized controlled clinical studies were statistically analyzed, and the most common intervention was the routine treatment plan (234/680, 34.4%), followed by blank control (181/680, 26.6%), exercise (134/680, 19.7%), health education (65/680, 9.6%), medications (58/680, 8.5%), and other traditional Chinese medicine methods (including Tuina, acupuncture, and herbal medications). Among the 134 studies using exercise as a control intervention, the most commonly used mode was walking (49/680, 7.2%), followed by traditional Chinese exercise such as Tai Chi Chuan, Wuqinxi, Yijinjing, and Liuzijue (31/680, 4.6%), jogging (12/680, 1.8%), broadcast calisthenics (7/680, 1.0%), and other aerobic and resistance exercises.

### 3.7. Outcomes

In addition to systematic reviews, statistical analysis was conducted on the primary outcomes of 767 clinical studies. The most common outcomes were related to laboratory tests, such as blood glucose and blood lipids. Second, the related symptom scales were evaluated, such as the visual analogue scale (VAS), JOA score, Oswestry disability index (ODI), COPD assessment test (CAT), Seattle Angina Questionnaire (SAQ), Pittsburgh Sleep Quality Index (PSQI), and Kupperman score. The third was the quality of the life scale, such as the Diabetes-Specific Quality of Life Scale (DSQL), SF-36, and World Health Organization Quality of Life Brief Scale (WHOQOL-BREF). There were also assessments of physiological functions including body mass index (BMI) and balance ability. As well as psychological assessment, such as the self-rating anxiety scale (SAS), self-rating depression scale (SDS), Hamilton depression scale (HAMD), Profile of Mood States (POMS), and Symptom Checklist 90 (SCL-90).

Except for 43 systematic reviews, only 70 (9.1%) of the remaining 767 studies mentioned records of adverse reactions, of which 7 (0.9%) studies reported that there were adverse events in detail, such as muscle and joint pain caused by exercise, fatigue, dizziness, chest tightness, palpitation, shortness of breath, and so on. There were no serious adverse events related to Baduanjin intervention.

After removing 3 case reports again, only 218 (28.5%) of the remaining 764 studies mentioned that the reasons for shedding included patients who were lost to follow-up, failed to complete the test, excluded from the study protocol, and combined other diseases or deaths that were not significantly related to Baduanjin intervention.

### 3.8. Methodological Quality Assessment

**Figure 3:**
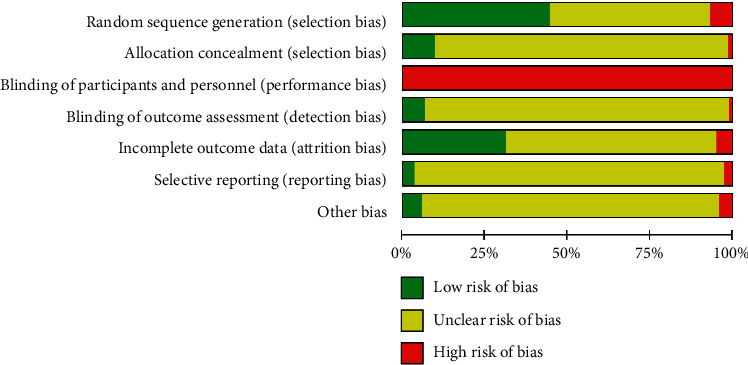
Risk of bias graph: review authors' judgments about each risk of the bias item presented as percentages across all included studies.

The methodological quality of 614 randomized controlled trials was evaluated using the ROB tool in RevMan 5.3.5, which suggests moderate quality (Figure 3). Only less than half of the studies have identified low-risk random sequence generation methods (273/614, 44.5%), and there were still a few studies that used high-risk random methods such as grouping by odd and even numbers of hospital records (41/614, 6.7%). The method of allocation concealment was clearly identified in only 57 (9.3%) studies; so, the selectivity bias was significant in the included studies. Because Baduanjin intervention could not blind participants, bias was high risk in all studies. Only 37 (6.0%) studies explicitly blinded the outcome evaluators, 192 (31.3%) studies completely reported the approach to case dropout and missing data, and very few studies mentioned protocol registration and published the results in full (19/614, 3.1%), so the overall quality of the study included in the detection bias, attrition bias, and reporting bias was not high. Only a few studies have fully reported protocol registration, clinical ethics review, and clinical trial procedures, which were sufficient to exclude other risks of bias (32/614, 5.2%).

## 4. Discussion

This systematic review is a comprehensive qualitative analysis from 2000 to 2019 of the clinical evidence on Baduanjin for healthcare, treatment, and rehabilitation, including systematic reviews, randomized controlled trials, nonrandomized controlled clinical trials, case series, and case reports. Our findings indicate that after 2000, the number of clinical studies on Baduanjin has shown an increasing trend, especially higher level evidence such as RCTs. This may be related to the fact that the Health Qigong Management Center of State General Administration of Sport of China organized and edited Health Qigong including Baduanjin in 2003 and promoted it vigorously throughout the country. Of note, nearly half of studies are supported by the fund, which also reminds the national government and medical institutions to attach importance to the clinical benefits of Baduanjin.

The clinical research of Baduanjin not only involves the physical and mental state of healthy/subhealthy people but also includes a wide range of diseases/conditions such as diabetes, chronic obstructive pulmonary disease (COPD), hypertension, lower back pain, neck pain, stroke, coronary heart disease, cognitive impairment, insomnia, osteoporosis or osteopenia, and so on. Most diseases are diseases of the musculoskeletal system or connective tissue, endocrine, nutritional or metabolic diseases, diseases of the circulatory system, diseases of the nervous system, and diseases of the respiratory system, which may be related to the physiological and biomechanical processes Baduanjin training influences. Baduanjin is a low-intensity aerobic exercise, which is characterized by a slow coordinated posture combined with musculoskeletal stretching movements, meditation minds, and breathing techniques. Several recent research studies have shown that practicing Baduanjin can relieve musculoskeletal pain [17–19], adjust blood pressure, glucose, and lipid [4, 8, 20], enhance cardiopulmonary function [7, 21], and improve sleep quality by relaxing the mental state and regulating breathing [22, 23].

It is of great significance to fully describe the details of interventions in clinical trials not only to provide solid evidence for clinical application but also to provide a detailed reference for further repeated trial operations [24]. This review shows that most of the included clinical trials did not adequately describe the style and form, session, frequency, duration, learning methods, and qualification of instructors of Baduanjin intervention. In this review, the included clinical trials of Baduanjin have a wide range of exercise intensity, with an average time of 35 minutes per session. Most studies practice 1 or 2 times a day, 3–5 days per week, and five 30 minutes sessions per week were most popular. The requirement of moderate-intensity exercise may be the reason why most studies have obtained positive results. On this basis, we recommend standardizing the design, conduct, and reporting of Baduanjin intervention to ensure that it is better implemented and evaluated in clinical trials.

Most of the control interventions in the clinical study of Baduanjin are without exercise or other exercise. Clinically, the evidence of health benefits of exercise is accurate [25], and Baduanjin also shows obvious advantages compared with no exercise. However, when compared with other exercises such as walking and jogging, the results of the study are significantly different. This may be because the study design of different clinical studies is different, and we cannot combine them for further quantitative analysis.

The vast majority of clinical trials included in this study obtained positive results, showing that Baduanjin has certain benefits in prevention, treatment, or rehabilitation. However, most of the included studies are small-sample studies, and the methodological quality of randomized controlled trials is low. Therefore, according to the current clinical evidence, the clinical benefits of Baduanjin still need to be further verified by random controlled trials with a large sample size and strict design. Besides, patient participation and compliance also have a certain impact on the research results. In this study, 218 (28.5%) clinical trials reported that the reason for shedding was mainly related to the low compliance of patients. Thus, how to improve patient compliance in subsequent clinical trials is worthy of serious consideration and design.

There is currently no review to systematically assess the frequency and quality of adverse event reports related to the clinical trial of Baduanjin. Only a few studies mentioned mild symptoms such as muscle and joint pain caused by exercise, and most of them relieved or disappeared quickly after stopping exercise and resting. Similarly, due to poor and inconsistent reports of adverse events, this review also failed to clearly draw conclusions about the safety of Baduanjin, but none of the existing studies reported serious adverse events related to Baduanjin.

Inevitably, there are some limitations in this review. First, subjected to the condition that our search language is limited to Chinese and English, and the search results also show that most of the Baduanjin studies were published in Chinese by Chinese researchers, but it cannot be ruled out that there may be a small number of clinical studies published in other languages, such as Japanese and Korean. Second, although most studies have obtained positive results, its methodological quality is low; so publication bias cannot be ruled out, and it also limits the level of evidence for bibliometric evaluation. Third, we focus on using qualitative analysis methods to describe the clinical research trends and characteristics of Baduanjin in the past 20 years; however, there is no quantitative analysis of a specific disease, and it is impossible to provide specific evidence-based evidence for the application of Baduanjin to a certain disease.

We make the following suggestions for further clinical studies on Baduanjin. First of all, we should improve and standardize the reporting of Baduanjin interventions in clinical trials. According to CONSORT 2010 statement [26], we recommend that the style and form, session, frequency, duration, learning methods, practicing methods, instructor qualification, participant compliance, and follow-up of Baduanjin intervention can be clarified in the research protocol. In addition, except for the fact that Baduanjin intervention cannot be blinded, which leads to performance bias, other aspects of the randomized controlled trials should be designed strictly. What is more, we can try to explore the relationship between the intensity and/or duration of Baduanjin and its effect on specific diseases and optimize the dose-response to further provide evidence for the clinical application of Baduanjin. Finally, in this review, we found that the majority of included clinical trials were not registered for prospective trials. Therefore, we recommend that trials should be registered in public clinical trial registries to prevent reporting bias.

## 5. Conclusions

This review, based on various diseases/conditions literature available in the past 20 years, suggests that the clinical studies of Baduanjin have a substantial quantity and evidence base. Although most studies report positive effects of Baduanjin on prevention, treatment, and rehabilitation, there are significant differences among different studies in the specific intervention measures such as style, intensity, duration, learning, and practice methods, which need to be further standardized and unified. Furthermore, because the methodological quality of the current research is low, it is recommended that high-quality designed and reporting studies should be conducted in the future to further validate the clinical benefits of Baduanjin.

## Figures and Tables

**Table 1:** Number of clinical studies on Baduanjin conducted in different countries/regions (*n* = 810).

Country/region	Study design (number of studies)					Total (%)
	SR	RCT	CCS	CS	CR	
Chinese Mainland	35	603	66	82	3	789 (97.4)
Hong Kong	4	6	0	0	0	10 (1.2)
Taiwan	0	4	0	1	0	5 (0.6)
USA	1	0	0	0	0	1 (0.1)
Australia	1	0	0	0	0	1 (0.1)
Brazil	1	0	0	0	0	1 (0.1)
Chile	1	0	0	0	0	1 (0.1)
Spain	0	1	0	0	0	1 (0.1)
Macao	0	0	0	1	0	1 (0.1)

SR, systematic review; RCT, randomized clinical trial; CCS, nonrandomized controlled clinical studies (quasirandomized clinical trial or observational studies such as cohort or case-control studies); CS, case series; CR, case report; USA, United States of America.

**Table 2:** Clinical trials of Baduanjin organized by prevalence of disease categories based on ICD-11 classifications.

Chapter	Blocks	Disease/conditions (ICD-11 codes)	No. of study^*∗*^
15	FA00–FC0Z	Diseases of the musculoskeletal system or connective tissue	124
05	5A00–5D46	Endocrine, nutritional, or metabolic diseases	124
11	BA00–BE2Z	Diseases of the circulatory system	114
08	8A00–8E7Z	Diseases of the nervous system	65
12	CA00–CB7Z	Diseases of the respiratory system	63
06	6A00–6E8Z	Mental, behavioral, or neurodevelopmental disorders	46
02	2A00–2F9Z	Neoplasms	32
16	GA00–GC8Z	Diseases of the genitourinary system	24
07	7A00–7B2Z	Sleep-wake disorders	17
13	DA00–DE2Z	Diseases of the digestive system	10
14	EA00–EM0Z	Diseases of the skin	6
04	4A00–4B4Z	Diseases of the immune system	5
01	1A00–1H0Z	Certain infectious or parasitic diseases	4
09	9A00–9E1Z	Diseases of the visual system	2

ICD-11, International Classification of Diseases, 11th Revision. ^*∗*^Some systematic reviews involved more than one type of diseases or conditions.

**Table 3:** Top 10 diseases/conditions included in clinical studies on Baduanjin.

Disease/Condition	Study design (number of studies)					Total (%)
	SR^*∗*^	RCT	CCS	CS	CR	
Diabetes	6	79	6	6	0	97 (12.0)
COPD	4	54	3	0	0	61 (7.5)
Hypertension	6	35	5	2	0	48 (5.9)
Low back pain	2	38	2	0	0	42 (5.2)
Neck pain	1	34	1	1	0	37 (4.6)
Stroke	3	28	1	1	0	33 (4.1)
Coronary heart disease	3	18	2	1	0	24 (3.0)
Cognitive impairment	3	17	0	0	0	20 (2.5)
Insomnia	1	14	0	2	0	17 (2.1)
Osteoporosis or osteopenia	1	14	0	0	0	15 (1.9)

SR, systematic review; RCT, randomized clinical trial; CCS, nonrandomized controlled clinical studies (quasirandomized clinical trial or observational studies such as cohort or case-control studies); CS, case series; CR, case report; COPD, chronic obstructive pulmonary disease. ^*∗*^Some systematic reviews involved more than one type of diseases or conditions.

**Table 4:** Sample size in the clinical study on Baduanjin (*n* = 764).

Study design	No. of study	Sample size		
		Minimum	Maximum	Mean ± standard deviation
RCT	614	16	1973	81.16 ± 86.32
CCS	66	12	1721	109.98 ± 208.67
CS	84	12	500	69.45 ± 68.84

RCT, randomized clinical trial; CCS, nonrandomized controlled clinical studies (quasirandomized clinical trial or observational studies such as cohort or case-control studies); CS, case series.

**Table 5:** Baduanjin styles applied in 767 clinical studies including RCT, CCS, CS, and CR.

Baduanjin style	No. of study	Frequency (%)
Unspecified style	445	58.0
The style of State General Administration of Sport of China in 2003	264	34.4
Homemade style	18	2.3
Others	12	1.6
The style of Chinese Health Qigong Association	11	1.4
Deng Tietao style	9	1.2
The style of Chinese Traditional Sport Health Preservation	5	0.7
The style of Beijing Sport University	3	0.4

RCT, randomized clinical trial; CCS, nonrandomized controlled clinical studies (quasirandomized clinical trial or observational studies such as cohort or case-control studies); CS, case series; CR, case report.

## Data Availability

All data generated or analyzed during this study are included within this article. Raw data are available from the corresponding author upon request.

## Supplementary Materials

Supplementary Materials Table S1: characteristics of included clinical studies on Baduanjin.

## References

[1] Health Qigong Management Center of General Administration of Sport of China (2007). *Chinese Health Qigong: Ba DuanJin*.

[2] Zou L., Pan Z., Yeung A. (2017). A review study on the beneficial effects of Baduanjin. *The Journal of Alternative and Complementary Medicine*.

[3] Li M. Y., Fang Q. Y., Li J. Z. (2015). The effect of Chinese traditional exercise-Baduanjin on physical and psychological well-being of college students: a randomized controlled trial. *PLoS One*.

[4] Song G., Chen C., Zhang J., Chang L., Zhu D., Wang X. (2018). Association of traditional Chinese exercises with glycemic responses in people with type 2 diabetes: a systematic review and meta-analysis of randomized controlled trials. *Journal of Sport and Health Science*.

[5] Yu T. T., Yu X. L., Zeng L. M., Zhou X., Zhao R. H. (2014). Baduanjin for diabetes: a systematic review. *Chinese Journal of Evidence-Based Medicine*.

[6] Yang J. P., Liu J. Y., Lv W. L., Meng L. N., Zhao H. (2015). Meta-analysis on effects of Baduanjin on patients with type 2 diabetes mellitus. *China Journal of Traditional Chinese Medicine and Pharmacy*.

[7] Tong H. X., Liu Y. H., Zhu Y. T., Zhang B. L., Hu J. Q. (2019). The therapeutic effects of qigong in patients with chronic obstructive pulmonary disease in the stable stage: a meta-analysis. *BMC Complementary and Alternative Medicine*.

[8] Xiong X., Wang P., Li S., Zhang Y., Li X. (2015). Effect of Baduanjin exercise for hypertension: a systematic review and meta-analysis of randomized controlled trials. *Maturitas*.

[9] Gai T. T., Fan W., Gao M. X., Cui Y., Wang Y. (2019). Effect of Baduanjin on rehabilitation of patients with coronary heart disease:A meta-analysis of 8 randomized controlled trials. *TMR Integrative Nursing*.

[10] General Office of National Health Commission (2020). *Notice on issuing guidelines for rehabilitation of traditional Chinese medicine in the recovery period of new coronary virus Pneumonia (Trial) (in Chinese). [EB/OL]. National Health Commission of the People’s Republic of China*.

[11] World Health Organization (2019). *International Statistical Classification of Diseases, 11th Revision for Mortality and Morbidity Statistics (ICD-11 MMS Version 2019)*.

[12] Lefebvre C., Manheimer E., Glanville J., Higgins J. P., Green S. (2008). Chapter 6: searching for studies. *Cochrane Handbook for Systematic Reviews of Interventions*.

[13] Wang Q. R. (2018). *A Clinical Study: Effect of The Ba DuanJin for Treating Nonspecific Low Back Pain*.

[14] Li Y., Peng X. L., Liu L., He L. Y., Liu F. (2018). Effect of Baduanjin exercise on mental health in patients with polycystic ovary syndrome. *Hunan Journal of Traditional Chinese Medicine*.

[15] Wang L. (2018). Effects of Baduanjin on treatment and rehabilitation of patients with insomnia (in Chinese). *Psychological Doctor*.

[16] Zhang S. W., Cheng X. F., Yu S., Dong Q., He Y. H. (2013). Clinical observation of inverted walk combined with Baduanjin exercise on the prevention of the recurrence of lumbar disc herniation. *Hebei Journal of Traditional Chinese Medicine*.

[17] Zou L. Y., Yeung A., Quan X. F., Boyden S. D., Wang H. R. (2018). A systematic review and meta-analysis of mindfulness-based (Baduanjin) exercise for alleviating musculoskeletal pain and improving sleep quality in people with chronic diseases. *International Journal of Environmental Research and Public Health*.

[18] Li H., Ge D., Liu S. (2019). Baduanjin exercise for low back pain: a systematic review and meta-analysis. *Complementary Therapies in Medicine*.

[19] Zeng Z.-p., Liu Y.-b., Fang J., Liu Y., Luo J., Yang M. (2020). Effects of Baduanjin exercise for knee osteoarthritis: a systematic review and meta-analysis. *Complementary Therapies in Medicine*.

[20] Pan M., Deng Y., Zheng C. (2019). The effects of Qigong exercises on blood lipid profiles of middle-aged and elderly individuals: a systematic review and network meta-analysis. *European Journal of Integrative Medicine*.

[21] Xiao X. L., Wang J., Gu Y. M., Cai Y. F., Ma L. X. (2018). Effect of community based practice of Baduanjin on self-efficacy of adults with cardiovascular diseases. *PLoS One*.

[22] Jiang Y. H., Tan C., Yuan S. (2017). Baduanjin Exercise for Insomnia: A Systematic Review and Meta-Analysis. *Behavioral Sleep Medicine*.

[23] Chen M.-C., Liu H.-E., Huang H.-Y., Chiou A.-F. (2011). The effect of a simple traditional exercise programme (Baduanjin exercise) on sleep quality of older adults: a randomized controlled trial. *International Journal of Nursing Studies*.

[24] Antonishen K. (2015). Exercise mode heterogeneity among reported studies of the qigong practice Baduanjin. *Journal of Bodywork and Movement Therapies*.

[25] Murphy M. H., Lahart I., Carlin A., Murtagh E. (2019). The effects of continuous compared to accumulated exercise on health: a meta-analytic review. *Sports Medicine*.

[26] Schulz K. F., Altman D. G., Moher D. (2010). CONSORT 2010 statement: updated guidelines for reporting parallel group randomised trials. *BMJ*.

