# Supplementary material for: Characteristic of Clinical Studies on Baduanjin during 2000–2019: A Comprehensive Review
Source: Evid Based Complement Alternat Med. 2020 Oct 16;2020:4783915. doi: 10.1155/2020/4783915 (PMC7603575; doi:10.1155/2020/4783915)
Supplement: Supplementary Materials — Table S1: characteristics of included clinical studies on Baduanjin. [file 4783915.f1.docx]

**Table S1. Characteristics of included clinical studies on Baduanjin.**

| **Study ID** | **Study design** | **Country** | **Disease/conditions** | **Baduanjin Intervention** | **Outcomes** |
| --- | --- | --- | --- | --- | --- |
| Zhong MY 2018 | CR | Chinese Mainland | Stress incontinence | Unspecified style, 30 minutes per time, 5 times per week, for 4 weeks. | Symptom |
| Pan J 2009 | CR | Chinese Mainland | Neurasthenia | Unspecified style, for 12 weeks. | Symptom |
| Shi ZC 2018 | CR | Chinese Mainland | Depression | Unspecified style, 30 minutes per time, 3 to 5 times per week, for 8 weeks. | Psychological: depression |
| Wang LY 2014 | CCS | Chinese Mainland | Health promotion | Unspecified style, 90 minutes per time, twice per week, for 12 weeks. | Physical performance |
| Zhou KK 2019 | CCS | Chinese Mainland | Health promotion | The style of State General Administration of Sport of Chinese Mainland in 2003, practiced under the instruction of a coach for 3 days before the beginning of study to grasp the skills and key points, 15 minutes per time, 3 times per week, for 5 weeks. | Symptom; QOL: SF-36; Psychological: anxiety |
| Chen YF 2019 | CCS | Chinese Mainland | Type 2 diabetes | The style of Chinese Traditional Sport Health Preservation, supervised by qualified nurse, 35 to 40 minutes per time, 5 times per week, for 24 weeks. | Laboratory tests: FPG, HbA1c |
| Yang JL 2016 | CCS | Chinese Mainland | Type 2 diabetes | The style of Chinese Traditional Sport Health Preservation, supervised by qualified instructor, 35 to 40 minutes per time, 5 times per week, for 24 weeks. | Laboratory tests: FPG, HbA1c |
| Yan SX 2012 | CCS | Chinese Mainland | Schizophrenia | The style of State General Administration of Sport of Chinese Mainland in 2003, supervised by qualified instructor, 30 minutes per session, twice per day, 5 times per week, for 12 weeks. | Laboratory tests: FPG, HbA1c, blood lipid |
| Jia HY 2013 | CCS | Chinese Mainland | Falls Prevention | Unspecified style, 60 minutes per time, 4 times per week, for 10 weeks. | Physical performance: strength, balance |
| Fan GQ 2014 | CCS | Chinese Mainland | Hypertension | Unspecified style, 30 minutes per session, twice per day, 2 to 3 times per week, for 12 weeks. | Physical performance: blood pressure; QOL: SF-36 |
| Lan H 2019 | CCS | Chinese Mainland | Breast cancer | The style of State General Administration of Sport of Chinese Mainland in 2003, supervised by qualified instructor, 90 minutes per time, 3 times per week, for 12 weeks. | Laboratory tests: immunity; Symptom |
| Wu ZH 2018 | CCS | Chinese Mainland | Bowel cancer | Unspecified style, supervised by qualified instructor, 30 minutes per time, 5 times per week, for 8 weeks. | Symptom |
| Chen W 2013 | CCS | Chinese Mainland | Health promotion | The style of State General Administration of Sport of Chinese Mainland in 2003, supervised by qualified instructor, 60 minutes per time, 6 times per week, for 20 weeks. | Symptom |
| He LP 2019 | CCS | Chinese Mainland | Health promotion | Unspecified style, supervised by qualified instructor, 60 minutes per time, 4 times per week, for 12 weeks. | Physical performance: flexibility, balance |
| Lin JW 2017 | CCS | Chinese Mainland | Chronic kidney disease | Unspecified style, 20 minutes per time, 3 times per week, for 12 weeks. | Laboratory tests; Physical performance |
| Si LY 2009 | CCS | Chinese Mainland | Climacteric syndrome | Unspecified style, supervised by qualified instructor, 10 to 15 minutes per session, twice per day, 5 times per week, for 12 weeks. | Laboratory tests: blood lipid; Physical performance: BMI; Symptom |
| Shi XM 2017 | CCS | Chinese Mainland | Coronary heart disease | The style of State General Administration of Sport of Chinese Mainland in 2003, 60 minutes per time, 3 times per week, for 12 weeks. | Laboratory tests; Physical performance: cardiopulmonary function |
| Chen YQ 2015 | CCS | Chinese Mainland | Hypertension | Unspecified style, 60 minutes per session, twice per day, 3 to 4 times per week, for 12 weeks. | Physical performance: blood pressure; Symptom |
| Liu XY 2015 | CCS | Chinese Mainland | Falls Prevention | Unspecified style, supervised by qualified instructor, 30 to 40 minutes per time, 5 times per week, for 12 weeks. | Physical performance: balance |
| Song BX 2019 | CCS | Chinese Mainland | Breast cancer | The style of State General Administration of Sport of Chinese Mainland in 2003, supervised by qualified instructor, 90 minutes per time, 3 times per week, for 12 weeks. | Laboratory tests: BMD, cardiopulmonary function |
| Yang QC 2019 | CCS | Chinese Mainland | Health promotion | Unspecified style, 60 minutes per time, 5 times per week, for 12 weeks. | Symptom |
| Cheng QY 2019 | CCS | Chinese Mainland | Type 2 diabetes | Unspecified style, 60 minutes per time, once per week, for 12 weeks. | Laboratory tests: FPG, HbA1c; QOL: SF-36 |
| Ma Y 2019 | CCS | Chinese Mainland | Stroke | Unspecified style, supervised by qualified instructor, 40 minutes per session, twice per day, 5 times per week. | Symptom |
| Cao J 2013 | CCS | Chinese Mainland | Chronic kidney disease | The style of State General Administration of Sport of Chinese Mainland in 2003, 40 minutes per time, 5 times per week, for 8 weeks. | QOL: WHOQOL-BREF; Psychological: anxiety, depression |
| Shi Y 2019 | CCS | Chinese Mainland | Tuberculosis | Unspecified style, supervised by qualified instructor, for 24 weeks. | Laboratory tests; Symptom; QOL: SF-36 |
| Cai HL 2018 | CCS | Chinese Mainland | Hypertension | Unspecified style, 25 minutes per time, 5 times per week, for 8 weeks. | Physical performance: blood pressure |
| Chen Y 2016 | CCS | Chinese Mainland | Chronic bronchitis | Unspecified style, 20 minutes per time, 5 times per week. | Laboratory tests |
| Cui XQ 2018 | CCS | Chinese Mainland | Type 2 diabetes | Unspecified style, supervised by qualified instructor, 30 minutes per time, 5 times per week, for 24 weeks. | Laboratory tests: FPG, HbA1c, blood lipid |
| Wei N 2019 | CCS | Chinese Mainland | Parkinson disease | Unspecified style, supervised by qualified instructor, 60 minutes per time, 5 times per week. | Symptom; QOL; Psychological: depression |
| Liu PF 2014 | CCS | Chinese Mainland | Hypertension | Unspecified style, supervised by qualified instructor, 30 minutes per session, twice per day, 5 times per week, for 12 weeks. | Laboratory tests: HbA1c, blood lipid; Physical performance: blood pressure, BMI |
| Lei CY 2019 | CCS | Chinese Mainland | COPD | Unspecified style, supervised by qualified instructor, 30 minutes per time, 5 times per week, for 48 weeks. | Laboratory tests; Symptom |
| Lou H 2010 | CCS | Chinese Mainland | Health promotion | Unspecified style, 50 minutes per time, 7 times per week, for 16 weeks. | Physical performance |
| Liu FC 2019 | CCS | Chinese Mainland | Parkinson disease | Unspecified style, supervised by qualified instructor, 30 minutes per time, 7 times per week. | Symptom; Psychological: anxiety, depression |
| Liu LL 2017 | CCS | Chinese Mainland | Health promotion | Unspecified style, 30 minutes per session, twice per day, 4 times per week, for 24 weeks. | Psychological |
| Wang JY 2013 | CCS | Chinese Mainland | Neck pain | Unspecified style, supervised by qualified instructor, 30 minutes per time, 5 times per week, for 24 weeks. | Symptom: VAS; QOL: SF-36 |
| Xie F 2019 | CCS | Chinese Mainland | Addiction | Unspecified style, 20 to 30 minutes per session, twice per day, 5 times per week, for 24 weeks. | Symptom; Psychological: anxiety, depression |
| Guo Y 2019 | CCS | Chinese Mainland | Health promotion | The style of State General Administration of Sport of Chinese Mainland in 2003, supervised by qualified instructor, 15 minutes per time, 7 times per week, for 10 weeks. | Laboratory tests |
| Guo Y 2016 | CCS | Chinese Mainland | Health promotion | The style of State General Administration of Sport of Chinese Mainland in 2003, supervised by qualified instructor, 15 minutes per time, 7 times per week, for 10 weeks. | Laboratory tests |
| Guo Y 2017 | CCS | Chinese Mainland | Health promotion | The style of State General Administration of Sport of Chinese Mainland in 2003, supervised by qualified instructor, 15 minutes per time, 7 times per week, for 10 weeks. | Laboratory tests |
| Guo Y 2019 | CCS | Chinese Mainland | Health promotion | The style of State General Administration of Sport of Chinese Mainland in 2003, supervised by qualified instructor, 15 minutes per time, 7 times per week, for 10 weeks. | Laboratory tests |
| Song R 2018 | CCS | Chinese Mainland | Coronary heart disease | Unspecified style, 30 to 40 minutes per time, 4 times per week, for 12 weeks. | Symptom; QOL; Psychological: anxiety |
| Liu FC 2019 | CCS | Chinese Mainland | Parkinson disease | Unspecified style, supervised by qualified instructor, 30 minutes per time, 7 times per week. | Symptom |
| Ma WB 2016 | CCS | Chinese Mainland | Health promotion | Unspecified style, supervised by qualified instructor, 30 minutes per time, 7 times per week, for 12 weeks. | Physical performance: balance |
| Cao Y 2015 | CCS | Chinese Mainland | Type 2 diabetes | Unspecified style, 60 minutes per time, 3 times per week, for 12 weeks. | Laboratory tests: FPG, HbA1c |
| Zhang W 2015 | CCS | Chinese Mainland | Health promotion | Unspecified style, supervised by qualified instructor, 30 minutes per session, twice per day, 5 times per week, for 10 weeks. | Physical performance: pulmonary function |
| Song JC 2015 | CCS | Chinese Mainland | Health promotion | Unspecified style, 30 minutes per time, 4 times per week, for 12 weeks. | Symptom; QOL; Psychological |
| Ma HJ 2011 | CCS | Chinese Mainland | Health promotion | Unspecified style, supervised by qualified instructor, 45 minutes per time, 5 times per week, for 12 weeks. | Psychological |
| Wang R 2017 | CCS | Chinese Mainland | Health promotion | Unspecified style, 40 minutes per time, 3 times per week, for 12 weeks. | Physical performance |
| Ding X 2017 | CCS | Chinese Mainland | Coronary heart disease | Unspecified style, 50 minutes per time, 5 times per week. | Laboratory tests; QOL |
| Qian GY 2019 | CCS | Chinese Mainland | COPD | The style of State General Administration of Sport of Chinese Mainland in 2003, 30 minutes per time, 4 times per week, for 36 weeks. | Physical performance: pulmonary function; QOL: WHOQOL-BREF; Psychological: anxiety, depression |
| Jiao RY 2013 | CCS | Chinese Mainland | Health promotion | The style of State General Administration of Sport of Chinese Mainland in 2003, supervised by qualified instructor, 45 minutes per session, twice per day, 5 times per week, for 24 weeks. | Laboratory tests |
| Lv Y 2015 | CCS | Chinese Mainland | COPD | Unspecified style, supervised by qualified instructor, 20 minutes per time, 7 times per week. | Physical performance: pulmonary function |
| Wei LL 2019 | CCS | Chinese Mainland | Health promotion | The style of State General Administration of Sport of Chinese Mainland in 2003, 13 minutes per time, 5 times per week. | Laboratory tests |
| Liao Y 2013 | CCS | Chinese Mainland | Health promotion | Unspecified style, 30 minutes per time, 7 times per week, for 6 weeks. | QOL: SF-36 |
| Zhong SC 2009 | CCS | Chinese Mainland | Health promotion | Unspecified style, supervised by qualified instructor, 40 to 50 minutes per time, 5 times per week, for 12 weeks. | Physical performance |
| Zhang J 2016 | CCS | Chinese Mainland | Health promotion | Unspecified style, 50 to 60 minutes per time, 5 times per week, for 12 weeks. | Physical performance: balance |
| Qiu WM 2014 | CCS | Chinese Mainland | Health promotion | The style of State General Administration of Sport of Chinese Mainland in 2003, 45 minutes per session, twice per day, 5 times per week, for 24 weeks. | Laboratory tests |
| Song JC 2012 | CCS | Chinese Mainland | Health promotion | Unspecified style, 30 minutes per time, 4 times per week, for 12 weeks. | Physical performance |
| Wang Q 2013 | CCS | Chinese Mainland | Hypertension | Other style; supervised by qualified instructor, 20 to 30 minutes per time, 4 to 5 times per week, for 48 weeks. | Physical performance: blood pressure |
| Ya TQ 2015 | CCS | Chinese Mainland | Low back pain | Unspecified style, 30 minutes per time, 5 times per week, for 2 weeks. | Symptom: VAS |
| Du MS 2019 | CCS | Chinese Mainland | Schizophrenia | Unspecified style, supervised by qualified instructor, 90 minutes per time, 5 times per week, for 12 weeks. | Symptom |
| Yang H 2019 | CCS | Chinese Mainland | Type 2 diabetes | Unspecified style, supervised by qualified instructor, 20 minutes per time, 5 times per week, for 12 weeks. | Laboratory tests: FPG, HbA1c; Psychological: anxiety, depression |
| Liao Y 2011 | CCS | Chinese Mainland | Health promotion | Unspecified style, 30 minutes per time, 5 times per week, for 6 weeks. | QOL: SF-36 |
| Wang ST 2007 | CCS | Chinese Mainland | Health promotion | Unspecified style, 60 minutes per time, 5 to 7 times per week, for 24 weeks. | Physical performance |
| Jia JW 2014 | CCS | Chinese Mainland | Myopia | Homemade style, 5 times per week, for 16 weeks. | Physical performance |
| Liu WH 2018 | CCS | Chinese Mainland | Low back pain | The style of State General Administration of Sport of Chinese Mainland in 2003, supervised by qualified instructor, 30 minutes per time, 3 times per week, for 3 weeks. | Symptom: VAS, JOA; Physical performance |
| Cao B 2017 | CCS | Chinese Mainland | Health promotion | Unspecified style, 30 minutes per time, 3 times per week, for 144 weeks. | Physical performance |
| Wei GZ 2019 | CCS | Chinese Mainland | Health promotion | Unspecified style, supervised by qualified instructor, 20 to 30 minutes per session, twice per day, 5 times per week. | Symptom: VAS; QOL |
| Liu B 2015 | CCS | Chinese Mainland | Health promotion | Unspecified style, 30 minutes per time, 5 times per week, for 6 weeks. | Physical performance; QOL: WHOQOL-BREF |
| Zhang H 2014 | CS | Macao | Premenstrual syndrome | The style of State General Administration of Sport of Chinese Mainland in 2003, 30 minutes per session, twice per day, 5 times per week, for 12 weeks.. | Symptom |
| An T 2019 | CS | Chinese Mainland | Type 2 diabetes | Unspecified style, 30 minutes per time, 5 times per week, for 12 weeks. | Laboratory tests: FPG, HbA1c; Psychological: depression |
| An BC 2013 | CS | Chinese Mainland | Knee osteoarthritis | The style of Chinese Health Qigong Association, supervised by qualified instructor, 30 minutes per time, 5 times per week, for 48 weeks. | Symptom; Physical performance: strength; QOL: SF-36 |
| Chen MD 2016 | CS | Taiwan | Severe mental illness | Unspecified style, supervised by qualified instructor, 30 minutes per time, 3 times per week, for 8 weeks. | Physical performance; Psychological; QOL: SF-36 |
| Xiao B 2016 | CS | Chinese Mainland | Obesity | Unspecified style, for 12 weeks. | Laboratory tests: blood lipid; Physical performance |
| Zhang XX 2019 | CS | Chinese Mainland | PCI | Unspecified style, supervised by qualified instructor, 40 minutes per session, twice per day, 5 times per week, for 1 week. | Symptom |
| Liu RH 2013 | CS | Chinese Mainland | Stroke | Unspecified style, 30 minutes per time, 5 times per week, for 2 weeks. | Incidence: constipation; Symptom |
| Yang JL 2015 | CS | Chinese Mainland | Type 2 diabetes | The style of Chinese Traditional Sport Health Preservation, supervised by qualified instructor, 35 to 40 minutes per time, 5 to 7 times per week, for 24 weeks. | Laboratory tests: bilirubin |
| Zhou XX 2010 | CS | Chinese Mainland | Health promotion | Unspecified style, 45 minutes per time, 4 times per week, for 15 weeks. | Physical performance |
| Wu SF 2016 | CS | Chinese Mainland | Health promotion | Unspecified style, 60 minutes per time, 7 times per week, for 12 weeks. | Laboratory tests: sex hormones; Physical performance: blood pressure, strength |
| Cheng L 2017 | CS | Chinese Mainland | Perimenopausal syndrome | The style of State General Administration of Sport of Chinese Mainland in 2003, supervised by qualified instructor, 30 minutes per session, twice per day, 5 times per week, for 48 weeks. | Physical performance: BMI |
| Yang JL 2014 | CS | Chinese Mainland | Type 2 diabetes | The style of Chinese Traditional Sport Health Preservation, supervised by qualified instructor, 35 to 40 minutes per time, 5 to 7 times per week, for 24 weeks. | Laboratory tests: FPG, HbA1c, blood lipid |
| Tao QY 2015 | CS | Chinese Mainland | Type 2 diabetes | The style of Chinese Traditional Sport Health Preservation, supervised by qualified instructor, 35 to 40 minutes per time, 5 to 7 times per week, for 24 weeks. | Laboratory tests: FPG, HbA1c, blood lipid; Physical performance: BMI |
| Niu P 2012 | CS | Chinese Mainland | Type 2 diabetes | Unspecified style, supervised by qualified instructor, 30 minutes per time, 5 times per week, for 24 weeks. | Laboratory tests: FPG, HbA1c |
| Wu XY 2014 | CS | Chinese Mainland | Coronary heart disease | The style of State General Administration of Sport of Chinese Mainland in 2003, supervised by qualified instructor, 30 minutes per time, 3 times per week, for 2 weeks. | Psychological: depression |
| Peng ZH 2014 | CS | Chinese Mainland | Health promotion | Unspecified style, supervised by qualified instructor, 45 to 60 minutes per time, 4 times per week, for 16 weeks. | Psychological |
| Yu JL 2018 | CS | Chinese Mainland | Health promotion | Unspecified style, 30 minutes per time, 5 times per week, for 16 weeks. | Symptom; Physical performance: flexibility |
| Hu BJ 2008 | CS | Chinese Mainland | Health promotion | Unspecified style, supervised by qualified instructor, 90 minutes per time, 3 times per week, for 20 weeks. | Physical performance; Psychological |
| Yang HJ 2011 | CS | Chinese Mainland | Health promotion | Unspecified style, 30 minutes per time, 3 times per week, for 20 weeks. | Physical performance: cardiopulmonary function |
| Liu HF 2007 | CS | Chinese Mainland | Health promotion | Unspecified style, supervised by qualified instructor, 90 minutes per time, 5 times per week, for 12 weeks. | Psychological: SCL-90 |
| Liu W 2017 | CS | Chinese Mainland | Health promotion | Unspecified style, 45 minutes per time, 3 times per week, for 18 weeks. | Physical performance; Psychological |
| Guan TY 2013 | CS | Chinese Mainland | Health promotion | Unspecified style, supervised by qualified instructor, 45 minutes per time, 4 times per week, for 18 weeks. | Laboratory tests; Physical performance; Psychological: anxiety |
| Li HB 2018 | CS | Chinese Mainland | Fatty liver | Homemade style, supervised by qualified instructor, 40 to 60 minutes per time, 3 times per week, for 24 weeks. | Laboratory tests: liver function, blood lipid |
| Wang L 2007 | CS | Chinese Mainland | Health promotion | Unspecified style, 60 minutes per time, 4 times per week, for 24 weeks. | Physical performance |
| Pang XM 2016 | CS | Chinese Mainland | Obesity | Unspecified style, 40 to 60 minutes per time, 3 to 4 times per week, for 17 weeks. | Physical performance: BMI |
| Wang YQ 2018 | CS | Chinese Mainland | Health promotion | Unspecified style, supervised by qualified instructor, 5 times per week, for 48 weeks. | Physical performance: cardiopulmonary function |
| Xue ZH 2012 | CS | Chinese Mainland | Health promotion | Unspecified style, supervised by qualified instructor, 60 minutes per time, 5 times per week, for 18 weeks. | Physical performance |
| Xu YL 2014 | CS | Chinese Mainland | Health promotion | Unspecified style, supervised by qualified instructor, 30 minutes per time, 3 times per week, for 20 weeks. | Physical performance |
| Ling K 2011 | CS | Chinese Mainland | Hypertension | Unspecified style, 60 minutes per time, 6 times per week, for 12 weeks. | Physical performance: blood pressure |
| Huang LY 2016 | CS | Chinese Mainland | Insomnia | Unspecified style, 60 minutes per time, 4 times per week, for 32 weeks. | Laboratory tests: melatonin salt concentration in urine; Symptom: PSQI |
| Huang LY 2015 | CS | Chinese Mainland | Insomnia | Unspecified style, 60 minutes per time, 4 times per week, for 32 weeks. | Symptom: PSQI; Physical performance: balance; Psychological: anxiety |
| Peng XL 2016 | CS | Chinese Mainland | AIDS | Unspecified style, supervised by qualified instructor, 30 minutes per time, 5 times per week, for 20 weeks. | Psychological: anxiety, depression |
| Jin YY 2011 | CS | Chinese Mainland | Neck pain | Unspecified style, 5 times per week, for 6 weeks. | Symptom |
| Li QP 2016 | CS | Chinese Mainland | Health promotion | The style of State General Administration of Sport of Chinese Mainland in 2003, supervised by qualified instructor, 90 minutes per time, 5 times per week, for 4 weeks. | Laboratory tests: free radical metabolism; Physical performance: BMI |
| Xiao Y 2013 | CS | Chinese Mainland | Health promotion | Unspecified style, 30 to 50 minutes per time, 5 times per week, for 16 weeks. | Physical performance: BMI, pulmonary function |
| An BC 2015 | CS | Chinese Mainland | Sarcopenia | Unspecified style, supervised by qualified instructor, 30 minutes per time, 5 times per week, for 48 weeks. | Physical performance: BMI, strength |
| Zhou HL 2011 | CS | Chinese Mainland | Perimenopausal syndrome | The style of State General Administration of Sport of Chinese Mainland in 2003, 5 times per week, for 24 weeks. | Symptom; Psychological: depression |
| Liu XY 2016 | CS | Chinese Mainland | Falls Prevention | Unspecified style, supervised by qualified instructor, 30 to 40 minutes per time, 5 times per week, for 12 weeks. | Physical performance: balance; Psychological: fear of falling |
| Niu P 2011 | CS | Chinese Mainland | Type 2 diabetes | The style of State General Administration of Sport of Chinese Mainland in 2003, supervised by qualified instructor, 40 minutes per time, 5 times per week, for 24 weeks. | Laboratory tests: FPG, HbA1c, blood lipid; Physical performance: BMI |
| Pan HS 2008 | CS | Chinese Mainland | Health promotion | Unspecified style, supervised by qualified instructor, 30 minutes per time, 5 times per week, for 48 weeks. | Physical performance: cardiopulmonary function |
| Liu XY 2016 | CS | Chinese Mainland | Falls Prevention | Unspecified style, supervised by qualified instructor, 30 to 40 minutes per time, 5 times per week, for 12 weeks. | Physical performance: balance; Psychological: fear of falling |
| Tang BQ 2019 | CS | Chinese Mainland | Bronchial asthma | The style of State General Administration of Sport of Chinese Mainland in 2003, supervised by qualified instructor, 20 minutes per session, twice per day, 5 times per week, for 8 weeks. | Symptom; Physical performance: pulmonary function; QOL |
| Lu CL 2015 | CS | Chinese Mainland | Health promotion | Unspecified style, supervised by qualified instructor, 50 minutes per time, 4 times per week, for 16 weeks. | Physical performance |
| Zhang XT 2016 | CS | Chinese Mainland | Health promotion | Unspecified style, 20 to 30 minutes per time, 6 times per week | Symptom |
| Yang RB 2014 | CS | Chinese Mainland | Hypertension | Unspecified style, 7 times per week, for 48 weeks. | Symptom; Physical performance: blood pressure |
| Guo Y 2019 | CS | Chinese Mainland | Health promotion | The style of State General Administration of Sport of Chinese Mainland in 2003, supervised by qualified instructor, 15 minutes per time, 5 times per week, for 10 weeks. | Laboratory tests: electroencephalogram |
| Guo Y 2018 | CS | Chinese Mainland | Health promotion | The style of State General Administration of Sport of Chinese Mainland in 2003, supervised by qualified instructor, 15 minutes per time, 5 times per week, for 10 weeks. | Laboratory tests: electroencephalogram |
| Liu Y 2016 | CS | Chinese Mainland | Health promotion | The style of State General Administration of Sport of Chinese Mainland in 2003, 90 minutes per time, 5 times per week, for 18 weeks. | Physical performance: BMI, pulmonary function; |
| Wen SZ 2016 | CS | Chinese Mainland | Health promotion | The style of State General Administration of Sport of Chinese Mainland in 2003, supervised by qualified instructor, 90 minutes per time, 5 times per week, for 16 weeks. | Laboratory tests: blood lipid; Physical performance: blood pressure, pulmonary function |
| Gao W 2018 | CS | Chinese Mainland | Health promotion | The style of State General Administration of Sport of Chinese Mainland in 2003, 90 minutes per time, twice per week, for 16 weeks. | Psychological: SCL-90 |
| Hu WW 2017 | CS | Chinese Mainland | Health promotion | Unspecified style, 7 times per week, for 14 weeks. | Psychological: SCL-90 |
| Su YH 2018 | CS | Chinese Mainland | Health promotion | The style of State General Administration of Sport of Chinese Mainland in 2003, supervised by qualified instructor, 40 minutes per time, 5 times per week, for 8 weeks. | Laboratory tests: BMD, strength, balance, pulmonary function |
| Zeng YH 2005 | CS | Chinese Mainland | Health promotion | Unspecified style, 60 minutes per time, 5 times per week, for 10 weeks. | Physical performance |
| Huang T 2005 | CS | Chinese Mainland | Health promotion | Unspecified style, supervised by qualified instructor, 50 minutes per time, 5 times per week, for 10 weeks. | Laboratory tests |
| Lun HD 2018 | CS | Chinese Mainland | Health promotion | Unspecified style, 90 minutes per time, 6 times per week, for 24 weeks. | Psychological: SCL-90 |
| Gao W 2018 | CS | Chinese Mainland | Health promotion | Unspecified style, 90 minutes per time, twice per week, for 16 weeks. | Psychological: SCL-90 |
| Yang Y 2017 | CS | Chinese Mainland | Health promotion | Unspecified style, 90 minutes per time, 4 times per week, for 12 weeks. | Physical performance: heart rate |
| Wan PF 2018 | CS | Chinese Mainland | Health promotion | Unspecified style, 90 minutes per time, 6 times per week, for 24 weeks. | QOL: SF-36 |
| Cheng GH 2016 | CS | Chinese Mainland | Health promotion | Unspecified style, 45 to 60 minutes per time, 2 to 3 times per week, for 24 weeks. | Physical performance |
| Cui YS 2018 | CS | Chinese Mainland | Health promotion | Unspecified style, 60 minutes per time, 6 times per week, for 24 weeks. | Psychological: SCL-90 |
| Shao CS 2018 | CS | Chinese Mainland | Health promotion | Unspecified style, 60 minutes per time, 5 times per week, for 8 weeks. | QOL: WHOQOL-BREF |
| Zhang C 2014 | CS | Chinese Mainland | Health promotion | The style of State General Administration of Sport of Chinese Mainland in 2003, supervised by qualified instructor, 60 minutes per time, 3 times per week, for 24 weeks. | Laboratory tests: blood lipid; Physical performance: blood pressure |
| Zhu NN 2016 | CS | Chinese Mainland | Health promotion | The style of State General Administration of Sport of Chinese Mainland in 2003, supervised by qualified instructor, 50 to 60 minutes per time, 3 times per week, for 24 weeks. | Laboratory tests: blood lipid; Physical performance |
| Zhou XQ 2003 | CS | Chinese Mainland | Health promotion | Unspecified style, 60 minutes per time, 5 times per week, for 10 weeks. | Physical performance |
| Zhou XQ 2007 | CS | Chinese Mainland | Health promotion | Unspecified style, supervised by qualified instructor, 60 minutes per time, 5 times per week, for 10 weeks. | Laboratory tests: blood lipid |
| Huang T 2003 | CS | Chinese Mainland | Health promotion | Unspecified style, supervised by qualified instructor, 50 minutes per time, 5 times per week, for 10 weeks. | Laboratory tests: free radical metabolism, sex hormones |
| Li MJ 2015 | CS | Chinese Mainland | Health promotion | Unspecified style, supervised by qualified instructor, 60 minutes per time, 3 times per week, for 12 weeks. | QOL: SF-36; Psychological: SCL-90 |
| Sun HM 2012 | CS | Chinese Mainland | Health promotion | Unspecified style, supervised by qualified instructor, 40 to 50 minutes per time, 6 times per week, for 24 weeks. | Laboratory tests: gut microbiota |
| Jin XZ 2017 | CS | Chinese Mainland | Health promotion | The style of State General Administration of Sport of Chinese Mainland in 2003, supervised by qualified instructor, 40 minutes per time, 5 times per week, for 8 weeks. | Physical performance |
| Zhang SC 2018 | CS | Chinese Mainland | Health promotion | The style of State General Administration of Sport of Chinese Mainland in 2003, 40 minutes per time, 5 times per week, for 8 weeks. | Laboratory tests: BMD; Physical performance: strength, balance |
| Yang YH 2018 | CS | Chinese Mainland | Knee osteoarthritis | The style of State General Administration of Sport of Chinese Mainland in 2003, supervised by qualified instructor, 90 minutes per time, 3 times per week, for 12 weeks. | Symptom |
| Zhang J 2016 | CS | Chinese Mainland | Health promotion | The style of State General Administration of Sport of Chinese Mainland in 2003, supervised by qualified instructor, 60 minutes per time, 3 times per week, for 24 weeks. | Laboratory tests: blood lipid, gut microbiota |
| Leng QH 2016 | CS | Chinese Mainland | Premenstrual syndrome | The style of State General Administration of Sport of Chinese Mainland in 2003, 30 minutes per time, 5 times per week, for 12 weeks. | Symptom |
| Lin JF 2018 | CS | Chinese Mainland | Health promotion | The style of State General Administration of Sport of Chinese Mainland in 2003, 45 minutes per time, once per week, for 18 weeks. | Physical performance |
| Gu KP 2014 | CS | Chinese Mainland | Hyperlipidemia | The style of Beijing Sport University, supervised by qualified instructor, 60 minutes per time, 5 to 7 times per week, for 24 weeks. | Laboratory tests: blood lipid |
| Chen WR 2015 | CS | Chinese Mainland | Hyperlipidemia | Unspecified style, 40 minutes per time, 5 times per week, for 12 weeks. | Laboratory tests: blood lipid; QOL: SF-36 |
| Wang H 2014 | CS | Chinese Mainland | Health promotion | Unspecified style, supervised by qualified instructor, 30 minutes per time, 5 times per week, for 16 weeks. | Laboratory tests: immunity; Symptom; Psychological: anxiety |
| Chen PP 2016 | CS | Chinese Mainland | Health promotion | Unspecified style, 60 minutes per time, 5 times per week, for 32 weeks. | Laboratory tests: melatonin salt concentration in urine; Symptom: PSQI; Physical performance: balance; Psychological: anxiety |
| Wang XX 2003 | CS | Chinese Mainland | Health promotion | Unspecified style, 60 minutes per time, 5 times per week, for 10 weeks. | Laboratory tests: immunity |
| Wang AL 2005 | CS | Chinese Mainland | Health promotion | Unspecified style, 60 minutes per time, 5 to 6 times per week, for 10 weeks. | Laboratory tests: blood lipid, immunity; Physical performance; Psychological: SCL-90 |
| Guo Y 2019 | CS | Chinese Mainland | Health promotion | The style of Beijing Sport University, supervised by qualified instructor, 15minutes per time, 5 times per week, for 10 weeks. | Laboratory tests: electroencephalogram |
| Cheng LJ 2015 | CS | Chinese Mainland | Health promotion | Unspecified style, supervised by qualified instructor, 90 minutes per time, 5 times per week, for 4 weeks. | Symptom: NDI |
| Kong L 2008 | CS | Chinese Mainland | osteoarthritis | Unspecified style, supervised by qualified instructor, 30 to 45 minutes per time, 6 times per week, for 48 weeks. | Physical performance: blood pressure, flexibility |
| Yu T 2018 | CS | Chinese Mainland | Neurasthenia | Unspecified style, supervised by qualified instructor, 20 to 50 minutes per time, 3 to 5 times per week, for 20 weeks. | Symptom |
| Xie Y 2019 | RCT | Chinese Mainland | Ankylosing spondylitis | Unspecified style, supervised by qualified instructor, 2 to 3 times per week, for 12 weeks. | Symptom |
| Jessie SM 2017 | RCT | Hong Kong | Chronic fatigue syndrome | Unspecified style, supervised by qualified instructor, 30 minutes per time, twice per week, for 9 weeks. | Laboratory tests: measurement of plasma adiponectin levels; Psychological: HADS |
| An B 2008 | RCT | Chinese Mainland | osteoarthritis | Unspecified style, supervised by qualified instructor, 30 minutes per time, 5 times per week, for 8 weeks. | Symptom; Physical performance: strength; QOL: SF-36 |
| Yu M 2018 | RCT | Chinese Mainland | Ischemic heart failure | The style of State General Administration of Sport of Chinese Mainland in 2003, supervised by qualified instructor, 45 minutes per time, twice per week, for 12 weeks. | Laboratory tests; Symptom; Physical performance; QOL |
| Zheng G 2019 | RCT | Chinese Mainland | Stroke | The style of State General Administration of Sport of Chinese Mainland in 2003, supervised by qualified instructor, 60 minutes per time, 5 times per week, for 12 weeks. | Laboratory tests: FPG, blood lipid; Symptom: PSQI; Physical performance: BMI, cardiopulmonary function; QOL: SF-36; Psychological |
| Wang JY 2014 | RCT | Chinese Mainland | Neck pain | The style of State General Administration of Sport of Chinese Mainland in 2003, supervised by qualified instructor, 30 minutes per time, 5 times per week, for 24 weeks. | Symptom: VAS; QOL: SF-36 |
| Wang F 2008 | RCT | Chinese Mainland | Type 2 diabetes | Unspecified style, 60 minutes per time, 5 times per week, for 16 weeks. | Laboratory tests: blood glucose, blood lipid; Psychological: SCL-90 |
| Liu J 2019 | RCT | Chinese Mainland | Health promotion | The style of State General Administration of Sport of Chinese Mainland in 2003, supervised by qualified instructor, 60 minutes per time, 5 times per week, for 12 weeks. | Symptom; Physical performance |
| Chen MC 2012 | RCT | Taiwan | Insomnia | Homemade style, supervised by qualified instructor, 30 minutes per time, 3 times per week, for 12 weeks. | Symptom: PSQI; Psychological |
| Hsu CH 2015 | RCT | Taiwan | Non-Hodgkin lymphoma | Unspecified style, for 12 weeks. | QOL |
| Li R 2014 | RCT | Chinese Mainland | Health promotion | The style of State General Administration of Sport of Chinese Mainland in 2003, supervised by qualified instructor, 30 to 60 minutes per time, 3 times per week, for 16 weeks. | Laboratory tests: blood glucose, blood lipid; Physical performance: BMI, cardiopulmonary function; |
| Jing L 2018 | RCT | Chinese Mainland | Health promotion | Unspecified style, supervised by qualified instructor, for 24 weeks. | Physical performance: cardiopulmonary function; QOL; Psychological |
| Lu Y 2019 | RCT | Chinese Mainland | Colorectal cancer | Other style, supervised by qualified instructor, 20 to 40 minutes per time, 5 times per week, for 24 weeks. | Symptom |
| Li M 2015 | RCT | Chinese Mainland | Health promotion | The style of State General Administration of Sport of Chinese Mainland in 2003, supervised by qualified instructor, 60 minutes per time, 5 times per week, for 12 weeks. | Physical performance: strength, cardiopulmonary function; Psychological |
| Xiao X 2018 | RCT | Chinese Mainland | Cardiovascular diseases | The style of State General Administration of Sport of Chinese Mainland in 2003, supervised by qualified instructor, 24 minutes per time, 5 times per week, for 16 weeks. | Symptom |
| Xiao CM 2016 | RCT | Chinese Mainland | Parkinson's disease | The style of Chinese Health Qigong Association, supervised by qualified instructor, 45 minutes per time, 4 times per week, for 24 weeks. | Symptom; Physical performance: balance, cognition, motion |
| Xiao C 2016 | RCT | Chinese Mainland | Hypertension | The style of Chinese Health Qigong Association, 40 minutes per time, 5 times per week, for 24 weeks. | Laboratory tests: blood glucose, blood lipid; Physical performance: blood pressure |
| Liu BX 2015 | RCT | Chinese Mainland | Osteoporosis | Homemade style, for 48 weeks. | Symptom; Laboratory tests: BMD |
| Tian Q 2013 | RCT | Chinese Mainland | Primary fibromyalgia syndrome | Unspecified style, 30 minutes per time, 3 times per week, for 8 weeks. | Symptom: VAS |
| Chen DM 2018 | RCT | Taiwan | Heart failure | The style of Chinese Traditional Sport Health Preservation, supervised by qualified instructor, 35 minutes per time, 3 times per week, for 12 weeks. | Symptom; Physical performance |
| Chen HH 2006 | RCT | Taiwan | Osteopenia | Unspecified style, 3 times per week, for 12 weeks. | Laboratory tests: BMD |
| Duan F 2018 | RCT | Chinese Mainland | Depression | The style of State General Administration of Sport of Chinese Mainland in 2003, supervised by qualified instructor, 60 minutes per time, 4 times per week, for 12 weeks. | Psychological |
| Liu XY 2016 | RCT | Chinese Mainland | Falls Prevention | The style of State General Administration of Sport of Chinese Mainland in 2003, supervised by qualified instructor, 30 to 40 minutes per time, 5 times per week, for 12 weeks. | Physical performance: balance |
| Lv W 2019 | RCT | Chinese Mainland | Health promotion | Unspecified style, 60 minutes per time, 5 times per week, for 12 weeks. | Symptom: PSQI; Physical performance: cognition, cardiopulmonary function |
| Ying W 2019 | RCT | Chinese Mainland | Breast cancer | The style of State General Administration of Sport of Chinese Mainland in 2003, supervised by qualified instructor, 60 minutes per time, 5 times per week, for 24 weeks. | Symptom; Physical performance: BMI; QOL; Psychological |
| Tao J 2016 | RCT | Chinese Mainland | Health promotion | The style of State General Administration of Sport of Chinese Mainland in 2003, supervised by qualified instructor, 60 minutes per time, 5 times per week, for 12 weeks. | Physical performance: memory function |
| Zhu NJ 2015 | RCT | Chinese Mainland | Type 2 diabetes | Unspecified style, for 48 weeks. | Symptom: MoCA; QOL |
| Cheung DST 2019 | RCT | Hong Kong | Mental illness | The style of Chinese Health Qigong Association, supervised by qualified instructor, 30 minutes per time, 5 times per week, for 22 weeks. | Laboratory tests: telomerase flexibility; Psychological: depression |
| Tao J 2019 | RCT | Chinese Mainland | Cognitive impairment | The style of State General Administration of Sport of Chinese Mainland in 2003, supervised by qualified instructor, 60 minutes per time, 3 times per week, for 24 weeks. | Symptom: MoCA |
| Liu J 2019 | RCT | Chinese Mainland | Knee osteoarthritis | Unspecified style, supervised by qualified instructor, 60 minutes per time, 5 times per week, for 12 weeks. | Laboratory tests; Symptom |
| Chan JS 2014 | RCT | Hong Kong | Chronic fatigue syndrome | The style of State General Administration of Sport of Chinese Mainland in 2003, supervised by qualified instructor, 90 minutes per time, 2 times per week, for 12 weeks. | Symptom: PSQI; Psychological: anxiety, depression |
| Manzaneque JM 2009 | RCT | Spain | Mental illness | Unspecified style, supervised by qualified instructor, 30 minutes per time, 3 times per week, for 4 weeks. | Laboratory tests; Symptom: PSQI; Psychological: anxiety, depression |
| Tsang HW 2003 | RCT | Hong Kong | Mental illness | Unspecified style, supervised by qualified instructor, 30 minutes per time, 2 times per week, for 12 weeks. | QOL: WHOQOL-BREF; Psychological: depression |
| Tsang HW 2006 | RCT | Hong Kong | Depression | Unspecified style, supervised by qualified instructor, 30 to 45 minutes per time, 3 times per week, for 16 weeks. | Psychological: depression |
| Tsang HW 2013 | RCT | Hong Kong | Depression | Unspecified style, supervised by qualified instructor, 45 minutes per time, 3 times per week, for 12 weeks. | Laboratory tests; Physical performance: strength, flexibility; Psychological: depression |
| Liu XD 2012 | RCT | Chinese Mainland | COPD | The style of State General Administration of Sport of Chinese Mainland in 2003, supervised by qualified instructor, 60 minutes per time, 3 times per week, for 24 weeks. | Laboratory tests; Physical performance: pulmonary function; QOL |
| Li ZM 2019 | RCT | Chinese Mainland | Chronic kidney disease | The style of State General Administration of Sport of Chinese Mainland in 2003, supervised by qualified instructor, 50 minutes per time, 4 times per week, for 24 weeks. | Laboratory tests |
| Wang HO 2013 | RCT | Chinese Mainland | Health promotion | Unspecified style, 45 minutes per time, 4 times per week, for 12 weeks. | Physical performance: heart rate, blood pressure, pulmonary function |
| Tao J 2017 | RCT | Chinese Mainland | Cognitive impairment | The style of State General Administration of Sport of Chinese Mainland in 2003, supervised by qualified instructor, 60 minutes per time, 5 times per week, for 12 weeks. | Physical performance: memory function |
| Jin L 2017 | RCT | Chinese Mainland | Health promotion | Unspecified style, supervised by qualified instructor, 30 to 60 minutes per time, 3 times per week, for 16 weeks. | Laboratory tests: surface electromyography |
| Tao J 2017 | RCT | Chinese Mainland | Health promotion | The style of State General Administration of Sport of Chinese Mainland in 2003, supervised by qualified instructor, 60 minutes per time, 5 times per week, for 12 weeks. | Symptom; Laboratory tests |
| Tao J 2017 | RCT | Chinese Mainland | Cognitive impairment | The style of State General Administration of Sport of Chinese Mainland in 2003, supervised by qualified instructor, 60 minutes per time, 5 times per week, for 12 weeks. | Symptom; Laboratory tests |
| Jiao J 2019 | RCT | Chinese Mainland | Fibromyalgia | Unspecified style, supervised by qualified instructor, 60 minutes per time, 2 times per week, for 12 weeks. | Symptom: VAS, PSQI; QOL; Psychological: depression |
| Liu J 2019 | RCT | Chinese Mainland | Knee osteoarthritis | The style of State General Administration of Sport of Chinese Mainland in 2003, supervised by qualified instructor, 60 minutes per time, 5 times per week, for 12 weeks. | Symptom; Laboratory tests; Psychological: depression |
| Xia R 2019 | RCT | Chinese Mainland | Cognitive impairment | The style of State General Administration of Sport of Chinese Mainland in 2003, supervised by qualified instructor, 60 minutes per time, 3 times per week, for 24 weeks. | Laboratory tests; Physical performance: attentional ability |
| Liu Y 2011 | RCT | Chinese Mainland | Type 2 diabetes | The style of State General Administration of Sport of Chinese Mainland in 2003, supervised by qualified instructor, 45 minutes per time, 3 to 5 times per week, for 12 weeks. | Laboratory tests: HbA1c; QOL; Psychological: depression |
| Wang C 2010 | RCT | Chinese Mainland | Frozen shoulder | The style of State General Administration of Sport of Chinese Mainland in 2003, 30 minutes per session, twice per day, 3 to 5 times per week, for 4 weeks. | Symptom: VAS |
| Li B 2016 | RCT | Chinese Mainland | Breast cancer | Unspecified style, 10 to 15 minutes per time, 5 times per week, for 24 weeks. | Laboratory tests |
| Lin Q 2018 | RCT | Chinese Mainland | Stroke | Unspecified style, 30 minutes per time, 5 times per week, for 24 weeks. | Symptom; QOL; Psychological: anxiety |
| Luo XL 2019 | RCT | Chinese Mainland | Type 2 diabetes | Unspecified style, 30 minutes per time, 5 times per week, for 72 weeks. | Laboratory tests: FPG, HbA1c |
| Luo XL 2019 | RCT | Chinese Mainland | Type 2 diabetes | Unspecified style, 30 minutes per time, 5 times per week, for 72 weeks. | Laboratory tests: blood lipid; Physical performance: heart rate, blood pressure, BMI |
| Qiu P 2017 | RCT | Chinese Mainland | Cancer | Unspecified style, supervised by qualified instructor, 30 minutes per session, twice per day, 5 times per week, for 8 weeks. | Symptom; QOL |
| Jin L 2015 | RCT | Chinese Mainland | Health promotion | Unspecified style, 11 minutes per time, for 1 weeks. | Physical performance |
| Zhu XN 2015 | RCT | Chinese Mainland | Health promotion | Unspecified style, supervised by qualified instructor, 30 to 45 minutes per time, 3 times per week, for 16 weeks. | Psychological: depression |
| Zhang LX 2017 | RCT | Chinese Mainland | COPD | Unspecified style, 5 times per week, for 8 weeks. | Physical performance: BMI, pulmonary function |
| Shi BX 2017 | RCT | Chinese Mainland | Neck pain | Unspecified style, supervised by qualified instructor, 60 minutes per session, twice per day, 5 times per week, for 12 weeks. | Symptom |
| Zhu LG 2005 | RCT | Chinese Mainland | Hyperlipidemia | Unspecified style, 20 minutes per session, twice per day, 5 times per week, for 12 weeks. | Laboratory tests: blood lipid |
| Liu Q 2014 | RCT | Chinese Mainland | Chronic diseases | Unspecified style, supervised by qualified instructor, 30 minutes per time, 5 times per week, for 24 weeks. | QOL |
| Deng XF 2019 | RCT | Chinese Mainland | Heart failure | Unspecified style, supervised by qualified instructor, 10 minutes per time, 5 times per week, for 24 weeks. | Symptom; Laboratory tests; Physical performance: cardiopulmonary function; QOL |
| Tian H 2017 | RCT | Chinese Mainland | Stroke | Unspecified style, supervised by qualified instructor, 30 to 40 minutes per time, 5 times per week, for 12 weeks. | Symptom: balance |
| Lu LX 2019 | RCT | Chinese Mainland | Dysmenorrhea | The style of State General Administration of Sport of Chinese Mainland in 2003, 30 minutes per time, 5 times per week, for 12 weeks. | Symptom: VAS; Laboratory tests |
| Hu SX 2019 | RCT | Chinese Mainland | Neck pain | Unspecified style, 20 to 30 minutes per time, 5 times per week, for 12 weeks. | Symptom: VAS; Physical performance: range of motion |
| Liang LX 2019 | RCT | Chinese Mainland | Lumbar fracture | Unspecified style, supervised by qualified instructor, 15 minutes per session, twice per day, 5 times per week, for 4 weeks. | Symptom: VAS; QOL |
| Wang ZG 2016 | RCT | Chinese Mainland | Low back pain | Unspecified style, 15 minutes per session, twice per day, 5 times per week, for 4 weeks. | Symptom: VAS, JOA |
| Chen XY 2013 | RCT | Chinese Mainland | Low back pain | Unspecified style, supervised by qualified instructor, 15 minutes per session, twice per day, 5 times per week, for 4 weeks. | Symptom: JOA |
| Yu XL 2017 | RCT | Chinese Mainland | Coronary heart disease | Unspecified style, 30 to 50 minutes per time, 5 times per week, for 4 weeks. | Symptom: PSQI |
| Cai JD 2015 | RCT | Chinese Mainland | Neck pain | Unspecified style, supervised by qualified instructor, 30 minutes per session, twice per day, 5 times per week, for 24 weeks. | Symptom: VAS |
| Liu YM 2017 | RCT | Chinese Mainland | Neck pain | Unspecified style, 30 minutes per time, 5 times per week, for 8 weeks. | Symptom: JOA |
| Pang H 2013 | RCT | Chinese Mainland | Low back pain | The style of State General Administration of Sport of Chinese Mainland in 2003, 30 minutes per time, 5 times per week, for 8 weeks. | Symptom: VAS, JOA |
| Deng YF 2015 | RCT | Chinese Mainland | COPD | The style of State General Administration of Sport of Chinese Mainland in 2003, supervised by qualified instructor, 30 minutes per time, 5 times per week, for 12 weeks. | Symptom: pulmonary function |
| Liang XL 2016 | RCT | Chinese Mainland | COPD | Unspecified style, 30 minutes per time, 5 times per week, for 12 weeks. | Symptom: pulmonary function |
| Song CA 2019 | RCT | Chinese Mainland | Lung cancer | Unspecified style, supervised by qualified instructor, 30 minutes per time, 5 times per week, for 4 weeks. | Symptom: PSQI |
| Chen JX 2015 | RCT | Chinese Mainland | COPD | The style of State General Administration of Sport of Chinese Mainland in 2003, supervised by qualified instructor, 30 minutes per time, 5 times per week, for 12 weeks. | Symptom: pulmonary function |
| Ding Y 2019 | RCT | Chinese Mainland | Stroke | The style of State General Administration of Sport of Chinese Mainland in 2003, 20 minutes per session, twice per day, 5 times per week, for 4 weeks. | Symptom; Physical performance: balance |
| Lin Y 2017 | RCT | Chinese Mainland | Type 2 diabetes | Unspecified style, supervised by qualified instructor, 20 minutes per session, twice per day, 5 times per week, for 12 weeks. | Laboratory tests: HbA1c |
| Zhou Y 2011 | RCT | Chinese Mainland | Neck pain | Unspecified style, supervised by qualified instructor, 60 minutes per time, 5 times per week, for 24 weeks. | Physical performance |
| Yang H 2015 | RCT | Chinese Mainland | Hypertension | Unspecified style, 40 minutes per time, 5 times per week, for 24 weeks. | QOL: SF-36 |
| Yang H 2014 | RCT | Chinese Mainland | Hypertension | Homemade style, supervised by qualified instructor, 40 minutes per time, 5 times per week, for 24 weeks. | Symptom; Physical performance: heart rate, blood pressure |
| Shang YH 2017 | RCT | Chinese Mainland | Neck pain | The style of State General Administration of Sport of Chinese Mainland in 2003, supervised by qualified instructor, 30 minutes per time, 5 times per week, for 12 weeks. | Symptom; Physical performance: range of motion |
| Li HH 2019 | RCT | Chinese Mainland | Neck pain | The style of State General Administration of Sport of Chinese Mainland in 2003, supervised by qualified instructor, 60 minutes per time, 5 times per week, for 12 weeks. | Symptom: VAS; Laboratory tests: surface electromyography; Psychological: anxiety |
| Pan HS 2010 | RCT | Chinese Mainland | Hypertension | The style of State General Administration of Sport of Chinese Mainland in 2003, 45 minutes per session, twice per day, 5 times per week, for 24 weeks. | Laboratory tests: blood glucose, blood lipid; Physical performance: blood pressure |
| Du WY 2018 | RCT | Chinese Mainland | Health promotion | The style of State General Administration of Sport of Chinese Mainland in 2003, 60 minutes per time, 5 times per week, for 24 weeks. | Physical performance: balance |
| Gao DF 2012 | RCT | Chinese Mainland | Schizophrenia | The style of State General Administration of Sport of Chinese Mainland in 2003, supervised by qualified instructor, 90 minutes per time, 5 times per week, for 12 weeks. | Symptom |
| Cui YS 2019 | RCT | Chinese Mainland | Stroke | The style of State General Administration of Sport of Chinese Mainland in 2003, 45 minutes per time, 5 times per week, for 8 weeks. | Physical performance: balance |
| Zhang SY 2018 | RCT | Chinese Mainland | Health promotion | The style of State General Administration of Sport of Chinese Mainland in 2003, supervised by qualified instructor, 60 minutes per time, 5 times per week, for 12 weeks. | Symptom: PSQI; Laboratory tests: polysomnogram |
| Liu XY 2014 | RCT | Chinese Mainland | Health promotion | Unspecified style, supervised by qualified instructor, 30 to 40 minutes per time, 5 times per week, for 12 weeks. | QOL: SF-36 |
| Sun LJ 2017 | RCT | Chinese Mainland | Knee osteoarthritis | Unspecified style, supervised by qualified instructor, 20 to 30 minutes per session, twice per day, 5 times per week, for 4 weeks. | Symptom: VAS, strength, range of motion |
| He JH 2010 | RCT | Chinese Mainland | Health promotion | Unspecified style, supervised by qualified instructor, 60 minutes per time, 3 times per week, for 20 weeks. | Physical performance; Psychological: SCL-90 |
| Liu XJ 2011 | RCT | Chinese Mainland | Osteoporosis | The style of State General Administration of Sport of Chinese Mainland in 2003, supervised by qualified instructor, 60 minutes per time, 6 times per week, for 24 weeks. | Symptom; Laboratory tests: BMD |
| Yue HX 2011 | RCT | Chinese Mainland | Health promotion | Unspecified style, supervised by qualified instructor, 60 minutes per time, 5 times per week, for 24 weeks. | Laboratory tests: hemorheology |
| Li CX 2010 | RCT | Chinese Mainland | Frozen shoulder | Unspecified style, supervised by qualified instructor, 60 minutes per time, 5 times per week, for 24 weeks. | Symptom: strength, range of motion |
| Gao DF 2010 | RCT | Chinese Mainland | Schizophrenia | The style of State General Administration of Sport of Chinese Mainland in 2003, supervised by qualified instructor, 60 minutes per time, 5 times per week, for 12 weeks. | Physical performance: BMI |
| Gao DF 2010 | RCT | Chinese Mainland | Schizophrenia | The style of State General Administration of Sport of Chinese Mainland in 2003, supervised by qualified instructor, 90 minutes per time, 5 times per week, for 12 weeks. | Symptom |
| Wang C 2009 | RCT | Chinese Mainland | Frozen shoulder | The style of State General Administration of Sport of Chinese Mainland in 2003, 0 5 times per week, for 5 weeks. | Symptom: VAS, range of motion |
| Wu X 2017 | RCT | Chinese Mainland | Falls Prevention | The style of State General Administration of Sport of Chinese Mainland in 2003, supervised by qualified instructor, 45 minutes per time, 5 times per week, for 4 weeks. | Physical performance: balance; QOL: SF-36; Psychological: anxiety |
| Zhou Y 2013 | RCT | Chinese Mainland | Obesity | The style of State General Administration of Sport of Chinese Mainland in 2003, 90 minutes per time, 5 times per week, for 14 weeks. | Laboratory tests: serum leptin, adiponectin, blood lipid; Physical performance: BMI |
| Yuan JL 2019 | RCT | Chinese Mainland | Neck pain | The style of State General Administration of Sport of Chinese Mainland in 2003, 40 minutes per time, 5 times per week, for 12 weeks. | Symptom: VAS; Psychological: anxiety, depression |
| Zheng HY 2017 | RCT | Chinese Mainland | Schizophrenia | Unspecified style, 30 minutes per time, 5 times per week, for 12 weeks. | Symptom; Laboratory tests: blood lipid |
| Xu XX 2018 | RCT | Chinese Mainland | Low back pain | The style of State General Administration of Sport of Chinese Mainland in 2003, 30 minutes per time, 5 times per week, for 12 weeks. | Symptom: VAS; QOL: SF-36 |
| Wei Q 2019 | RCT | Chinese Mainland | Stroke | Unspecified style, 30 minutes per time, 5 times per week, for 12 weeks. | Symptom; QOL |
| Guan FP 2017 | RCT | Chinese Mainland | Schizophrenia | Unspecified style, supervised by qualified instructor, 60 minutes per time, 5 times per week, for 12 weeks. | Symptom |
| Li CX 2014 | RCT | Chinese Mainland | Depression | Unspecified style, supervised by qualified instructor, 60 minutes per time, 5 times per week, for 40 weeks. | Laboratory tests; Psychological: depression |
| Lin F 2014 | RCT | Chinese Mainland | Hypertension | Unspecified style, supervised by qualified instructor, 30 minutes per session, twice per day, 5 times per week, for 12 weeks. | Physical performance: blood pressure |
| Zhou Y 2012 | RCT | Chinese Mainland | Dysmenorrhea | Unspecified style, supervised by qualified instructor, 60 minutes per time, 5 times per week, for 24 weeks. | Symptom; Laboratory tests |
| Liu XH 2014 | RCT | Chinese Mainland | Hypertension | Unspecified style, 20 to 40 minutes per time, 3 to 5 times per week, for 12 weeks. | Laboratory tests; Physical performance: blood pressure |
| Wu WZ 2019 | RCT | Chinese Mainland | Type 2 diabetes | The style of State General Administration of Sport of Chinese Mainland in 2003, 30 minutes per session, twice per day, 5 times per week, for 12 weeks. | Laboratory tests: FPG, HbA1c, blood lipid |
| Xu QJ 2017 | RCT | Chinese Mainland | Type 2 diabetes | The style of State General Administration of Sport of Chinese Mainland in 2003, supervised by qualified instructor, 60 minutes per time, 5 times per week, for 12 weeks. | Symptom; Laboratory tests: HbA1c; Psychological: anxiety |
| He K 2017 | RCT | Chinese Mainland | Type 2 diabetes | Unspecified style, supervised by qualified instructor, 30 to 60 minutes per time, 5 times per week, for 48 weeks. | QOL |
| Wei QB 2014 | RCT | Chinese Mainland | Type 2 diabetes | Unspecified style, supervised by qualified instructor, 30 minutes per time, 5 times per week, for 12 weeks. | QOL: SF-36 |
| Zhang L 2005 | RCT | Chinese Mainland | Type 2 diabetes | Unspecified style, supervised by qualified instructor, 60 minutes per time, 3 times per week, for 16 weeks. | Laboratory tests: FPG, HbA1c, blood lipid; QOL; Psychological: SCL-90 |
| Li QW 2016 | RCT | Chinese Mainland | Type 2 diabetes | Unspecified style, supervised by qualified instructor, 45 to 50 minutes per time, 3 times per week, for 12 weeks. | Laboratory tests: FPG, HbA1c; Physical performance: BMI, strength |
| Guo QQ 2019 | RCT | Chinese Mainland | Type 2 diabetes | Unspecified style, supervised by qualified instructor, 40 minutes per time, 4 times per week, for 12 weeks. | Symptom; Laboratory tests: FPG, HbA1c, blood lipid; Physical performance: heart rate, blood pressure, strength, range of motion |
| Peng DZ 2015 | RCT | Chinese Mainland | Type 2 diabetes | The style of State General Administration of Sport of Chinese Mainland in 2003, supervised by qualified instructor, 30 minutes per time, 5 times per week, for 24 weeks. | Laboratory tests: HbA1c; QOL; Psychological: anxiety, SCL-90 |
| Ke XJ 2018 | RCT | Chinese Mainland | Neck pain | The style of State General Administration of Sport of Chinese Mainland in 2003, supervised by qualified instructor, 45 to 60 minutes per time, 5 times per week, for 10 weeks. | Symptom: VAS, range of motion |
| Liu AL 2012 | RCT | Chinese Mainland | Health promotion | Unspecified style, supervised by qualified instructor, 45 to 60 minutes per time, 5 times per week, for 10 weeks. | Physical performance |
| Chen TG 2013 | RCT | Chinese Mainland | Health promotion | Unspecified style, supervised by qualified instructor, 90 minutes per time, 5 times per week, for 24 weeks. | Psychological |
| Yan HJ 2017 | RCT | Chinese Mainland | Health promotion | Unspecified style, twice per day, 5 times per week, for 12 weeks. | Physical performance; Psychological: SCL-90 |
| Liang HY 2013 | RCT | Chinese Mainland | Hypertension | Unspecified style, twice per day, 5 times per week, for 12 weeks. | Physical performance: blood pressure; QOL |
| Li Y 2018 | RCT | Chinese Mainland | Polycystic Ovary Syndrome | Unspecified style, supervised by qualified instructor, 90 minutes per time, 5 times per week, for 40 weeks. | Laboratory tests; Physical performance: BMI |
| Li Y 2018 | RCT | Chinese Mainland | Polycystic Ovary Syndrome | Unspecified style, supervised by qualified instructor, 120 minutes per time, 5 times per week, for 24 weeks. | Psychological: anxiety, depression, SCL-90 |
| Wang QR 2018 | RCT | Chinese Mainland | Low back pain | The style of State General Administration of Sport of Chinese Mainland in 2003, supervised by qualified instructor, 5 minutes per time, 5 times per week, for 10 weeks. | Symptom: VAS, ODI; Psychological: anxiety |
| Cao C 2016 | RCT | Chinese Mainland | COPD | The style of State General Administration of Sport of Chinese Mainland in 2003, 30 minutes per time, 4 times per week, for 24 weeks. | Physical performance: pulmonary function; Psychological: anxiety, depression |
| Hou C 2017 | RCT | Chinese Mainland | Depression | Unspecified style, 30 minutes per time, 7 times per week, for 8 weeks. | Symptom: PSQI; Psychological: anxiety, depression |
| Gao HM 2017 | RCT | Chinese Mainland | Depression | Unspecified style, supervised by qualified instructor, 30 minutes per time, 7 times per week, for 4 weeks. | Psychological: anxiety, depression |
| Liang D 2016 | RCT | Chinese Mainland | Hyperlipidemia | Unspecified style, supervised by qualified instructor, 60 minutes per time, 5 times per week, for 12 weeks. | Laboratory tests: blood lipid; Physical performance: BMI |
| Chen Q 2013 | RCT | Chinese Mainland | Hypertension | Unspecified style, 20 minutes per time, twice per day, 5 times per week, for 12 weeks | Laboratory tests: endothelial function |
| Xu XX 2018 | RCT | Chinese Mainland | Low back pain | The style of State General Administration of Sport of Chinese Mainland in 2003, supervised by qualified instructor, 30 minutes per time, 5 times per week, for 12 weeks. | Symptom: VAS; QOL: SF-36 |
| Cai XN 2017 | RCT | Chinese Mainland | Hypertension | Unspecified style, supervised by qualified instructor, 40 minutes per time, 3 times per week. | Symptom; Physical performance: balance; QOL: WHOQOL-BREF; Psychological: fear of falling |
| Yang H 2014 | RCT | Chinese Mainland | Hypertension | Homemade style, supervised by qualified instructor, 40 minutes per time, 5 times per week, for 24 weeks. | Physical performance: cardiopulmonary function; QOL: SF-36 |
| Yang H 2015 | RCT | Chinese Mainland | Hypertension | Homemade style, supervised by qualified instructor, 40 minutes per time, 5 times per week, for 24 weeks. | Physical performance: cardiopulmonary function |
| Hu L 2018 | RCT | Chinese Mainland | Coronary heart disease | Unspecified style, supervised by qualified instructor, 60 minutes per time, 3 times per week, for 8 weeks. | Laboratory tests: FPG, blood lipid ;Physical performance: blood pressure, cardiopulmonary function; QOL; Psychological: anxiety, depression |
| Wang HQ 2018 | RCT | Chinese Mainland | Coronary heart disease | Unspecified style, 40 minutes per time, 5 times per week, for 12 weeks. | Physical performance: cardiopulmonary function |
| Hua L 2018 | RCT | Chinese Mainland | Coronary heart disease | Homemade style, 30 minutes per session, twice per day, 7 times per week, for 1 weeks. | QOL; Psychological: anxiety |
| Xiong XH 2016 | RCT | Chinese Mainland | Coronary heart disease | Unspecified style, supervised by qualified instructor, 30 minutes per time, 5 times per week, for 12 weeks. | Laboratory tests: cardiac function; QOL |
| Xiong XH 2017 | RCT | Chinese Mainland | Coronary heart disease | Unspecified style, supervised by qualified instructor, 30 minutes per time, 5 times per week, for 12 weeks. | Symptom; QOL |
| Lin J 2012 | RCT | Chinese Mainland | Coronary heart disease | The style of State General Administration of Sport of Chinese Mainland in 2003, 40 minutes per time, 5 times per week, for 12 weeks. | QOL; Psychological: anxiety, depression |
| Zhang HY 2019 | RCT | Chinese Mainland | Low back pain | The style of State General Administration of Sport of Chinese Mainland in 2003, supervised by qualified instructor, 5 times per week, for 10 weeks. | Symptom: VAS, range of motion |
| Zhang ZL 2019 | RCT | Chinese Mainland | Coronary heart disease | The style of State General Administration of Sport of Chinese Mainland in 2003, supervised by qualified instructor, 40 minutes per time, 5 times per week, for 12 weeks. | Laboratory tests: cardiac function; QOL |
| He XW 2014 | RCT | Chinese Mainland | Neck pain | The style of State General Administration of Sport of Chinese Mainland in 2003, 50 to 60 minutes per time, 5 times per week, for 12 weeks. | Symptom: VAS, NDI; Psychological: anxiety, depression |
| Jiang YH 2019 | RCT | Chinese Mainland | Hypertension | The style of State General Administration of Sport of Chinese Mainland in 2003, supervised by qualified instructor, 30 minutes per session, twice per day, 7 times per week, for 12 weeks. | Physical performance: blood pressure; Psychological: anxiety |
| Chen YZ 2016 | RCT | Chinese Mainland | Coronary heart disease | Unspecified style, supervised by qualified instructor, 60 minutes per time, 3 times per week, for 12 weeks. | Psychological: anxiety, depression |
| Li RL 2017 | RCT | Chinese Mainland | Heart failure | Unspecified style, supervised by qualified instructor, for 8 weeks. | Physical performance: pulmonary function; QOL |
| Du WY 2017 | RCT | Chinese Mainland | Falls Prevention | The style of State General Administration of Sport of Chinese Mainland in 2003, 60 minutes per time, 5 times per week, for 24 weeks. | Physical performance: balance |
| Zhang LF 2012 | RCT | Chinese Mainland | Health promotion | Unspecified style, 60 minutes per time, 3 to 5 times per week, for 24 weeks. | Physical performance: balance, strength |
| Sun L 2018 | RCT | Chinese Mainland | Health promotion | Unspecified style, supervised by qualified instructor, 40 to 60 minutes per time, 4 to 5 times per week, for 12 weeks. | Physical performance: balance, strength |
| Zhou Y 2014 | RCT | Chinese Mainland | Health promotion | Unspecified style, 50 minutes per time, 4 times per week, for 12 weeks. | Laboratory tests: immunity; Physical performance: balance, strength |
| Xu ZL 2019 | RCT | Chinese Mainland | Cognitive impairment | The style of State General Administration of Sport of Chinese Mainland in 2003, 50 minutes per time, 6 times per week, for 24 weeks. | Symptom: Cognitive function; Laboratory tests: neuron-specific enolase |
| Zhu HM 2015 | RCT | Chinese Mainland | Type 2 diabetes | The style of State General Administration of Sport of Chinese Mainland in 2003, supervised by qualified instructor, 40 minutes per session, twice per day, 5 times per week, for 48 weeks. | Symptom: Cognitive function; QOL |
| Zhang C 2015 | RCT | Chinese Mainland | Rheumatoid arthritis | Unspecified style, supervised by qualified instructor, 20 minutes per session, twice per day, 7 times per week, for 12 weeks. | Symptom |
| Gao F 2018 | RCT | Chinese Mainland | Low back pain | The style of State General Administration of Sport of Chinese Mainland in 2003, supervised by qualified instructor, 15 minutes per session, twice per day, 6 times per week, for 8 weeks. | Symptom: VAS, ODI; Psychological: anxiety, depression |
| Shi ZB 2018 | RCT | Chinese Mainland | Heart failure | The style of State General Administration of Sport of Chinese Mainland in 2003, 15 minutes per session, twice per day, 7 times per week, for 4 weeks. | Laboratory tests: cardiopulmonary function; QOL |
| Qiu WF 2015 | RCT | Chinese Mainland | COPD | Deng Tietao style, supervised by qualified instructor, 30 minutes per time, 5 times per week, for 12 weeks. | Physical performance: pulmonary function |
| Ye XP 2016 | RCT | Chinese Mainland | COPD | The style of State General Administration of Sport of Chinese Mainland in 2003, 40 minutes per time, 5 times per week, for 4 weeks. | Physical performance: pulmonary function; QOL |
| Zhang HL 2016 | RCT | Chinese Mainland | COPD | Unspecified style, supervised by qualified instructor, 30 minutes per time, 4 times per week, for 24 weeks. | QOL: WHOQOL-BREF |
| Guo JC 2016 | RCT | Chinese Mainland | COPD | The style of State General Administration of Sport of Chinese Mainland in 2003, supervised by qualified instructor, 30 minutes per time, 4 times per week, for 24 weeks. | Laboratory tests: blood gas analysis; Physical performance: pulmonary function |
| Guo XJ 2016 | RCT | Chinese Mainland | COPD | The style of State General Administration of Sport of Chinese Mainland in 2003, supervised by qualified instructor, 30 minutes per time, 4 times per week, for 36 weeks. | Physical performance: pulmonary function; QOL: WHOQOL-BREF |
| Liu M 2019 | RCT | Chinese Mainland | Helicobacter pylori infection | Unspecified style, supervised by qualified instructor, 30 minutes per session, twice per day, 7 times per week, for 12 weeks. | Symptom: PSQI |
| Han JJ 2019 | RCT | Chinese Mainland | Craniocerebral trauma | The style of State General Administration of Sport of Chinese Mainland in 2003, supervised by qualified instructor, 40 minutes per time, 4 times per week, for 24 weeks. | Symptom: Cognitive function; Psychological: anxiety, depression |
| Xie BJ 2019 | RCT | Chinese Mainland | Stroke | Unspecified style, 25 minutes per time, 5 times per week, for 3 weeks. | Physical performance: motor, balance; QOL |
| Kang M 2018 | RCT | Chinese Mainland | Dysmenorrhea | Unspecified style, supervised by qualified instructor, 60 minutes per time, 3 times per week, for 12 weeks. | Symptom: VAS; Psychological: SCL-90 |
| He H 2016 | RCT | Chinese Mainland | Psoriasis | The style of State General Administration of Sport of Chinese Mainland in 2003, 40 minutes per time, 5 times per week, for 4 weeks. | Symptom; QOL: SF-36 |
| Li YJ 2012 | RCT | Chinese Mainland | Ankylosing spondylitis | Unspecified style, 15 to 20 minutes per session, twice per day, 7 times per week, for 12 weeks. | Laboratory tests |
| Liu T 2018 | RCT | Chinese Mainland | Cognitive impairment | The style of State General Administration of Sport of Chinese Mainland in 2003, supervised by qualified instructor, 60 minutes per time, 6 times per week, for 24 weeks. | Symptom: Cognitive function; Laboratory tests |
| Xia R 2017 | RCT | Chinese Mainland | Cognitive impairment | The style of State General Administration of Sport of Chinese Mainland in 2003, supervised by qualified instructor, 60 minutes per time, 3 times per week, for 24 weeks. | Symptom: Cognitive function, attention |
| Li YH 2006 | RCT | Chinese Mainland | Cardiovascular diseases | Unspecified style, supervised by qualified instructor, 40 minutes per time, 5 times per week, for 10 weeks. | Laboratory tests: cardiac function |
| Li Q 2017 | RCT | Chinese Mainland | Breast cancer | The style of State General Administration of Sport of Chinese Mainland in 2003, supervised by qualified instructor, 5 times per week, for 12 weeks. | QOL; Psychological: anxiety, depression |
| Huang YQ 2017 | RCT | Chinese Mainland | Breast cancer | Unspecified style, supervised by qualified instructor, 30 minutes per session, twice per day, 7 times per week, for 16 weeks. | QOL; Psychological: depression |
| Wang XL 2019 | RCT | Chinese Mainland | Type 2 diabetes | The style of Chinese Traditional Sport Health Preservation, supervised by qualified instructor, 30 minutes per time, 5 times per week, for 48 weeks. | Laboratory tests: FPG, HbA1c; Physical performance: BMI |
| Yang H 2018 | RCT | Chinese Mainland | Type 2 diabetes | Unspecified style, supervised by qualified instructor, 30 minutes per time, 5 times per week, for 24 weeks. | Laboratory tests: FPG, HbA1c, blood lipid |
| Li P 2017 | RCT | Chinese Mainland | Type 2 diabetes | Unspecified style, supervised by qualified instructor, 30 minutes per time, 5 times per week, for 24 weeks. | Laboratory tests: FPG, HbA1c, blood lipid |
| Chen B 2015 | RCT | Chinese Mainland | Stroke | The style of State General Administration of Sport of Chinese Mainland in 2003, supervised by qualified instructor, 60 minutes per time, 5 times per week, for 12 weeks. | Symptom: PSQI; Physical performance: cardiopulmonary function; Psychological |
| Ma SH 2011 | RCT | Chinese Mainland | Functional constipation | Unspecified style, supervised by qualified instructor, 60 minutes per time, 5 times per week, for 12 weeks. | QOL: SF-36 |
| Wang J 2017 | RCT | Chinese Mainland | Cardiovascular diseases | The style of State General Administration of Sport of Chinese Mainland in 2003, supervised by qualified instructor, 30 minutes per time, 5 times per week, for 16 weeks. | Symptom; Physical performance |
| Yang YF 2016 | RCT | Chinese Mainland | Hyperlipidemia | The style of State General Administration of Sport of Chinese Mainland in 2003, supervised by qualified instructor, 20 minutes per time, 7 times per week, for 24 weeks. | Laboratory tests: blood lipid |
| Feng L 2017 | RCT | Chinese Mainland | Neck pain | The style of State General Administration of Sport of Chinese Mainland in 2003, supervised by qualified instructor, 20 minutes per session, twice per day, 6 times per week, for 4 weeks. | Symptom: VAS |
| Wang L 2018 | RCT | Chinese Mainland | Insomnia | Unspecified style, supervised by qualified instructor, twice per day, 7 times per week, for14 weeks. | Symptom |
| Li WH 2019 | RCT | Chinese Mainland | Type 2 diabetes | Unspecified style, 40 minutes per time, 3 times per week, for 24 weeks. | Laboratory tests: FPG, HbA1c; Physical performance: blood pressure |
| Zhu HL 2017 | RCT | Chinese Mainland | Type 2 diabetes | Unspecified style, 60 minutes per time, 7 times per week, for 12 weeks. | Laboratory tests: FPG, HbA1c; Psychological: anxiety, depression |
| Fu JM 2018 | RCT | Chinese Mainland | Cognitive impairment | The style of State General Administration of Sport of Chinese Mainland in 2003, 45 minutes per time, 3 times per week, for 12 weeks. | Symptom: Cognitive function |
| Ma SH 2010 | RCT | Chinese Mainland | Perimenopausal syndrome | The style of State General Administration of Sport of Chinese Mainland in 2003, supervised by qualified instructor, 45 minutes per time, 5 times per week, for 12 weeks. | Symptom; Psychological: depression |
| Lei T 2019 | RCT | Chinese Mainland | Lung cancer | Unspecified style, supervised by qualified instructor, 30 minutes per time, 5 times per week, for 8 weeks. | QOL |
| Kang JJ 2018 | RCT | Chinese Mainland | Low back pain | The style of State General Administration of Sport of Chinese Mainland in 2003, 60 minutes per time, 5 times per week, for 24 weeks. | Symptom: VAS, JOA |
| Xu H 2015 | RCT | Chinese Mainland | Low back pain | Unspecified style, 30 minutes per session, twice per day, 5 times per week, for 4 weeks. | Laboratory tests; Symptom: VAS, JOA |
| Zeng XY 2018 | RCT | Chinese Mainland | Low back pain | The style of State General Administration of Sport of Chinese Mainland in 2003, 30 minutes per session, twice per day, 5 times per week, for 8 weeks. | Symptom: VAS, JOA, ODI; Psychological: anxiety |
| Chen H 2012 | RCT | Chinese Mainland | Hypertension | Unspecified style, supervised by qualified instructor, 20 minutes per session, twice per day, 5 times per week, for 24 weeks. | Laboratory tests; Physical performance: blood pressure |
| Chen Y 2017 | RCT | Chinese Mainland | Osteoporosis | Unspecified style, supervised by qualified instructor, 40 to 60 minutes per session, twice per day, 5 times per week, for 8 weeks. | Symptom: VAS; QOL: SF-36 |
| Liu M 2018 | RCT | Chinese Mainland | Insomnia | The style of State General Administration of Sport of Chinese Mainland in 2003, supervised by qualified instructor, 60 minutes per time, 5 times per week, for 12 weeks. | Symptom: PSQI; Psychological: anxiety |
| Liu YJ 2017 | RCT | Chinese Mainland | Low back pain | The style of State General Administration of Sport of Chinese Mainland in 2003, 30 minutes per time, 6 times per week, for 4 weeks. | Symptom: VAS |
| Zhang B 2015 | RCT | Chinese Mainland | Stroke | Unspecified style, supervised by qualified instructor, 40 minutes per session, twice per day, 5 times per week, for 10 weeks. | Symptom; Physical performance: motor, balance |
| Cai YF 2018 | RCT | Chinese Mainland | Neck pain | The style of State General Administration of Sport of Chinese Mainland in 2003, supervised by qualified instructor, 30 minutes per session, twice per day, 5 times per week, for 24 weeks. | Symptom; Psychological |
| Xiu MN 2015 | RCT | Chinese Mainland | Cancer | Unspecified style, supervised by qualified instructor, 30 minutes per session, twice per day, 5 times per week, for 8 weeks. | Symptom; QOL |
| Men HY 2013 | RCT | Chinese Mainland | Stroke | Other style, 60 minutes per time, 7 times per week, for 2 weeks. | Symptom |
| Wu YC 2015 | RCT | Chinese Mainland | Type 2 diabetes | Unspecified style, supervised by qualified instructor, 3 session per day, 5 times per week, for 24 weeks. | Laboratory tests: FPG, HbA1c; QOL: SF-36 |
| Zhang SJ 2015 | RCT | Chinese Mainland | Type 2 diabetes | Unspecified style, 30 minutes per time, 5 times per week, for 12 weeks. | Laboratory tests: FPG, HbA1c |
| Dai GZ 2018 | RCT | Chinese Mainland | COPD | The style of State General Administration of Sport of Chinese Mainland in 2003, supervised by qualified instructor, 30 minutes per time, 3 times per week, for 48 weeks. | Physical performance: pulmonary function |
| Li ZF 2019 | RCT | Chinese Mainland | Osteoporosis | Unspecified style, supervised by qualified instructor, 30 to 40 minutes per time, 5 times per week, for 24 weeks. | Symptom; Physical performance: balance; Psychological: anxiety |
| Zhuang KC | RCT | Chinese Mainland | Stroke | The style of State General Administration of Sport of Chinese Mainland in 2003, supervised by qualified instructor, 50 minutes per time, 5 times per week, for 12 weeks. | Physical performance |
| Du WY 2018 | RCT | Chinese Mainland | Health promotion | The style of State General Administration of Sport of Chinese Mainland in 2003, 45 minutes per time, 5 times per week, for 24 weeks. | Physical performance: balance |
| Wei RB 2012 | RCT | Chinese Mainland | Ankylosing spondylitis | Unspecified style, 15 to 20 minutes per session, twice per day, 7 times per week, for 12 weeks. | Laboratory tests; Symptom: VAS, range of motion; Physical performance |
| Ren YP 2018 | RCT | Chinese Mainland | Type 2 diabetes | Unspecified style, 60 minutes per time, 4 times per week, for 16 weeks. | Physical performance |
| Wang CM 2016 | RCT | Chinese Mainland | Knee osteoarthritis | Unspecified style, 30 minutes per time, 5 times per week, for 12 weeks. | Symptom: VAS; Physical performance |
| Yang YP 2014 | RCT | Chinese Mainland | Arrhythmia | Unspecified style, 20 minutes per session, twice per day, 7 times per week, for 8 weeks. | Physical performance: hotler |
| Qiu YC 2018 | RCT | Chinese Mainland | Knee osteoarthritis | Unspecified style, 30 minutes per time, 5 times per week, for 12 weeks. | Symptom: VAS; Physical performance |
| Wan J 2013 | RCT | Chinese Mainland | Neck pain | The style of Chinese Traditional Sport Health Preservation, supervised by qualified instructor, 30 minutes per session, twice per day, 5 times per week, for 5 weeks. | Symptom: VAS; Psychological |
| Shan SQ 2015 | RCT | Chinese Mainland | Health promotion | Unspecified style, 60 minutes per time, 5 times per week, for 4 weeks. | Physical performance |
| Peng XL 2018 | RCT | Chinese Mainland | Addiction | Unspecified style, supervised by qualified instructor, 30 minutes per time, 5 times per week, for 20 weeks. | Psychological: anxiety, depression |
| Zhuang Q 2017 | RCT | Chinese Mainland | Type 2 diabetes | Unspecified style, supervised by qualified instructor, 40 minutes per time, 5 times per week, for 24 weeks. | Laboratory tests: FPG, HbA1c, blood lipid; Physical performance |
| Yin G 2013 | RCT | Chinese Mainland | COPD | Deng Tietao style, 30 minutes per time, 5 times per week, for 24 weeks. | Physical performance: pulmonary function; QOL |
| Xie ZR 2018 | RCT | Chinese Mainland | Health promotion | The style of State General Administration of Sport of Chinese Mainland in 2003, supervised by qualified instructor, 30 to 50 minutes per time, 3 times per week, for 12 weeks. | Symptom: VAS, NDI, range of motion; QOL: SF-36 |
| Li WH 2019 | RCT | Chinese Mainland | Type 2 diabetes | Unspecified style, 20 minutes per time, 3 times per week, for 36 weeks. | Laboratory tests: FPG; Physical performance |
| Geng YQ 2013 | RCT | Chinese Mainland | Health promotion | Unspecified style, supervised by qualified instructor, 30 minutes per time, 3 times per week, for 4 weeks. | Psychological: SCL-90 |
| Lv F 2015 | RCT | Chinese Mainland | Breast cancer | Unspecified style, 60 minutes per time, 3 times per week, for 24 weeks. | Symptom; QOL: SF-36 |
| Wang C 2009 | RCT | Chinese Mainland | Frozen shoulder | The style of State General Administration of Sport of Chinese Mainland in 2003, 30 minutes per session, twice per day, 5 times per week, for 5 weeks. | Symptom: VAS, range of motion |
| Yang D 2019 | RCT | Chinese Mainland | Parkinson's Disease | Unspecified style, for 8 weeks. | Symptom; Physical performance: balance |
| Zhang JM 2016 | RCT | Chinese Mainland | Health promotion | The style of State General Administration of Sport of Chinese Mainland in 2003, supervised by qualified instructor, 90 minutes per time, 3 times per week, for 8 weeks. | Physical performance: pulmonary function |
| Wang YW 2019 | RCT | Chinese Mainland | Insomnia | Unspecified style, 45 minutes per time, 4 times per week, for 12 weeks. | Symptom: PSQI; Physical performance: memory function |
| Lin Q 2017 | RCT | Chinese Mainland | Cognitive impairment | Unspecified style, 6 times per week, for 24 weeks. | Laboratory tests: cerebrospinal fluid; Physical performance: cognitive function; QOL; Psychological: depression |
| Chen X 2019 | RCT | Chinese Mainland | Stable angina | The style of State General Administration of Sport of Chinese Mainland in 2003, 36 minutes per time, 5 times per week, for 12 weeks. | Symptom; QOL; Psychological: anxiety, depression |
| Wang CY 2015 | RCT | Chinese Mainland | Type 2 diabetes | The style of State General Administration of Sport of Chinese Mainland in 2003, supervised by qualified instructor, 20 minutes per time, 5 times per week, for 6 weeks. | Laboratory tests: FPG, HbA1c, blood lipid |
| Jin YY 2017 | RCT | Chinese Mainland | Insomnia | The style of State General Administration of Sport of Chinese Mainland in 2003, supervised by qualified instructor, 45 minutes per time, 5 times per week, for 4 weeks. | Symptom |
| Zhang RR 2013 | RCT | Chinese Mainland | Type 2 diabetes | The style of State General Administration of Sport of Chinese Mainland in 2003, supervised by qualified instructor, 60 minutes per time, 5 times per week, for 48 weeks. | Laboratory tests: FPG, HbA1c, blood lipid; QOL; Psychological: anxiety, depression |
| Sun YJ 2015 | RCT | Chinese Mainland | Type 2 diabetes | Homemade style, supervised by qualified instructor, 60 minutes per time, 5 times per week, for 24 weeks. | Laboratory tests: FPG, HbA1c, blood lipid; Psychological: anxiety, depression |
| Huang YY 2013 | RCT | Chinese Mainland | COPD | Unspecified style, supervised by qualified instructor, 15 minutes per time, 7 times per week, for 12 weeks. | Physical performance: lung function |
| Bai YJ 2011 | RCT | Chinese Mainland | Stroke | The style of State General Administration of Sport of Chinese Mainland in 2003, 20 minutes per session, twice per day, 5 times per week, for 6 weeks. | Physical performance: balance |
| Zhang XY 2018 | RCT | Chinese Mainland | Atrial fibrillation | Unspecified style, supervised by qualified instructor, 20 to 30 minutes per time, 5 times per week, for 12 weeks. | QOL: SF-36; Psychological: anxiety |
| Li MS 2012 | RCT | Chinese Mainland | Hyperlipidemia | Deng Tietao style, supervised by qualified instructor, twice per day, once per week, for 48 weeks. | Laboratory tests: blood lipid; Physical performance: BMI |
| Zhu XY 2019 | RCT | Chinese Mainland | Insomnia | The style of State General Administration of Sport of Chinese Mainland in 2003, supervised by qualified instructor, 30 minutes per time, 5 times per week, for 4 weeks. | Symptom: PSQI |
| Zhang Y 2016 | RCT | Chinese Mainland | Stroke | Unspecified style, 20 minutes per session, twice per day, 5 times per week, for 8 weeks. | Symptom; Physical performance: motor, balance |
| Wang J 2016 | RCT | Chinese Mainland | Type 2 diabetes | Unspecified style, supervised by qualified instructor, 30 minutes per time, 5 times per week, for 4 weeks. | Symptom; Psychological: anxiety, depression |
| Xiao HZ 2018 | RCT | Chinese Mainland | Psoriasis | Homemade style, supervised by qualified instructor, 40 minutes per time, 5 times per week, for 12 weeks. | Symptom; QOL; Psychological: depression |
| Li XY 2018 | RCT | Chinese Mainland | Heart failure | Unspecified style, supervised by qualified instructor, 30 minutes per time, 5 times per week, for 12 weeks. | Symptom; Physical performance: cardiopulmonary function; QOL |
| Zhu YJ 2018 | RCT | Chinese Mainland | Heart disease | Other style, 60 minutes per time, 5 times per week. | Symptom; Physical performance: pulmonary function; QOL |
| Zhang XY 2018 | RCT | Chinese Mainland | Angina | The style of State General Administration of Sport of Chinese Mainland in 2003, 20 to 30 minutes per time, 5 times per week, for 24 weeks. | Symptom; Laboratory tests; Physical performance: cardiopulmonary function; QOL: SF-36 |
| Li YS 2018 | RCT | Chinese Mainland | Heart disease | Unspecified style, supervised by qualified instructor, 40 minutes per time, 6 times per week, for 4 weeks. | Laboratory tests: cardiac function |
| Zhang HY 2019 | RCT | Chinese Mainland | Insomnia | Unspecified style, 20 minutes per session, twice per day, 5 times per week, for 4 weeks. | Symptom: PSQI |
| Xiong GH 2015 | RCT | Chinese Mainland | Insomnia | The style of State General Administration of Sport of Chinese Mainland in 2003, 20 minutes per session, twice per day, 5 times per week, for 4 weeks. | Symptom: PSQI |
| Xiong GH 2016 | RCT | Chinese Mainland | Insomnia | The style of State General Administration of Sport of Chinese Mainland in 2003, 20 minutes per session, twice per day, 5 times per week, for 4 weeks. | Symptom |
| Li PC 2019 | RCT | Chinese Mainland | Neck pain | The style of State General Administration of Sport of Chinese Mainland in 2003, supervised by qualified instructor, 60 minutes per time, 5 times per week, for 4 weeks. | Symptom: VAS |
| Gao HX 2019 | RCT | Chinese Mainland | Neck pain | The style of State General Administration of Sport of Chinese Mainland in 2003, supervised by qualified instructor, 5 times per week, for 4 weeks. | Symptom: VAS |
| Feng B 2019 | RCT | Chinese Mainland | Low back pain | The style of State General Administration of Sport of Chinese Mainland in 2003, supervised by qualified instructor, 10 minutes per time, 5 times per week, for 4 weeks. | Symptom: VAS, ODI |
| Liu J 2012 | RCT | Chinese Mainland | Ankylosing spondylitis | Unspecified style, supervised by qualified instructor, for 12 weeks. | Laboratory tests |
| Yin HY 2016 | RCT | Chinese Mainland | Type 2 diabetes | The style of State General Administration of Sport of Chinese Mainland in 2003, supervised by qualified instructor, 60 minutes per time, 5 times per week, for 24 weeks. | Laboratory tests: FPG, HbA1c, blood lipid; Psychological: anxiety, depression |
| Cai YX 2018 | RCT | Chinese Mainland | Osteoporosis | The style of State General Administration of Sport of Chinese Mainland in 2003, supervised by qualified instructor, 30 minutes per session, twice per day, 5 times per week, for 48 weeks. | Laboratory tests; Physical performance: BMD; QOL |
| Qian J 2018 | RCT | Chinese Mainland | COPD | Unspecified style, supervised by qualified instructor, 30 minutes per time, 5 times per week, for 12 weeks. | Physical performance: cardiopulmonary function; QOL; Psychological: anxiety, depression |
| Hou DF 2017 | RCT | Chinese Mainland | COPD | Unspecified style, supervised by qualified instructor, 30 minutes per time, 2 times per week, for 12 weeks. | Laboratory tests; Physical performance: pulmonary function |
| Huang F 2016 | RCT | Chinese Mainland | COPD | The style of State General Administration of Sport of Chinese Mainland in 2003, supervised by qualified instructor, 30 minutes per time, 2 times per week, for 12 weeks. | Laboratory tests; Physical performance: pulmonary function |
| Yang M 2013 | RCT | Chinese Mainland | Hypertension | The style of State General Administration of Sport of Chinese Mainland in 2003, supervised by qualified instructor, for 48 weeks. | Laboratory tests: blood glucose, blood lipid; Physical performance: blood pressure |
| Cai YF 2016 | RCT | Chinese Mainland | Hypertension | The style of State General Administration of Sport of Chinese Mainland in 2003, supervised by qualified instructor, 30 to 40 minutes per time, 5 times per week, for 48 weeks. | Physical performance: blood pressure, BMI |
| Zhu WX 2019 | RCT | Chinese Mainland | Type 2 diabetes | Unspecified style, supervised by qualified instructor, 5 times per week, for 24 weeks. | Physical performance: heart rate |
| Guo J 2013 | RCT | Chinese Mainland | Stroke | Unspecified style, 20 minutes per session, twice per day, 5 times per week, for 6 weeks. | Symptom |
| Zhang M 2013 | RCT | Chinese Mainland | Stroke | Unspecified style, 20 minutes per session, twice per day, 5 times per week, for 6 weeks. | Symptom: balance |
| Su JH 2018 | RCT | Chinese Mainland | Osteoporosis | Unspecified style, supervised by qualified instructor, 45 to 60 minutes per session, twice per day, 5 times per week, for 24 weeks. | Symptom; Physical performance: BMD, balance |
| Qiu P 2019 | RCT | Chinese Mainland | Cancer | Unspecified style, supervised by qualified instructor, 15 to 20 minutes per session, twice per day, 5 times per week, for 4 weeks. | Symptom; Psychological: anxiety, depression |
| Wei DL 2019 | RCT | Chinese Mainland | Insomnia | The style of State General Administration of Sport of Chinese Mainland in 2003, supervised by qualified instructor, 15 minutes per session, twice per day, 5 times per week, for 8 weeks. | Symptom: PSQI; QOL: SF-36; Psychological: anxiety, depression |
| Gao Y 2018 | RCT | Chinese Mainland | Functional constipation | The style of State General Administration of Sport of Chinese Mainland in 2003, supervised by qualified instructor, 30 minutes per session, twice per day, 5 times per week, for 12 weeks. | Symptom; Laboratory tests; QOL |
| Sun J 2019 | RCT | Chinese Mainland | Breast cancer | Unspecified style, 12 minutes per time, 1 times per week, for 4 weeks. | Laboratory tests: immunity; QOL; Psychological: anxiety, depression |
| Chen M 2017 | RCT | Chinese Mainland | Leukemia | Unspecified style, supervised by qualified instructor, 30 minutes per session, twice per day, 5 times per week, for 4 weeks. | Symptom; Psychological: anxiety, depression |
| Liao XH 2018 | RCT | Chinese Mainland | Hypertension | The style of State General Administration of Sport of Chinese Mainland in 2003, supervised by qualified instructor, 60 minutes per time, 6 times per week, for 4 weeks. | Symptom: PSQI; Laboratory tests: polysomnogram; Physical performance: blood pressure |
| Fu JY 2016 | RCT | Chinese Mainland | Ankylosing spondylitis | Unspecified style, supervised by qualified instructor, 30 minutes per session, twice per day, 5 times per week, for 24 weeks. | Symptom: VAS; Laboratory tests; Physical performance; Psychological:SCL-90 |
| Li XY 2019 | RCT | Chinese Mainland | Heart failure | The style of State General Administration of Sport of Chinese Mainland in 2003, supervised by qualified instructor, 20 to 30 minutes per time, 5 times per week, for 12 weeks. | Laboratory tests; QOL |
| Chen GJ 2018 | RCT | Chinese Mainland | Chronic gastritis | Unspecified style, supervised by qualified instructor, 45 minutes per time, 5 times per week, for 48 weeks. | Psychological: anxiety, depression |
| Li L 2015 | RCT | Chinese Mainland | Low back pain | The style of State General Administration of Sport of Chinese Mainland in 2003, supervised by qualified instructor, 30 minutes per session, twice per day, 5 times per week, for 8 weeks. | Symptom: VAS, ODI |
| Wang LQ 2016 | RCT | Chinese Mainland | Type 2 diabetes | Unspecified style, supervised by qualified instructor, 20 minutes per time, 4times per week, for 12 weeks. | Laboratory tests: FPG |
| Cao BL 2015 | RCT | Chinese Mainland | Type 2 diabetes | Unspecified style, 20 minutes per session, twice per day, 5 times per week, for 8 weeks. | Symptom: VAS; Laboratory tests: FPG, HbA1c |
| Wu Y 2019 | RCT | Chinese Mainland | COPD | The style of State General Administration of Sport of Chinese Mainland in 2003, 30 minutes per time, 5 times per week, for 12 weeks. | Physical performance: pulmonary function; QOL |
| Wei W 2017 | RCT | Chinese Mainland | Metabolic diseases | The style of State General Administration of Sport of Chinese Mainland in 2003, supervised by qualified instructor, 45 minutes per session, twice per day, 6 times per week, for 24 weeks. | Laboratory tests: blood glucose, blood lipid; Physical performance: blood pressure, BMI |
| Han BL 2015 | RCT | Chinese Mainland | Low back pain | The style of State General Administration of Sport of Chinese Mainland in 2003, 40 minutes per time, 5 times per week, for 4 weeks. | Symptom: VAS, ODI |
| Li CN 2015 | RCT | Chinese Mainland | Low back pain | Unspecified style, 20 to 30 minutes per time, 5 times per week, for 4 weeks. | Symptom: VAS, ODI |
| Fu MF 2019 | RCT | Chinese Mainland | Neck pain | The style of State General Administration of Sport of Chinese Mainland in 2003, supervised by qualified instructor, 60 minutes per time, 5 times per week, for 6 weeks. | Symptom: VAS, NDI |
| Wu W 2013 | RCT | Chinese Mainland | Dysmenorrhea | Unspecified style, 60 minutes per time, 5 times per week, for 6 weeks. | Symptom |
| Guan L2019 | RCT | Chinese Mainland | Lung cancer | Unspecified style, supervised by qualified instructor, twice per day, 5 times per week, for 9weeks. | Symptom: PSQI; QOL; Psychological: anxiety, depression |
| Lu HJ 2019 | RCT | Chinese Mainland | Chronic fatigue syndrome | The style of State General Administration of Sport of Chinese Mainland in 2003, supervised by qualified instructor, 30 minutes per time, 5 times per week, for 12 weeks. | Symptom |
| Chen L 2018 | RCT | Chinese Mainland | Psoriasis | Unspecified style, 30 minutes per time, 5 times per week, for 4 weeks. | Symptom; Laboratory tests; Psychological: anxiety, depression |
| Hao J 2013 | RCT | Chinese Mainland | Perimenopausal syndrome | Unspecified style, 45 minutes per time, 5 times per week, for 20 weeks. | Symptom; QOL: SF-36 |
| Liu YY 2018 | RCT | Chinese Mainland | Knee osteoarthritis | Unspecified style, supervised by qualified instructor, 30 minutes per time, 5 times per week, for 4 weeks. | Symptom; Physical performance |
| Sun LJ 2017 | RCT | Chinese Mainland | Knee osteoarthritis | The style of Chinese Health Qigong Association, supervised by qualified instructor, 20 to 30 minutes per session, twice per day, 5 times per week, for 4 weeks. | Symptom: VAS; Physical performance |
| Yang BY 2016 | RCT | Chinese Mainland | COPD | Unspecified style, supervised by qualified instructor, 30 minutes per time, 6 times per week, for 12 weeks. | Physical performance: pulmonary function |
| Zhang XX 2019 | RCT | Chinese Mainland | Heart disease | Unspecified style, supervised by qualified instructor, 30 minutes per session, twice per day, 5 times per week, for 24 weeks. | QOL |
| Han Y 2017 | RCT | Chinese Mainland | Lung cancer | The style of State General Administration of Sport of Chinese Mainland in 2003, supervised by qualified instructor, 30 minutes per time, 3 to 5 times per week, for 12 weeks. | Symptom; Physical performance: pulmonary function; QOL; Psychological: anxiety, depression |
| Han Y 2017 | RCT | Chinese Mainland | Breast cancer | The style of State General Administration of Sport of Chinese Mainland in 2003, supervised by qualified instructor, 20 minutes per time, 5 times per week, for 12 weeks. | Laboratory tests; Psychological: anxiety |
| Chen X 2018 | RCT | Chinese Mainland | Lung cancer | Unspecified style, supervised by qualified instructor, 30 minutes per time, 5 times per week, for 8 weeks. | QOL |
| Wu YM 2011 | RCT | Chinese Mainland | Type 2 diabetes | The style of State General Administration of Sport of Chinese Mainland in 2003, for 24 weeks. | Laboratory tests: blood glucose; QOL: SF-36 |
| Chen XH 2018 | RCT | Chinese Mainland | Type 2 diabetes | Unspecified style, supervised by qualified instructor, 20 minutes per time, 5 times per week, for 24 weeks. | Laboratory tests: FPG, HbA1c |
| Yu ML 2018 | RCT | Chinese Mainland | Heart failure | The style of State General Administration of Sport of Chinese Mainland in 2003, supervised by qualified instructor, 45 minutes per time, 5 times per week, for 12 weeks. | Physical performance: cardiopulmonary function; QOL |
| Zhang XQ 2018 | RCT | Chinese Mainland | Cancer | Unspecified style, supervised by qualified instructor, 30 minutes per time, 5 times per week, for 8 weeks. | Symptom: PSQI; Physical performance: heart rate |
| Zhang H 2015 | RCT | Chinese Mainland | Health promotion | Unspecified style, 13 minutes per session, twice per day, 5 times per week, for 12 weeks. | Laboratory tests: pulmonary function |
| Guan FP 2016 | RCT | Chinese Mainland | Schizophrenia | Unspecified style, supervised by qualified instructor, 30 minutes per time, 5 times per week, for 24 weeks. | Psychological: anxiety |
| Shang YH 2017 | RCT | Chinese Mainland | Type 2 diabetes | The style of State General Administration of Sport of Chinese Mainland in 2003, supervised by qualified instructor, 60 minutes per time, 5 times per week, for 24 weeks. | Laboratory tests: blood glucose, blood lipid; Physical performance: BMI |
| Liu HH 2014 | RCT | Chinese Mainland | Type 2 diabetes | The style of State General Administration of Sport of Chinese Mainland in 2003, 30 minutes per time, 5 times per week, for 24 weeks. | Laboratory tests: HbA1c; Psychological: anxiety |
| Pan HS 2008 | RCT | Chinese Mainland | Type 2 diabetes | The style of State General Administration of Sport of Chinese Mainland in 2003, 45 minutes per session, twice per day, 5 times per week, for 24 weeks. | Laboratory tests: blood glucose, blood lipid; Physical performance: BMI, pulmonary function |
| Duan JH 2012 | RCT | Chinese Mainland | Type 2 diabetes | Unspecified style, 20 minutes per time, 5 times per week, for 8 weeks. | Laboratory tests: FPG, HbA1c, blood lipid; Physical performance: BMI |
| Wu LM 2019 | RCT | Chinese Mainland | Neck pain | The style of State General Administration of Sport of Chinese Mainland in 2003, 30 to 40 minutes per time, 3 to 5 times per week, for 8 weeks. | Symptom: VAS, NDI, range of motion |
| Yi WM 2019 | RCT | Chinese Mainland | Type 2 diabetes | The style of State General Administration of Sport of Chinese Mainland in 2003, supervised by qualified instructor, 30 minutes per session, 3 sessions per day, 5 times per week, for 12 weeks. | Laboratory tests: FPG, HbA1c, blood lipid |
| Miao HY 2018 | RCT | Chinese Mainland | Type 2 diabetes | Unspecified style, 20 minutes per session, twice per day, 5 times per week, for 12 weeks. | QOL |
| Hou JY 2016 | RCT | Chinese Mainland | Type 2 diabetes | Unspecified style, supervised by qualified instructor, 30 minutes per time, 5 times per week, for 24 weeks. | Laboratory tests: FPG, HbA1c, blood lipid |
| Huang YY 2019 | RCT | Chinese Mainland | Schizophrenia | Unspecified style, supervised by qualified instructor, 30 minutes per session, twice per day, 5 times per week, for 12 weeks. | Symptom: PSQI; Laboratory tests: FPG, HbA1c, blood lipid; Physical performance: BMI |
| Lin XL 2012 | RCT | Chinese Mainland | Heart disease | Homemade style, supervised by qualified instructor, 30 to 45 minutes per session, twice per day, 5 times per week, for 20 weeks. | QOL |
| Liu XY 2014 | RCT | Chinese Mainland | Falls Prevention | Unspecified style, supervised by qualified instructor, 30 to 40 minutes per time, 5 times per week, for 12 weeks. | Physical performance: balance |
| Wang XK 2012 | RCT | Chinese Mainland | Osteoporosis | Unspecified style, 50 minutes per session, twice per day, 5 times per week, for 16 weeks. | Laboratory tests; Physical performance: BMD |
| Zheng LW 2014 | RCT | Chinese Mainland | Hypertension | The style of State General Administration of Sport of Chinese Mainland in 2003, 30 minutes per time, 5 times per week, for 12 weeks. | Laboratory tests; Physical performance: blood pressure |
| Feng YC 2010 | RCT | Chinese Mainland | Irritable bowel syndrome | The style of State General Administration of Sport of Chinese Mainland in 2003, supervised by qualified instructor, 45 minutes per session, twice per day, 5 times per week, for 12 weeks. | Symptom |
| Pan HS 2010 | RCT | Chinese Mainland | Chronic nephritis | The style of State General Administration of Sport of Chinese Mainland in 2003, supervised by qualified instructor, 45 minutes per session, twice per day, 5 times per week, for 12 weeks. | Laboratory tests |
| Zhai FM 2013 | RCT | Chinese Mainland | Health promotion | Unspecified style, supervised by qualified instructor, 40 to 50 minutes per time, 5 times per week, for 8 weeks. | Physical performance: blood pressure, balance, strength |
| Feng YC 2009 | RCT | Chinese Mainland | COPD | The style of State General Administration of Sport of Chinese Mainland in 2003, supervised by qualified instructor, 45 minutes per session, twice per day, 5 times per week, for 24 weeks. | Laboratory tests: blood gas analysis; Physical performance: pulmonary function |
| Guo JC 2016 | RCT | Chinese Mainland | COPD | Unspecified style, supervised by qualified instructor, 15 to 20 minutes per time, 4 times per week, for 24 weeks. | Laboratory tests: blood gas analysis; Physical performance: pulmonary function |
| Zheng YH 2018 | RCT | Chinese Mainland | Stroke | The style of State General Administration of Sport of Chinese Mainland in 2003, supervised by qualified instructor, 40 minutes per time, 3 times per week, for 24 weeks. | Symptom: cognitive function; QOL: SF-36 |
| Xiong ZY 2018 | RCT | Chinese Mainland | Stroke | The style of State General Administration of Sport of Chinese Mainland in 2003, supervised by qualified instructor, 40 minutes per time, 3 times per week, for 24 weeks. | Physical performance: cognitive function, motor function, balance, muscle tension |
| Wei YJ 2018 | RCT | Chinese Mainland | Addiction | Unspecified style, 60 minutes per time, 3 times per week, for 16 weeks. | Physical performance; Psychological: anxiety, depression |
| Li SZ 2016 | RCT | Chinese Mainland | Cognitive impairment | The style of State General Administration of Sport of Chinese Mainland in 2003, supervised by qualified instructor, 60 minutes per time, 3 times per week, for 24 weeks. | Physical performance: cognitive function; QOL: SF-36 |
| Xiong ZY 2018 | RCT | Chinese Mainland | Stroke | The style of State General Administration of Sport of Chinese Mainland in 2003, supervised by qualified instructor, 60 minutes per time, 5 times per week, for 12 weeks. | Symptom: PSQI |
| Fang QY 2015 | RCT | Chinese Mainland | Stroke | The style of State General Administration of Sport of Chinese Mainland in 2003, supervised by qualified instructor, 60 minutes per time, 5 times per week, for 12 weeks. | Laboratory tests: blood glucose, blood lipid; Physical performance: BMI, blood pressure |
| Guan YX 2012 | RCT | Chinese Mainland | Type 2 diabetes | The style of State General Administration of Sport of Chinese Mainland in 2003, supervised by qualified instructor, 60 minutes per time, 5 times per week, for 16 weeks. | Laboratory tests: FPG, HbA1c, blood lipid; Psychological: anxiety, depression |
| Zhou LB 2011 | RCT | Chinese Mainland | Type 2 diabetes | Unspecified style, supervised by qualified instructor, 30 minutes per time, 5 times per week, for 12 weeks. | Laboratory tests: FPG, HbA1c, blood lipid; Physical performance: BMI |
| Zha CM 2011 | RCT | Chinese Mainland | viral hepatitis type B | Unspecified style, supervised by qualified instructor, 30 minutes per session, twice per day, 5 times per week, for 8 weeks. | QOL: SF-36 |
| Lin Q 2016 | RCT | Chinese Mainland | Cognitive impairment | The style of State General Administration of Sport of Chinese Mainland in 2003, 60 minutes per time, 5 times per week, for 24 weeks. | Physical performance: cognitive function, |
| Li Y 2017 | RCT | Chinese Mainland | Type 2 diabetes | The style of State General Administration of Sport of Chinese Mainland in 2003, supervised by qualified instructor, 30 minutes per time, 4 times per week, for 12 weeks. | Laboratory tests: FPG, HbA1c, blood lipid; Physical performance: BMI |
| Liang YH 2014 | RCT | Chinese Mainland | Hypertension | The style of State General Administration of Sport of Chinese Mainland in 2003, supervised by qualified instructor, 20 minutes per session, twice per day, 5 times per week, for 24 weeks. | Laboratory tests: blood lipid; Physical performance: blood pressure |
| Chen Y 2017 | RCT | Chinese Mainland | COPD | The style of Beijing Sport University, 30 minutes per time, 5 times per week, for 24 weeks. | Physical performance: pulmonary function; QOL: SF-36 |
| He YC 2018 | RCT | Chinese Mainland | COPD | Unspecified style, twice per day, 5 times per week, for 4 weeks. | Physical performance: pulmonary function; QOL: WHOQOL-BREF |
| Yang R 2018 | RCT | Chinese Mainland | Psoriasis | Unspecified style, supervised by qualified instructor, 30 minutes per time, 5 times per week, for 5 weeks. | Symptom; QOL: SF-36 |
| Li N 2012 | RCT | Chinese Mainland | viral hepatitis type B | Unspecified style, supervised by qualified instructor, 30 minutes per session, twice per day, 5 times per week, for 8 weeks. | Symptom; Physical performance: BMI |
| Yu HL 2013 | RCT | Chinese Mainland | Hypertension | Unspecified style, for 48 weeks. | Physical performance: blood pressure, BMI |
| Shi XY 2017 | RCT | Chinese Mainland | Falls Prevention | Unspecified style, supervised by qualified instructor, 30 to 40 minutes per time, 5 times per week, for 12 weeks. | Physical performance: balance |
| Wang XJ 2019 | RCT | Chinese Mainland | Coronary heart disease | The style of State General Administration of Sport of Chinese Mainland in 2003, supervised by qualified instructor, 20 minutes per session, 3 to 4 sessions per day, 5 times per week. | Symptom; Laboratory tests: blood glucose, blood lipid; Physical performance: blood pressure; QOL: SF-36 |
| Zeng JH 2016 | RCT | Chinese Mainland | Functional dyspepsia | The style of State General Administration of Sport of Chinese Mainland in 2003, supervised by qualified instructor, 40 minutes per session, twice per day, 5 times per week, for 12 weeks. | Symptom; QOL |
| Zhang HZ 2018 | RCT | Chinese Mainland | Frozen shoulder | The style of State General Administration of Sport of Chinese Mainland in 2003, supervised by qualified instructor, 60 minutes per time, 5 times per week, for 12 weeks. | Symptom: VAS, range of motion |
| Huang LS 2017 | RCT | Chinese Mainland | Chronic gastritis | The style of State General Administration of Sport of Chinese Mainland in 2003, supervised by qualified instructor, 30 minutes per time, 5 times per week, for 5 weeks. | QOL: SF-36 |
| Li Q 2017 | RCT | Chinese Mainland | Lung cancer | The style of State General Administration of Sport of Chinese Mainland in 2003, 20 to 30 minutes per time, 3 times per week, for 12 weeks. | Symptom; Physical performance: pulmonary function; Psychological: anxiety, depression |
| Pan XJ 2019 | RCT | Chinese Mainland | Low back pain | The style of State General Administration of Sport of Chinese Mainland in 2003, supervised by qualified instructor, 30 to 40 minutes per session, twice per day, 5 times per week, for 2 weeks. | Symptom: VAS, JOA |
| Chen LH 2016 | RCT | Chinese Mainland | Hypertension | Unspecified style, 60 minutes per time, 5 times per week, for 12 weeks. | Physical performance: blood pressure |
| Pan W 2019 | RCT | Chinese Mainland | Heart failure | Unspecified style, 60 minutes per time, 5 times per week, for82 weeks. | Physical performance: cardiopulmonary function |
| Liu ZL2018 | RCT | Chinese Mainland | COPD | Unspecified style, supervised by qualified instructor, 30 minutes per time, 5 times per week, for 12 weeks. | Physical performance: pulmonary function |
| Yuan SJ 2018 | RCT | Chinese Mainland | COPD | Unspecified style, supervised by qualified instructor, 5 times per week, for 24 weeks. | Physical performance: pulmonary function |
| Sun LJ 2017 | RCT | Chinese Mainland | Knee osteoarthritis | Unspecified style, supervised by qualified instructor, 20 to 30 minutes per session, twice per day, 5 times per week, for 4 weeks. | Physical performance: strength |
| Hou XL 2018 | RCT | Chinese Mainland | Neurasthenia | The style of State General Administration of Sport of Chinese Mainland in 2003, supervised by qualified instructor, 35 to 45 minutes per time, 5 times per week, for 12 weeks. | Symptom; Physical performance: cognitive function, balance; QOL |
| Yao LP 2008 | RCT | Chinese Mainland | Low back pain | Unspecified style, 60 minutes per time, 5 times per week, for 24 weeks. | Symptom: JOA; QOL |
| Xu H 2015 | RCT | Chinese Mainland | Low back pain | Unspecified style, 60 minutes per time, 5 times per week, for 24 weeks. | Symptom: VAS, JOA |
| Shang ZQ 2019 | RCT | Chinese Mainland | Insomnia | Unspecified style, 60 minutes per time, 5 times per week, for 4 weeks. | Symptom |
| Ren JY 2018 | RCT | Chinese Mainland | Insomnia | The style of State General Administration of Sport of Chinese Mainland in 2003, supervised by qualified instructor, 30 minutes per time, 5 times per week, for 4 weeks. | Symptom: PSQI |
| Deng YF 2014 | RCT | Chinese Mainland | COPD | The style of State General Administration of Sport of Chinese Mainland in 2003, 30 minutes per time, 5 times per week, for 12 weeks. | Physical performance: pulmonary function |
| Huang RC 2011 | RCT | Chinese Mainland | Type 2 diabetes | The style of State General Administration of Sport of Chinese Mainland in 2003, 60 minutes per time, 5 times per week, for 24 weeks. | Laboratory tests: FPG, HbA1c, blood lipid |
| Li L 2014 | RCT | Chinese Mainland | Type 2 diabetes | Unspecified style, 30 minutes per time, 5 times per week, for 4 weeks. | Symptom: PSQI; Laboratory tests: FPG, HbA1c, blood lipid |
| Du HH 2017 | RCT | Chinese Mainland | Low back pain | The style of State General Administration of Sport of Chinese Mainland in 2003, 20 minutes per session, twice per day, 5 times per week, for 8 weeks. | Symptom: VAS |
| He XQ 2017 | RCT | Chinese Mainland | Type 2 diabetes | The style of State General Administration of Sport of Chinese Mainland in 2003, supervised by qualified instructor, 40 minutes per time, 5 times per week, for 3 weeks. | Laboratory tests: FPG, HbA1c; QOL |
| Huang YF 2016 | RCT | Chinese Mainland | Osteopenia | Unspecified style, 15 minutes per time, 5 times per week, for 24 weeks. | Physical performance: BMD |
| Niu H 2019 | RCT | Chinese Mainland | Stroke | Unspecified style, 30 minutes per time, 5 times per week, for 6 weeks. | Physical performance: cognitive function; QOL |
| Zhang XY 2017 | RCT | Chinese Mainland | Heart disease | Unspecified style, supervised by qualified instructor, 30 minutes per time, 5 times per week, for 6 weeks. | Symptom; Physical performance: cardiac function; QOL: SF-36 |
| Lin YN 2007 | RCT | Chinese Mainland | Type 2 diabetes | The style of State General Administration of Sport of Chinese Mainland in 2003, supervised by qualified instructor, 20 to 30 minutes per time, 5 times per week, for 12 weeks. | Laboratory tests: FPG, HbA1c; QOL; Psychological |
| Liang N 2017 | RCT | Chinese Mainland | Type 2 diabetes | Unspecified style, 60 minutes per time, 5 times per week, for 16 weeks. | Symptom |
| Wang F 2009 | RCT | Chinese Mainland | Type 2 diabetes | Unspecified style, for 20 weeks. | Symptom: PSQI |
| Wang F 2008 | RCT | Chinese Mainland | Type 2 diabetes | The style of State General Administration of Sport of Chinese Mainland in 2003, supervised by qualified instructor, 60 minutes per time, 5 times per week, for 16 weeks. | Symptom: PSQI; Laboratory tests: FPG, HbA1c; Psychological: anxiety, depression |
| Lin YN 2008 | RCT | Chinese Mainland | Type 2 diabetes | Unspecified style, 60 minutes per time, 5 times per week, for 16 weeks. | Symptom |
| Lin YN 2009 | RCT | Chinese Mainland | Type 2 diabetes | Unspecified style, 60 minutes per time, 5 times per week, for 16 weeks. | Laboratory tests: FPG, HbA1c; QOL |
| Qin G 2012 | RCT | Chinese Mainland | Health promotion | Unspecified style, 60 minutes per time, 5 times per week, for 20 weeks. | Laboratory tests: cardiovascular function |
| Ma SH 2011 | RCT | Chinese Mainland | Perimenopausal syndrome | The style of State General Administration of Sport of Chinese Mainland in 2003, supervised by qualified instructor, 50 minutes per time, 5 times per week, for 12 weeks. | Symptom |
| Dong LJ 2018 | RCT | Chinese Mainland | COPD | Other style, supervised by qualified instructor, 60 minutes per time, 5 times per week, for 12 weeks. | Physical performance: pulmonary function; QOL; Psychological: anxiety, depression |
| Su YS 2014 | RCT | Chinese Mainland | Health promotion | The style of State General Administration of Sport of Chinese Mainland in 2003, supervised by qualified instructor, 60 minutes per time, 5 times per week, for 12 weeks. | Symptom: PSQI; QOL: WHOQOL-BREF; Psychological: anxiety, depression |
| Li DJ 2006 | RCT | Chinese Mainland | Cardiovascular diseases | Unspecified style, supervised by qualified instructor, 40 minutes per time, 5 times per week, for 10 weeks. | Physical performance |
| Wang YZ 2019 | RCT | Chinese Mainland | Neck pain | The style of State General Administration of Sport of Chinese Mainland in 2003, supervised by qualified instructor, 60 minutes per time, 5 times per week, for 4 weeks. | Symptom: NDI |
| Shi LY 2013 | RCT | Chinese Mainland | Coronary heart disease | Unspecified style, 30 to 40 minutes per time, 5 times per week, for 24 weeks. | Laboratory tests: electrocardiogram |
| Wang L 2018 | RCT | Chinese Mainland | Stroke | Unspecified style, 40 minutes per time, 5 times per week, for 12 weeks. | Symptom; QOL |
| Guo JC 2016 | RCT | Chinese Mainland | COPD | Unspecified style, supervised by qualified instructor, 30 minutes per time, 4 times per week, for 24 weeks. | Symptom; Laboratory tests: pulmonary function |
| Han R 2015 | RCT | Chinese Mainland | Lung cancer | Unspecified style, supervised by qualified instructor, 30 minutes per session, twice per day, 5 times per week, for 12 weeks. | Symptom: VAS; Physical performance: pulmonary function, BMI; QOL |
| Lin XM 2015 | RCT | Chinese Mainland | Hypertension | Other style, 30 minutes per time, 7 times per week. | Symptom; Physical performance: blood pressure |
| Chen QY 2013 | RCT | Chinese Mainland | Hypertension | The style of State General Administration of Sport of Chinese Mainland in 2003, supervised by qualified instructor, 30 minutes per time, 5 times per week, for 12 weeks. | Laboratory tests; Physical performance: blood pressure |
| Wang H 2017 | RCT | Chinese Mainland | Cancer | Unspecified style, supervised by qualified instructor, 60 minutes per session, twice per day, 5 times per week, for 4 weeks. | Symptom: PSQI; Psychological |
| Chen XM 2014 | RCT | Chinese Mainland | Health promotion | Unspecified style, 60 minutes per time, 4 times per week, for 12 weeks. | Physical performance: heart rate |
| Ding M 2008 | RCT | Chinese Mainland | Health promotion | Unspecified style, 40 minutes per time, 3 times per week, for 6 weeks. | Laboratory tests: urine protein; Physical performance: pulmonary function, strength, heart rate |
| Zhang YG 2018 | RCT | Chinese Mainland | Coronary heart disease | Unspecified style, supervised by qualified instructor, 30 minutes per time, 5 times per week, for 12 weeks. | Symptom; Physical performance: pulmonary function; Psychological: anxiety, depression |
| Cai B 2017 | RCT | Chinese Mainland | Neck pain | Unspecified style, supervised by qualified instructor, 30 to 60 minutes per session, twice per day, 5 times per week, for 4 weeks. | Symptom |
| Zhang SW 2013 | RCT | Chinese Mainland | Low back pain | The style of State General Administration of Sport of Chinese Mainland in 2003, 90 minutes per time, 5 times per week, for 144 weeks. | Symptom: JOA |
| Li YJ 2016 | RCT | Chinese Mainland | Type 2 diabetes | Deng Tietao style, supervised by qualified instructor, 30 minutes per session, 1 to 2 sessions per day, 5 times per week, for 12 weeks. | Symptom; Laboratory tests: FPG, HbA1c, blood lipid; Physical performance: BMI |
| Wu XC 2018 | RCT | Chinese Mainland | Type 2 diabetes | Deng Tietao style, supervised by qualified instructor, 30 minutes per time, 4 times per week, for 8 weeks. | Laboratory tests: FPG, HbA1c, blood lipid; Physical performance: BMI; Psychological: anxiety, depression |
| Wu XC 2019 | RCT | Chinese Mainland | Type 2 diabetes | Deng Tietao style, supervised by qualified instructor, 30 minutes per time, 4 times per week, for 8 weeks. | Laboratory tests: FPG, HbA1c, blood lipid; Physical performance: BMI; Psychological: anxiety, depression |
| Guo LH 2012 | RCT | Chinese Mainland | Heart disease | Deng Tietao style, 20 minutes per session, twice per day, 5 times per week, for 12 weeks. | Symptom; QOL: SF-36 |
| Chen CY 2019 | RCT | Chinese Mainland | Hypertension | Unspecified style, 15 to 20 minutes per session, twice per day, 5 times per week, for 12 weeks. | Physical performance: blood pressure |
| Liang W 2014 | RCT | Chinese Mainland | Neck pain | Unspecified style, 20 minutes per time, 3 times per week, for 12 weeks. | Symptom |
| Zhang XF 2011 | RCT | Chinese Mainland | Neck pain | Unspecified style. | Symptom |
| Chen HB 2019 | RCT | Chinese Mainland | Neck pain | Unspecified style, 2 sessions per day, 5 times per week, for 2 weeks. | Symptom: VAS |
| Zeng N 2019 | RCT | Chinese Mainland | Type 2 diabetes | Unspecified style, supervised by qualified instructor, 30 minutes per session, twice per day, 5 times per week, for 12 weeks. | Laboratory tests: FPG, HbA1c, blood lipid |
| Li MN 2018 | RCT | Chinese Mainland | Hypertension | Unspecified style, 20 minutes per session, twice per day, 5 times per week. | Symptom; Physical performance: blood pressure |
| Lai YL 2016 | RCT | Chinese Mainland | Hypertension | Unspecified style, supervised by qualified instructor, 25 minutes per session, twice per day, 5 times per week, for 12 weeks. | Symptom; Physical performance: blood pressure |
| Liu YJ 2015 | RCT | Chinese Mainland | Low back pain | The style of State General Administration of Sport of Chinese Mainland in 2003, 15 minutes per time, 5 times per week, for 4 weeks. | Symptom: VAS, ODI |
| Lin GY 2018 | RCT | Chinese Mainland | Schizophrenia | Unspecified style, 30 minutes per time, 5 times per week, for 12 weeks. | Symptom: PSQI; QOL |
| Chen ZL 2018 | RCT | Chinese Mainland | Cognitive impairment | The style of State General Administration of Sport of Chinese Mainland in 2003, 3 times per week, for 36 weeks. | Physical performance: memory function |
| Huang XL 2016 | RCT | Chinese Mainland | Lung cancer | Unspecified style, 30 minutes per session, twice per day, 5 times per week, for 12 weeks. | Symptom; Physical performance: pulmonary function; QOL |
| Gao TT 2019 | RCT | Chinese Mainland | Type 2 diabetes | Homemade style, 30 minutes per time, 4 times per week, for 12 weeks. | Laboratory tests: FPG, HbA1c, blood lipid; QOL: WHOQOL-BREF |
| Yan JM 2018 | RCT | Chinese Mainland | Fracture | Homemade style, supervised by qualified instructor, 15 to 20 minutes per session, 3 sessions per day, 5 times per week, for 2 weeks. | Symptom |
| Zhao C 2017 | RCT | Chinese Mainland | Heart disease | Homemade style, supervised by qualified instructor, 20 minutes per time, 5 times per week, for 8 weeks. | Symptom; Laboratory tests: electrocardiogram; Physical performance: cardiopulmonary function; QOL |
| Qu HJ 2012 | RCT | Chinese Mainland | Health promotion | Unspecified style, supervised by qualified instructor, 30 to 40 minutes per time, 4 times per week, for 16 weeks. | Symptom; Laboratory tests |
| Chen KM 2017 | RCT | Chinese Mainland | Insomnia | The style of State General Administration of Sport of Chinese Mainland in 2003, supervised by qualified instructor, 20 minutes per session, twice per day, 5 times per week, for 4 weeks. | Symptom: PSQI |
| Zhang TT 2019 | RCT | Chinese Mainland | COPD | The style of State General Administration of Sport of Chinese Mainland in 2003, supervised by qualified instructor, 30 minutes per time, 5 times per week, for 24 weeks. | Laboratory tests; Physical performance: pulmonary function |
| Zhufu Y 2018 | RCT | Chinese Mainland | COPD | The style of State General Administration of Sport of Chinese Mainland in 2003, supervised by qualified instructor, 4 times per week, for 24 weeks. | Laboratory tests; Physical performance: pulmonary function |
| Wu LF 2017 | RCT | Chinese Mainland | Low back pain | Unspecified style, supervised by qualified instructor, 60 minutes per time, 3 times per week, for 8 weeks. | Laboratory tests: surface electromyography; Symptom |
| Wang SN 2018 | RCT | Chinese Mainland | COPD | Other style, supervised by qualified instructor, 10 to 15 minutes per time, 3 times per week, for 8 weeks. | Laboratory tests; Physical performance: pulmonary function |
| Zhang LM 2016 | RCT | Chinese Mainland | Perimenopausal syndrome | Unspecified style, supervised by qualified instructor, 45 minutes per time, 5 times per week, for 12 weeks. | Symptom: PSQI; Psychological: anxiety, depression |
| Zhang CB 2017 | RCT | Chinese Mainland | Osteoporosis | The style of State General Administration of Sport of Chinese Mainland in 2003, supervised by qualified instructor, 2 sessions per day, 5 times per week, for 48 weeks. | Laboratory tests |
| Hu QC 2019 | RCT | Chinese Mainland | Health promotion | The style of State General Administration of Sport of Chinese Mainland in 2003, 15 minutes per time, 5 times per week, for 10 weeks. | Laboratory tests |
| Sa ZY 2018 | RCT | Chinese Mainland | Health promotion | Unspecified style, supervised by qualified instructor, 60 minutes per time, 5 times per week, for 12 weeks. | Laboratory tests |
| Sa ZY 2017 | RCT | Chinese Mainland | Health promotion | The style of State General Administration of Sport of Chinese Mainland in 2003, supervised by qualified instructor, 60 minutes per time, 5 times per week, for 12 weeks. | Laboratory tests |
| Li MY 2017 | RCT | Chinese Mainland | Cognitive impairment | The style of State General Administration of Sport of Chinese Mainland in 2003, supervised by qualified instructor, 60 minutes per time, 3 times per week, for 24 weeks. | Physical performance: cognition function, attention |
| Ye BZ 2019 | RCT | Chinese Mainland | Cognitive impairment | The style of State General Administration of Sport of Chinese Mainland in 2003, supervised by qualified instructor, 60 minutes per time, 3 times per week, for 24 weeks. | Laboratory tests; Physical performance: cognition function |
| Chen H 2016 | RCT | Chinese Mainland | Low back pain | Unspecified style, 20 minutes per time, 5 times per week, for 3 weeks. | Symptom: JOA; Laboratory tests |
| Shao LL 2019 | RCT | Chinese Mainland | Neck pan, Low back pain | The style of State General Administration of Sport of Chinese Mainland in 2003, supervised by qualified instructor, 30 minutes per session, twice per day, 5 times per week, for 8 weeks. | Symptom; QOL |
| Lin Q 2017 | RCT | Chinese Mainland | Hypertension | The style of State General Administration of Sport of Chinese Mainland in 2003, supervised by qualified instructor, 30 to 40 minutes per time, 5 times per week, for 24 weeks. | Laboratory tests; Physical performance: blood pressure, heart rate |
| Gu F 2018 | RCT | Chinese Mainland | Coronary heart disease | The style of State General Administration of Sport of Chinese Mainland in 2003, 3 to 5 times per week, for 12 weeks. | Symptom |
| Xuan SS 2016 | RCT | Chinese Mainland | Neck pain | Unspecified style, 40 minutes per time, 5 times per week, for 3 weeks. | Symptom |
| Wu LS 2016 | RCT | Chinese Mainland | Hyperlipidemia | Unspecified style, 15 minutes per session, twice per day, 5 times per week, for 8 weeks. | Laboratory tests: blood lipid |
| Kuan XW 2019 | RCT | Chinese Mainland | Osteoporosis | Unspecified style, 60 minutes per session, twice per day, 5 times per week, for 12 weeks. | Symptom: VAS; Physical performance: BMD; QOL |
| Liang L 2014 | RCT | Chinese Mainland | Low back pain | Unspecified style, 60 minutes per time, 5 times per week, for 12 weeks. | Symptom: range of motion |
| Zhang ZK 2016 | RCT | Chinese Mainland | Glaucoma | Unspecified style, 30 minutes per time, 5 times per week, for 12 weeks. | Symptom; Physical performance; QOL; Psychological: anxiety, depression |
| Lai QY 2018 | RCT | Chinese Mainland | Health promotion | The style of State General Administration of Sport of Chinese Mainland in 2003, 60 minutes per time, 5 times per week, for 12 weeks. | Physical performance |
| Wang XQ 2014 | RCT | Chinese Mainland | Mental illness | Unspecified style, supervised by qualified instructor, 30 minutes per time, 5 times per week, for 24 weeks. | Laboratory tests: FPG, blood lipid; Physical performance: BMI |
| Li M 2018 | RCT | Chinese Mainland | COPD | The style of State General Administration of Sport of Chinese Mainland in 2003, supervised by qualified instructor, 60 minutes per time, 4 times per week, for 24 weeks. | Symptom; Physical performance; QOL; Psychological: anxiety, depression |
| He LP 2017 | RCT | Chinese Mainland | Health promotion | Unspecified style, 60 minutes per time, 4 times per week, for 12 weeks. | Physical performance |
| Sun YP 2014 | RCT | Chinese Mainland | COPD | The style of State General Administration of Sport of Chinese Mainland in 2003, supervised by qualified instructor, 60 minutes per time, 5 times per week, for 48 weeks. | Symptom; Physical performance: pulmonary function |
| Lin YF 2013 | RCT | Chinese Mainland | Type 2 diabetes | Unspecified style, 45 minutes per session, twice per day, 5 times per week, for 24 weeks. | Laboratory tests: FPG, HbA1c, blood lipid |
| Liu Q 2014 | RCT | Chinese Mainland | Chronic | Unspecified style, 30 minutes per time, 5 times per week, for 24 weeks. | Symptom |
| Liu JR 2011 | RCT | Chinese Mainland | Health promotion | Unspecified style, supervised by qualified instructor, 30 minutes per time, 5 times per week, for 12 weeks. | Laboratory tests: blood glucose |
| Liu JR 2006 | RCT | Chinese Mainland | Hyperlipidemia | Unspecified style, 30 minutes per session, twice per day, 5 times per week, for 12 weeks. | Laboratory tests: blood glucose |
| Wu JL 2013 | RCT | Chinese Mainland | Health promotion | The style of State General Administration of Sport of Chinese Mainland in 2003, supervised by qualified instructor, 30 minutes per session, twice per day, 5 times per week, for 12 weeks. | Physical performance: heart rate |
| Li MY 2015 | RCT | Chinese Mainland | Health promotion | The style of State General Administration of Sport of Chinese Mainland in 2003, supervised by qualified instructor, 90 minutes per time, 5 times per week, for 12 weeks. | Physical performance: pulmonary function, balance |
| Hu WW 2017 | RCT | Chinese Mainland | Health promotion | The style of State General Administration of Sport of Chinese Mainland in 2003, 60 minutes per time, 5 times per week, for 12 weeks. | Physical performance: pulmonary function, BMI, blood pressure; Psychological: SCL-90 |
| Wan Y 2011 | RCT | Chinese Mainland | Health promotion | The style of State General Administration of Sport of Chinese Mainland in 2003, 90 minutes per time, 5 times per week, for 14 weeks. | Psychological |
| Mo GN 2015 | RCT | Chinese Mainland | Health promotion | Unspecified style, 60 minutes per time, 3 times per week, for 8 weeks. | Psychological |
| Li XH 2009 | RCT | Chinese Mainland | Type 2 diabetes | The style of State General Administration of Sport of Chinese Mainland in 2003, supervised by qualified instructor, 60 minutes per time, 3 times per week, for 24 weeks. | Laboratory tests: blood glucose, blood lipid, vascular function |
| Zhang XQ 2008 | RCT | Chinese Mainland | Metabolic syndrome | Unspecified style, 60 minutes per time, 5 times per week, for 24 weeks. | Laboratory tests: blood glucose, blood lipid; Physical performance: heart rate, blood pressure |
| Liu HF 2007 | RCT | Chinese Mainland | Health promotion | Unspecified style, supervised by qualified instructor, 90 minutes per time, 5 times per week, for 12 weeks. | Psychological: SCL-90 |
| Yu ZS 2017 | RCT | Chinese Mainland | Obesity | Unspecified style, 60 minutes per time, 5 times per week, for 16 weeks. | Laboratory tests: blood glucose, blood lipid; Physical performance |
| Tang QH 2008 | RCT | Chinese Mainland | Health promotion | Unspecified style, 45 to 60 minutes per time, 5 to 7 times per week, for 48 weeks. | Physical performance |
| Ke XJ 2019 | RCT | Chinese Mainland | Neck pain | The style of State General Administration of Sport of Chinese Mainland in 2003, supervised by qualified instructor, 45 to 60 minutes per time, 5 times per week, for 10 weeks. | Symptom: VAS, NDI; Psychological: anxiety, depression |
| Bai SG 2014 | RCT | Chinese Mainland | Health promotion | Unspecified style, 90 minutes per time, 5 times per week, for 12 weeks. | Physical performance |
| Liu ST 2015 | RCT | Chinese Mainland | Health promotion | Unspecified style, 30 minutes per session, twice per day, 5 times per week, for 12 weeks. | Physical performance: balance |
| Xue WS 2013 | RCT | Chinese Mainland | Health promotion | Unspecified style, supervised by qualified instructor, 40 minutes per time, 5 times per week, for 24 weeks. | Physical performance: cardiopulmonary function |
| Li RW 2018 | RCT | Chinese Mainland | Health promotion | Unspecified style, 90 minutes per time, 4 times per week, for 8 weeks. | Psychological: SCL-90 |
| Sun G 2004 | RCT | Chinese Mainland | Health promotion | Unspecified style, supervised by qualified instructor, 30 to 40 minutes per time, 5 times per week, for 12 weeks. | Laboratory tests: blood lipid; Physical performance: blood pressure, pulmonary function, strength, balance |
| Mo GN 2013 | RCT | Chinese Mainland | Health promotion | Unspecified style, 60 minutes per time, 3 times per week, for 24 weeks. | Psychological: SCL-90 |
| Ren LS 2015 | RCT | Chinese Mainland | Health promotion | The style of State General Administration of Sport of Chinese Mainland in 2003, 30 minutes per time, 3 times per week, for 8 weeks. | Psychological |
| Niu W 2011 | RCT | Chinese Mainland | Health promotion | Unspecified style, 90 minutes per time, 3 times per week, for 12 weeks. | Physical performance |
| Liu Y 2012 | RCT | Chinese Mainland | Type 2 diabetes | The style of State General Administration of Sport of Chinese Mainland in 2003, supervised by qualified instructor, 40 minutes per time, 3 to 5 times per week, for 12 weeks. | Laboratory tests: HbA1c; QOL; Psychological: depression |
| Huo R 2010 | RCT | Chinese Mainland | Type 2 diabetes | The style of State General Administration of Sport of Chinese Mainland in 2003, supervised by qualified instructor, 30 minutes per time, 5 times per week, for 12 weeks. | Laboratory tests: HbA1c; QOL; Psychological: depression |
| Liu HF 2007 | RCT | Chinese Mainland | Health promotion | Unspecified style, supervised by qualified instructor, 90 minutes per time, 5 times per week, for 12 weeks. | Psychological |
| Liu HF 2008 | RCT | Chinese Mainland | Health promotion | The style of State General Administration of Sport of Chinese Mainland in 2003, supervised by qualified instructor, 90 minutes per time, 5 times per week, for 12 weeks. | Psychological: SCL-90 |
| Cui YS 2018 | RCT | Chinese Mainland | Stroke | Unspecified style, 45 minutes per time, 5 times per week, for 8 weeks. | Physical performance: balance |
| Liu HF 2008 | RCT | Chinese Mainland | Health promotion | Unspecified style, supervised by qualified instructor, 90 minutes per time, 5 times per week, for 12 weeks. | Psychological: SCL-90 |
| Tao JJ 2019 | RCT | Chinese Mainland | Neck pain | The style of State General Administration of Sport of Chinese Mainland in 2003, supervised by qualified instructor, 24 minutes per time, 3 times per week, for 12 weeks. | Symptom: VAS, range of motion |
| Pei Y 2018 | RCT | Chinese Mainland | Health promotion | Unspecified style, 60 minutes per time, 5 times per week, for 12 weeks. | Physical performance; Psychological: anxiety, depression |
| Zhang LF 2018 | RCT | Chinese Mainland | Type 2 diabetes | Unspecified style, supervised by qualified instructor, 60 minutes per time, 5 times per week. | Laboratory tests: blood glucose, blood lipid, hemorheology |
| Sun MH 2018 | RCT | Chinese Mainland | Type 2 diabetes | The style of State General Administration of Sport of Chinese Mainland in 2003, 60 minutes per time, 4 times per week, for 12 weeks. | Laboratory tests: FPG, HbA1c; Physical performance: blood pressure |
| Wang YH 2007 | RCT | Chinese Mainland | Type 2 diabetes | The style of State General Administration of Sport of Chinese Mainland in 2003, supervised by qualified instructor, 60 minutes per time, once per week, for 24 weeks. | Laboratory tests: blood glucose, blood lipid, hemorheology |
| Wang C 2009 | RCT | Chinese Mainland | Frozen shoulder | The style of State General Administration of Sport of Chinese Mainland in 2003, 30 minutes per session, twice per day, once per week, for 4 weeks. | Symptom: VAS, range of motion |
| Yu P 2019 | RCT | Chinese Mainland | COPD | Unspecified style, supervised by qualified instructor, 30 minutes per time, 3 times per week, for 24 weeks. | Physical performance: pulmonary function |
| Zhang Q 2018 | RCT | Chinese Mainland | Neck pain | Unspecified style, 30 minutes per session, twice per day, 5 times per week, for 4 weeks. | Symptom; Physical performance |
| Yang MC 2012 | RCT | Chinese Mainland | Type 2 diabetes | Unspecified style, supervised by qualified instructor, 60 minutes per time, 4 times per week, for 24 weeks. | Laboratory tests: FPG, HbA1c, blood lipid |
| Zhang X 2011 | RCT | Chinese Mainland | Type 2 diabetes | Unspecified style, 60 minutes per time, 5 times per week, for 24 weeks. | Laboratory tests: FPG, HbA1c, blood lipid; Psychological: anxiety |
| Zhou T 2014 | RCT | Chinese Mainland | Type 2 diabetes | Unspecified style, 30 minutes per session, twice per day, 5 times per week, for 12 weeks. | Laboratory tests: FPG, HbA1c; Psychological: depression |
| Zhao K 2016 | RCT | Chinese Mainland | Impaired glucose tolerance | Unspecified style, 30 minutes per time, 5 times per week, for 48 weeks. | Psychological |
| Zeng SJ 2019 | RCT | Chinese Mainland | Health promotion | Unspecified style, 90 minutes per time, 5 times per week, for 12 weeks. | Physical performance: blood pressure, heart rate |
| Song XJ 2011 | RCT | Chinese Mainland | Health promotion | Unspecified style, 30 minutes per time, 3 times per week, for 18 weeks. | Psychological: SCL-90 |
| Yang L 2010 | RCT | Chinese Mainland | Health promotion | Unspecified style, supervised by qualified instructor, 90 minutes per time, 5 times per week, for 24 weeks. | Psychological |
| Han R 2016 | RCT | Chinese Mainland | Lung cancer | Unspecified style, supervised by qualified instructor, 30 minutes per session, twice per day, 5 times per week, for 12 weeks. | Physical performance: pulmonary function; QOL |
| Liu T 2018 | RCT | Chinese Mainland | Obesity | The style of State General Administration of Sport of Chinese Mainland in 2003, supervised by qualified instructor, 90 minutes per time, 6 times per week, for 24 weeks. | Laboratory tests: blood glucose, blood lipid; Physical performance |
| Miao FS 2009 | RCT | Chinese Mainland | Hyperlipidemia | Unspecified style, 50 to 60 minutes per time, 5 to 7 times per week, for 72 weeks. | Laboratory tests: blood lipid |
| Xue XJ 2018 | RCT | Chinese Mainland | Arrhythmia | The style of State General Administration of Sport of Chinese Mainland in 2003, 30 to 40 minutes per time, 3 to 5 times per week, for 8 weeks. | Physical performance: heart rate |
| Zhu XN 2014 | RCT | Chinese Mainland | Perimenopausal syndrome | Unspecified style, supervised by qualified instructor, 60 minutes per time, 5 times per week, for 24 weeks. | Laboratory tests: free radical metabolism |
| Wang DX 2009 | RCT | Chinese Mainland | Health promotion | The style of State General Administration of Sport of Chinese Mainland in 2003, supervised by qualified instructor, 60 minutes per time, 6 times per week, for 12 weeks. | Physical performance: blood pressure, heart rate |
| Hu GX 2014 | RCT | Chinese Mainland | Health promotion | Unspecified style, supervised by qualified instructor, 30 to 60 minutes per time, 4 to 7 times per week, for 24 weeks. | QOL: SF-36 |
| Wang R 2016 | RCT | Chinese Mainland | Heart disease | Unspecified style, for 12 weeks. | Symptom; QOL; Psychological: anxiety, depression |
| Liu SR 2014 | RCT | Chinese Mainland | COPD | Unspecified style, supervised by qualified instructor, 30 minutes per time, 7 times per week, for 12 weeks. | Physical performance: pulmonary function |
| Zuo RQ 2018 | RCT | Chinese Mainland | COPD | The style of State General Administration of Sport of Chinese Mainland in 2003, supervised by qualified instructor, 30 minutes per time, 5 times per week, for 8 weeks. | Physical performance: pulmonary function |
| Liu SR 2013 | RCT | Chinese Mainland | COPD | Unspecified style, supervised by qualified instructor, 30minutes per time, 5 times per week, for 12 weeks. | Laboratory tests; Physical performance: pulmonary function |
| Chen YF 2015 | RCT | Chinese Mainland | COPD | Unspecified style, supervised by qualified instructor, 30 minutes per time, 5 times per week, for 12 weeks. | Physical performance: pulmonary function |
| Liu SR 2012 | RCT | Chinese Mainland | COPD | Unspecified style, supervised by qualified instructor, 30 minutes per time, 5 times per week, for 12 weeks. | Physical performance: pulmonary function |
| Wang C 2008 | RCT | Chinese Mainland | Migraine | Unspecified style, supervised by qualified instructor, 10 minutes per session, twice per day, 5 times per week, for 12 weeks. | Laboratory tests: hemodynamics |
| Peng X 2016 | RCT | Chinese Mainland | Health promotion | The style of State General Administration of Sport of Chinese Mainland in 2003, 15 minutes per time, 3 times per week, for 6 weeks. | Laboratory tests; Physical performance |
| Wu L 2018 | RCT | Chinese Mainland | Breast cancer | Unspecified style, supervised by qualified instructor, 30 to 60 minutes per time, 2 to 5 times per week, for 12 weeks. | Symptom; Physical performance: pulmonary function; QOL |
| Fang CP 2014 | RCT | Chinese Mainland | Impaired glucose tolerance | Unspecified style, 30 minutes per time, 5 times per week, for 12 weeks. | Laboratory tests: FPG, HbA1c, blood lipid |
| Fang CP 2014 | RCT | Chinese Mainland | Impaired glucose tolerance | Unspecified style, 30 minutes per time, 5 times per week, for 12 weeks. | Psychological: SCL-90 |
| Chen KY 2008 | RCT | Chinese Mainland | Health promotion | Unspecified style, supervised by qualified instructor, 60 to 90 minutes per session, twice per day, 5 times per week, for 12 weeks. | Symptom |
| Ma X 2016 | RCT | Chinese Mainland | Health promotion | The style of State General Administration of Sport of Chinese Mainland in 2003, supervised by qualified instructor, 60 minutes per time, 3 times per week, for 24 weeks. | Physical performance: balance |
| Wang X 2017 | RCT | Chinese Mainland | Low back pain | The style of State General Administration of Sport of Chinese Mainland in 2003, 60 minutes per time, 3 to 4 times per week, for 12 weeks. | Symptom: VAS |
| Huang BJ 2017 | RCT | Chinese Mainland | COPD | Unspecified style, 30 minutes per time, 7 times per week, for 24 weeks. | Physical performance: pulmonary function; QOL |
| Wang DM 2019 | RCT | Chinese Mainland | COPD | Unspecified style, supervised by qualified instructor, 30 minutes per time, 7 times per week, for 12 weeks. | Physical performance: pulmonary function |
| Shen L 2016 | RCT | Chinese Mainland | Neck pain | Unspecified style, 60 minutes per time, 3 times per week, for 3 weeks. | Symptom; QOL |
| Shen L 2017 | RCT | Chinese Mainland | Neck pain | The style of Chinese Traditional Sport Health Preservation, 60 minutes per time, 3 times per week, for3 weeks. | Symptom |
| Chen J 2014 | RCT | Chinese Mainland | Health promotion | Unspecified style, 45 minutes per time, 3 times per week, for 12 weeks. | Laboratory tests: surface electromyography |
| Hou YS 2016 | RCT | Chinese Mainland | Health promotion | Unspecified style, 60 minutes per time, 5 times per week, for 12 weeks. | Physical performance: balance |
| Xue GW 2015 | RCT | Chinese Mainland | COPD | The style of State General Administration of Sport of Chinese Mainland in 2003, 30 minutes per time, 4 times per week, for 24 weeks. | Symptom; Physical performance: pulmonary function |
| Yang DL 2017 | RCT | Chinese Mainland | COPD | Unspecified style, supervised by qualified instructor, 30 minutes per time, 7 times per week, for 12 weeks. | Physical performance: pulmonary function; QOL |
| Yu YY 2019 | RCT | Chinese Mainland | COPD | Unspecified style, supervised by qualified instructor, 30 minutes per time, 7 times per week, for 12 weeks. | Physical performance: pulmonary function |
| Long YF 2011 | RCT | Chinese Mainland | Perimenopausal syndrome | The style of State General Administration of Sport of Chinese Mainland in 2003, supervised by qualified instructor, 30 minutes per session, twice per day, 6 times per week, for 12 weeks. | Symptom; Laboratory tests: sex hormones |
| Qi DF 2018 | RCT | Chinese Mainland | Neck pain | Unspecified style, 60 minutes per time, 3 times per week, for 10 weeks. | Symptom: VAS; Physical performance |
| Liu JR 2010 | RCT | Chinese Mainland | Health promotion | Unspecified style, supervised by qualified instructor, 30 minutes per session, twice per day, 5 times per week, for 12 weeks. | Laboratory tests: blood glucose |
| Shen LS 2017 | RCT | Chinese Mainland | Breast cancer | The style of State General Administration of Sport of Chinese Mainland in 2003, 30 minutes per time, 5 times per week, for 8 weeks. | Symptom: VAS, range of motion; QOL |
| Zhang JF 2013 | RCT | Chinese Mainland | Hypertension | Other style, 15 to 20 minutes per session, twice per day, 5 times per week, for 8 weeks. | QOL |
| Dong CL 2016 | RCT | Chinese Mainland | Hypertension | Homemade style, 15 to 20 minutes per session, twice per day, 5 times per week, for 3 weeks. | Physical performance: blood pressure |
| Wang Q 2010 | RCT | Chinese Mainland | Neck pain | Homemade style, 1 to 2 sessions per day, 5 times per week, for 12 weeks. | Symptom |
| Yang YY 2008 | RCT | Chinese Mainland | Neck pain | Homemade style, 1 to 2 sessions per day, 5 times per week, for 12 weeks. | Symptom |
| Chen SS 2017 | RCT | Chinese Mainland | Heart failure | Unspecified style, 20 to 30 minutes per time, 3 to 4 times per week, for 12 weeks. | Laboratory tests: cardiac function; QOL |
| He X 2015 | RCT | Chinese Mainland | Hypertension | Other style, 30 minutes per time, 5 times per week, for 12 weeks. | Physical performance: blood pressure |
| Lin Q 2018 | RCT | Chinese Mainland | Stroke | Unspecified style, 30 minutes per time, 5 times per week. | Symptom; Physical performance; QOL; Psychological: anxiety |
| Ding Y 2014 | RCT | Chinese Mainland | Low back pain | Unspecified style, 40 minutes per time, 5 times per week, for 12 weeks. | Symptom: VAS, ODI, range of motion |
| Ma Y 2018 | RCT | Chinese Mainland | Health promotion | The style of State General Administration of Sport of Chinese Mainland in 2003, supervised by qualified instructor, 3 times per week, for 12 weeks. | Physical performance: balance |
| Wang N 2017 | RCT | Chinese Mainland | Perimenopausal syndrome | Unspecified style, 5 times per week, for 12 weeks. | Symptom |
| Xiao B 2003 | RCT | Chinese Mainland | Neck pain | Unspecified style, 30 to 40 minutes per session, twice per day, 5 times per week, for 4 weeks. | Laboratory tests; Symptom: VAS |
| Chen JP 2018 | RCT | Chinese Mainland | Health promotion | Unspecified style, supervised by qualified instructor, 20 minutes per time, 5 times per week, for 14 weeks. | Symptom; Psychological: anxiety |
| Sun G 2008 | RCT | Chinese Mainland | Health promotion | Unspecified style, supervised by qualified instructor, 40 to 50 minutes per time, 5 times per week, for 12 weeks. | Physical performance: intelligence |
| Jing JH 2013 | RCT | Chinese Mainland | Health promotion | Unspecified style, 60 minutes per time, 5 times per week, for 24 weeks. | Physical performance |
| Wang D 2018 | RCT | Chinese Mainland | Health promotion | Unspecified style, supervised by qualified instructor, 40 minutes per time, 5 times per week, for 12 weeks. | Physical performance |
| Zhao YY 2016 | RCT | Chinese Mainland | Fibromyalgia syndrome | Homemade style, 30 minutes per time, 5 times per week, for 12 weeks. | Symptom; Physical performance |
| Zhang F 2017 | RCT | Chinese Mainland | Depression | Unspecified style, supervised by qualified instructor, 30 minutes per session, twice per day, 5 times per week, for 6 weeks. | QOL; Psychological: anxiety |
| Lv YL 2013 | RCT | Chinese Mainland | Health promotion | Unspecified style, supervised by qualified instructor, 30 minutes per time, 5 times per week, for 24 weeks. | Physical performance |
| Qiu XH 2014 | RCT | Chinese Mainland | Health promotion | Unspecified style, 50 minutes per time, 5 times per week, for 18 weeks. | Physical performance |
| Zhang SQ 2012 | RCT | Chinese Mainland | Breast cancer | Unspecified style, supervised by qualified instructor, 15 to 20 minutes per time, 5 times per week, for 4 weeks. | Symptom |
| Li JJ 2019 | RCT | Chinese Mainland | Health promotion | The style of State General Administration of Sport of Chinese Mainland in 2003, supervised by qualified instructor, 70 minutes per time, 5 times per week, for 24 weeks. | Physical performance: BMD |
| Dong LJ 2019 | RCT | Chinese Mainland | COPD | Other style, supervised by qualified instructor, 30 minutes per time, 4 times per week, for 24 weeks. | Physical performance: pulmonary function, BMI; QOL |
| Liu MX 2017 | RCT | Chinese Mainland | Type 2 diabetes | Unspecified style, supervised by qualified instructor, 20 minutes per session, twice per day, 5 times per week, for 12 weeks. | QOL |
| Wei SM 2007 | RCT | Chinese Mainland | Health promotion | The style of State General Administration of Sport of Chinese Mainland in 2003, 60 minutes per time, 5 times per week, for 12 weeks. | Laboratory tests: blood lipid; Physical performance; Psychological: anxiety, depression |
| Guo X 2019 | RCT | Chinese Mainland | Heart disease | The style of State General Administration of Sport of Chinese Mainland in 2003, supervised by qualified instructor, 40 minutes per session, twice per day, 5 times per week, for 4 weeks. | Symptom; Laboratory tests; Physical performance; Psychological: anxiety |
| Zhang XD 2017 | RCT | Chinese Mainland | Heart disease | The style of State General Administration of Sport of Chinese Mainland in 2003, supervised by qualified instructor, 20 to 30 minutes per session, 3 sessions per day, 5 times per week, for 4 weeks. | Symptom; Laboratory tests; Physical performance; Psychological: anxiety |
| Li GB 2018 | RCT | Chinese Mainland | Heart disease | The style of State General Administration of Sport of Chinese Mainland in 2003, 20 to 30 minutes per session, twice per day, 5 times per week, for 8 weeks. | Symptom; Laboratory tests: HbA1c, blood lipid; Physical performance: cardiopulmonary function |
| Zhang DM 2017 | RCT | Chinese Mainland | Health promotion | Unspecified style, supervised by qualified instructor, 60 minutes per time, 5 times per week, for 4 weeks. | Physical performance: balance |
| Yang XH 2016 | RCT | Chinese Mainland | Breast hyperplasia | Unspecified style, 20 minutes per time, 5 times per week, for 12 weeks. | Symptom |
| Yu D 2019 | RCT | Chinese Mainland | Heart failure | Unspecified style, 30 minutes per time, 4 to 5 times per week, for 12 weeks. | Laboratory tests; Physical performance: cardiopulmonary function; QOL: SF-36 |
| Yang HX 2019 | RCT | Chinese Mainland | Stroke | The style of State General Administration of Sport of Chinese Mainland in 2003, 40 minutes per session, twice per day, 5 times per week, for 8 weeks. | Symptom; Laboratory tests: surface electromyography; Physical performance |
| Wu YH 2016 | RCT | Chinese Mainland | Coronary heart disease | Unspecified style, 60 minutes per time, 5 times per week, for 12 weeks. | Psychological: anxiety, depression |
| Wu XM 2009 | RCT | Chinese Mainland | Health promotion | The style of Chinese Traditional Sport Health Preservation, 90 minutes per time, 2 times per week, for 16 weeks. | Physical performance; QOL; Psychological: SCL-90 |
| Wang DW 2016 | RCT | Chinese Mainland | Type 2 diabetes | The style of State General Administration of Sport of Chinese Mainland in 2003, supervised by qualified instructor, 30 minutes per time, 5 times per week, for 24 weeks. | Laboratory tests: FPG, HbA1c, blood lipid; QOL |
| Zhou E 2019 | RCT | Chinese Mainland | Frozen shoulder | Unspecified style, 5 times per week, for 24 weeks. | Symptom |
| Ma SH 2011 | RCT | Chinese Mainland | Perimenopausal syndrome | The style of State General Administration of Sport of Chinese Mainland in 2003, supervised by qualified instructor, 45 minutes per time, 5 times per week, for 20 weeks. | Symptom; Psychological |
| Ye T 2018 | RCT | Chinese Mainland | Stroke | The style of State General Administration of Sport of Chinese Mainland in 2003, supervised by qualified instructor, 20 minutes per session, twice per day, 5 times per week, for 6 weeks. | Physical performance: motor, balance; QOL |
| Chen CF 2016 | RCT | Chinese Mainland | Health promotion | Unspecified style, 30 minutes per time, 5 times per week, for 24 weeks. | Physical performance: balance; QOL: WHOQOL-BREF; Psychological: fear of falling |
| Yang LJ 2018 | RCT | Chinese Mainland | Low back pain | The style of State General Administration of Sport of Chinese Mainland in 2003 | Symptom: VAS, JOA |
| Lin HR 2018 | RCT | Chinese Mainland | Low back pain | The style of State General Administration of Sport of Chinese Mainland in 2003, 30 minutes per session, twice per day, 3 times per week, for 2 weeks. | Symptom: VAS |
| Zhang BJ 2012 | RCT | Chinese Mainland | Low back pain | Unspecified style, 30 minutes per time, 5 times per week, for 4 weeks. | Symptom: VAS, ODI |
| Qiu YC 2018 | RCT | Chinese Mainland | Low back pain | Unspecified style, 5 times per week, for 3 weeks. | Symptom: VAS, JOA, ODI |
| Yang J 2019 | RCT | Chinese Mainland | Cognitive impairment | Unspecified style, supervised by qualified instructor, 40 minutes per time, 5 times per week, for 24 weeks. | Physical performance: cognitive function; QOL |
| He GJ 2016 | RCT | Chinese Mainland | Breast cancer | Unspecified style, supervised by qualified instructor, 15 to 20 minutes per session, twice per day, 5 times per week, for 12 weeks. | Symptom |
| Lin MJ 2013 | RCT | Chinese Mainland | Stroke | Unspecified style, supervised by qualified instructor, 15 minutes per time, 5 times per week, for 4 weeks. | Psychological: anxiety |
| An BC 2008 | RCT | Chinese Mainland | Knee osteoarthritis | Unspecified style, supervised by qualified instructor, 30 minutes per time, 5 times per week, for 8 weeks. | Symptom; Physical performance: strength; QOL: SF-36 |
| Ji YL 2014 | RCT | Chinese Mainland | Multiple sclerosis | Unspecified style, supervised by qualified instructor, 30 minutes per time, 5 times per week. | QOL |
| Shi ZC 2019 | RCT | Chinese Mainland | Health promotion | Unspecified style, 60 minutes per time, once per week, for 10 weeks. | Psychological: anxiety, depression |
| Zhang J 2016 | RCT | Chinese Mainland | Anxiety | The style of State General Administration of Sport of Chinese Mainland in 2003, 30 minutes per time, 5 times per week, for 12 weeks. | Psychological: anxiety |
| Wang LH 2017 | RCT | Chinese Mainland | Hyperlipidemia | Unspecified style, 60 minutes per time, 5 times per week, for 12 weeks. | Laboratory tests: blood lipid |
| Zhang XY 2018 | RCT | Chinese Mainland | Chronic fatigue syndrome | Other style, 60 minutes per session, twice per day, 5 times per week, for 12 weeks. | Laboratory tests; Symptom; Psychological: anxiety |
| Wang L 2018 | RCT | Chinese Mainland | COPD | Unspecified style, supervised by qualified instructor, 30 minutes per time, 5 times per week, for 12 weeks. | Physical performance: pulmonary function; QOL |
| Liu XY 2018 | RCT | Chinese Mainland | COPD | Unspecified style, supervised by qualified instructor, 30 minutes per time, 5 times per week. | Laboratory tests: pulmonary function; QOL; Psychological: anxiety, depression |
| Shang QQ 2014 | RCT | Chinese Mainland | Low back pain | The style of State General Administration of Sport of Chinese Mainland in 2003, 30 minutes per session, twice per day, 5 times per week. | Symptom: VAS, JOA |
| Huang XL 2017 | RCT | Chinese Mainland | Addiction | The style of State General Administration of Sport of Chinese Mainland in 2003, supervised by qualified instructor, 30 minutes per session, twice per day, 5 times per week, for 24 weeks. | Symptom |
| Gao FZ 2018 | RCT | Chinese Mainland | Insomnia | The style of State General Administration of Sport of Chinese Mainland in 2003, supervised by qualified instructor, 30 minutes per time, 5 times per week, for 8 weeks. | Symptom: PSQI; Laboratory tests; Physical performance: balance; Psychological |
| Huang XL 2015 | RCT | Chinese Mainland | Addiction | The style of State General Administration of Sport of Chinese Mainland in 2003, 30 minutes per session, twice per day, 5 times per week, for 20 weeks. | Psychological: anxiety |
| Xiao B 2010 | RCT | Chinese Mainland | Health promotion | Unspecified style, 30 minutes per time, 5 times per week, for 12 weeks. | Symptom; Laboratory tests: hemorheology; QOL: SF-36 |
| Chen CL 2014 | RCT | Chinese Mainland | Addiction | Unspecified style, 60 minutes per time, 5 times per week, for 12 weeks. | Laboratory tests: T lymphocyte |
| Lu XX 2010 | RCT | Chinese Mainland | Health promotion | Unspecified style, supervised by qualified instructor, 90 minutes per time, 3 times per week, for 12 weeks. | Psychological: anxiety, depression |
| Peng RD 2018 | RCT | Chinese Mainland | Type 2 diabetes | The style of State General Administration of Sport of Chinese Mainland in 2003, 2 sessions per day,5 times per week, for 48 weeks. | Laboratory tests; Physical performance: BMD |
| He JH 2011 | RCT | Chinese Mainland | Health promotion | Unspecified style, 45 minutes per time, 5 times per week, for 20 weeks. | Laboratory tests; Physical performance |
| Sun G 2008 | RCT | Chinese Mainland | Health promotion | Unspecified style, supervised by qualified instructor, 40 to 50 minutes per time, 5 times per week, for 12 weeks. | Laboratory tests: blood lipid; Physical performance |
| Wang ST 2007 | RCT | Chinese Mainland | Health promotion | The style of Beijing Sport University, supervised by qualified instructor, 60 minutes per time, 5 times per week, for 24 weeks. | QOL: SF-36 |
| Li L 2014 | RCT | Chinese Mainland | Low back pain | The style of State General Administration of Sport of Chinese Mainland in 2003, supervised by qualified instructor, 90 minutes per time, 5 times per week, for 4 weeks. | Symptom: VAS, JOA |
| Hu J 2018 | RCT | Chinese Mainland | Low back pain | Unspecified style, 4 times per week, for 3 weeks. | Symptom: JOA |
| Pan BQ 2013 | RCT | Chinese Mainland | Obesity | The style of State General Administration of Sport of Chinese Mainland in 2003, 30 minutes per session, twice per day, 5 times per week, for 12 weeks. | Physical performance: BMI |
| Chen P 2016 | RCT | Chinese Mainland | COPD | Unspecified style, supervised by qualified instructor, 30 minutes per time, 5 times per week, for 24 weeks. | Laboratory tests: blood gas analysis; Physical performance: pulmonary function; QOL |
| Chen Y 2019 | RCT | Chinese Mainland | Hypertension | Unspecified style, 12 minutes per time, 5 times per week, for 24 weeks. | QOL |
| Wang QJ 2018 | RCT | Chinese Mainland | Hypertension | Other style, supervised by qualified instructor, 60 minutes per time, 5 times per week, for 24 weeks. | Symptom: VAS; Physical performance: blood pressure |
| Yan T 2018 | RCT | Chinese Mainland | Coronary heart disease | Unspecified style, 30 minutes per session, twice per day, 5 times per week, for 12 weeks. | Physical performance: cardiopulmonary function; QOL |
| Dong CP 2012 | RCT | Chinese Mainland | Health promotion | Unspecified style, 30 minutes per time, 4 times per week, for 24 weeks. | Laboratory tests |
| Shi DL 2011 | RCT | Chinese Mainland | COPD | Unspecified style, 60 minutes per time, 5 times per week. | Laboratory tests |
| Wu JS 2019 | RCT | Chinese Mainland | Addiction | The style of State General Administration of Sport of Chinese Mainland in 2003, supervised by qualified instructor, 60 minutes per time, 5 times per week, for 48 weeks. | Psychological: anxiety, depression |
| Liu T 2015 | RCT | Chinese Mainland | Cognitive impairment | Unspecified style, supervised by qualified instructor, 60 minutes per time, 6 times per week, for 24 weeks. | Laboratory tests: cerebrospinal fluid; Physical performance: cognitive function |
| Sun JP 2016 | RCT | Chinese Mainland | Cognitive impairment | Unspecified style, supervised by qualified instructor, 60 minutes per time, 6 times per week, for 24 weeks. | Laboratory tests: cerebrospinal fluid; Physical performance: cognitive function |
| Cheng Y 2016 | RCT | Chinese Mainland | Hypertension | The style of State General Administration of Sport of Chinese Mainland in 2003, supervised by qualified instructor, 30 minutes per time, 3 times per week, for 12 weeks. | Physical performance: blood pressure |
| Ji XD 2012 | RCT | Chinese Mainland | Type 2 diabetes | The style of State General Administration of Sport of Chinese Mainland in 2003, supervised by qualified instructor, 45 minutes per time, 5 times per week, for 8 weeks. | Laboratory tests: FPG, HbA1c; Psychological: anxiety, depression |
| Wang N 2018 | RCT | Chinese Mainland | Chronic pyelonephritis | Unspecified style, supervised by qualified instructor, 30 minutes per time, 5 times per week, for 4 weeks. | Symptom |
| Wang J 2019 | RCT | Chinese Mainland | Coronary heart disease | Unspecified style, supervised by qualified instructor, 30 minutes per time, 3 times per week. | Psychological: anxiety, depression |
| Pan HY 2016 | RCT | Chinese Mainland | COPD | Unspecified style, 30 minutes per time, 5 times per week, for 24 weeks. | Physical performance: pulmonary function; QOL |
| Gu TT 2010 | RCT | Chinese Mainland | Low back pain | Unspecified style, 30 minutes per time, 5 times per week, for 4 weeks. | Symptom: VAS |
| Tian ZC 2019 | RCT | Chinese Mainland | Frozen shoulder | The style of State General Administration of Sport of Chinese Mainland in 2003, 30 minutes per time, 5 times per week, for 3 weeks. | Symptom; Psychological: SCL-90 |
| Li HB 2010 | RCT | Chinese Mainland | Hypertension | Unspecified style, 10 minutes per session, twice per day, 5 times per week, for 2 weeks. | Physical performance: blood pressure, heart rate |
| Lu W 2019 | RCT | Chinese Mainland | Neck pain | The style of State General Administration of Sport of Chinese Mainland in 2003, 30 minutes per time, 5 times per week, for 4 weeks. | Symptom; Laboratory tests |
| Zhang P 2019 | RCT | Chinese Mainland | Ulcerative colitis | Unspecified style, 30 minutes per time, 5 times per week, for 12 weeks. | QOL; Psychological: anxiety |
| Wu X 2012 | RCT | Chinese Mainland | Low back pain | Unspecified style, 30 minutes per time, 5 times per week, for 4 weeks. | Symptom: ODI; QOL |
| Dong YY 2018 | RCT | Chinese Mainland | Parkinson's Disease | Unspecified style, 20 minutes per session, twice per day, 5 times per week, for 12 weeks. | Physical performance: cognitive function; QOL |
| Lin JC 2018 | RCT | Chinese Mainland | Type 2 diabetes | The style of State General Administration of Sport of Chinese Mainland in 2003, supervised by qualified instructor, 30 to 60 minutes per time, 5 times per week, for 8 weeks. | Laboratory tests: FPG, HbA1c |
| Li SP 2015 | RCT | Chinese Mainland | Health promotion | The style of State General Administration of Sport of Chinese Mainland in 2003, supervised by qualified instructor, 45 minutes per time, 5 times per week, for 12 weeks. | Symptom; Laboratory tests: sex hormones |
| Wang JY 2015 | RCT | Chinese Mainland | COPD | The style of State General Administration of Sport of Chinese Mainland in 2003, supervised by qualified instructor, 5 times per week, for 48 weeks. | Physical performance: pulmonary function |
| Tan YH 2017 | RCT | Chinese Mainland | Psoriasis | Unspecified style, supervised by qualified instructor, 20 minutes per session, twice per day, 5 times per week, for 24 weeks. | Symptom; Physical performance: BMD |
| Yang M 2019 | RCT | Chinese Mainland | Type 2 diabetes | Unspecified style, 5 times per week, for 8 weeks. | Symptom; Laboratory tests: FPG, HbA1c |
| Du XL 2014 | RCT | Chinese Mainland | Osteoporosis | Unspecified style, supervised by qualified instructor, 2 sessions per day, 5 times per week, for 24 weeks. | Symptom; Physical performance: BMD |
| Chen J 2015 | RCT | Chinese Mainland | Osteoporosis | Unspecified style, supervised by qualified instructor, 2 sessions per day, 5 times per week. | Symptom |
| Tang C 2018 | RCT | Chinese Mainland | Inflammatory bowel disease | The style of State General Administration of Sport of Chinese Mainland in 2003, supervised by qualified instructor, 15 to 20 minutes per session, twice per day, 5 to 6 times per week, for 12 weeks. | Symptom; QOL |
| Liao SQ 2013 | RCT | Chinese Mainland | Metabolic syndrome | The style of State General Administration of Sport of Chinese Mainland in 2003, supervised by qualified instructor, 30 minutes per time, 5 times per week, for 24 weeks. | Laboratory tests: FPG, HbA1c, blood lipid; Physical performance: blood pressure, BMI |
| Chen LH 2014 | RCT | Chinese Mainland | Low back pain | Unspecified style, 15 to 30 minutes per time, 5 times per week. | Symptom |
| Han J 2017 | RCT | Chinese Mainland | Heart failure | The style of State General Administration of Sport of Chinese Mainland in 2003, supervised by qualified instructor, 15 to 30 minutes per time, 3 to 4 times per week, for 24 weeks. | Laboratory tests; Physical performance: cardiac function, BMI |
| Shi ZB 2017 | RCT | Chinese Mainland | Hypertension | Unspecified style, 30 minutes per time, 4 to 5 times per week, for 12 weeks. | Physical performance: blood pressure |
| Liu L 2019 | RCT | Chinese Mainland | Parkinson's Disease | Unspecified style, supervised by qualified instructor, 60 minutes per session, twice per day, 5 times per week, for 3 weeks. | QOL; Psychological: anxiety |
| Wu WL 2010 | RCT | Chinese Mainland | Hyperuricemia | Deng Tietao style, supervised by qualified instructor, 2 sessions per day, 5 times per week, for 24 weeks. | Laboratory tests: blood uric acid |
| Pan JL2012 | RCT | Chinese Mainland | Hypertension | Deng Tietao style, supervised by qualified instructor, 2 sessions per day, 5 times per week, for 48 weeks. | Laboratory tests: blood glucose, blood lipid; Physical performance: blood pressure |
| Zhang RR 2008 | RCT | Chinese Mainland | Type 2 diabetes | Unspecified style, supervised by qualified instructor, 60 minutes per time, 5 times per week, for 16 weeks. | Laboratory tests: FPG, HbA1c, blood lipid; QOL; Psychological: SCL-90 |
| Zheng YL 2016 | RCT | Chinese Mainland | Type 2 diabetes | Unspecified style, supervised by qualified instructor, 60 minutes per time, 5 times per week, for 16 weeks. | Laboratory tests: FPG, HbA1c; QOL |
| Lin YN 2009 | RCT | Chinese Mainland | Type 2 diabetes | The style of State General Administration of Sport of Chinese Mainland in 2003, supervised by qualified instructor, 60 minutes per time, 5 times per week, for 16 weeks. | Laboratory tests: FPG, HbA1c; Psychological: SCL-90 |
| Jiang Y 2019 | RCT | Chinese Mainland | Type 2 diabetes | Unspecified style, 20 minutes per session, twice per day, 5 times per week, for 12 weeks. | Laboratory tests: FPG, HbA1c, blood lipid; Psychological: anxiety, depression |
| Wang X 2012 | RCT | Chinese Mainland | Health promotion | The style of State General Administration of Sport of Chinese Mainland in 2003, supervised by qualified instructor, 20 minutes per session, twice per day, 5 times per week, for 20 weeks. | Symptom |
| Li K 2019 | RCT | Chinese Mainland | Low back pain | The style of State General Administration of Sport of Chinese Mainland in 2003, supervised by qualified instructor, 10 minutes per time, 5 times per week, for 4 weeks. | Symptom: VAS, ODI |
| Wang JM 2018 | RCT | Chinese Mainland | Heart disease | Unspecified style, supervised by qualified instructor, 40 minutes per session, twice per day, 3 to 5 times per week, for 24 weeks. | Symptom; Laboratory tests; Physical performance: cardiac function |
| Zhu ZG 2014 | RCT | Chinese Mainland | COPD | Unspecified style, supervised by qualified instructor, 30 minutes per session, twice per day, 5 times per week, for 24 weeks. | Laboratory tests; Physical performance: pulmonary function |
| Peng XY 2015 | RCT | Chinese Mainland | Osteoporosis | Unspecified style, supervised by qualified instructor, 30 minutes per time, 5 times per week, for 2 weeks. | Symptom: VAS |
| Zhu ZG 2016 | RCT | Chinese Mainland | COPD | Unspecified style, supervised by qualified instructor, 30 minutes per session, twice per day, 5 times per week, for 24 weeks. | Symptom; Physical performance: pulmonary function |
| Zhu ZG 2017 | RCT | Chinese Mainland | COPD | Unspecified style, supervised by qualified instructor, 40 minutes per session, twice per day, 5 times per week, for 24 weeks. | Laboratory tests |
| Peng JY 2017 | RCT | Chinese Mainland | Coronary heart disease | Unspecified style, supervised by qualified instructor, 30 minutes per time, 5 times per week, for 4 weeks. | QOL: SF-36 |
| Cai W 2010 | RCT | Chinese Mainland | Stroke | Unspecified style, 30 minutes per time, 4 to 5 times per week, for 12 weeks. | QOL: WHOQOL-BREF |
| Cai W 2011 | RCT | Chinese Mainland | Stroke | Unspecified style, 30 minutes per time, 4 to 5 times per week, for 12 weeks. | QOL |
| Lu HL 2019 | RCT | Chinese Mainland | Heart failure | Unspecified style, 30 minutes per session, twice per day, 5 times per week, for 48 weeks. | Symptom; Physical performance |
| Wang JJ 2019 | RCT | Chinese Mainland | Coronary heart disease | Unspecified style, supervised by qualified instructor, 30 minutes per time, 5 times per week, for 24 weeks. | Laboratory tests; QOL; Psychological: anxiety, depression |
| Liu AH 2018 | RCT | Chinese Mainland | Depression | The style of State General Administration of Sport of Chinese Mainland in 2003, supervised by qualified instructor, 40 minutes per time, 5 times per week, for 8 weeks. | Psychological: anxiety, depression |
| Zhao LM | SR | Chinese Mainland | Cognitive impairment | The style of State General Administration of Sport of Chinese Mainland in 2003; Unspecified style; 60 minutes per time, 3 times per week, for 24 weeks; 30 minutes per time, 5 times per week, for 12 weeks; 60 minutes per time, 6 times per week, for 24 weeks. | Laboratory tests: cerebrospinal fluid; Physical performance: cognitive function, attention; QOL: SF-36 |
| Cheng FK 2015 | SR | Hong Kong | Mental illness | Unspecified style; The style of State General Administration of Sport of Chinese Mainland in 2003; Other style; 30 to 40 minutes per time, 5 times per week, for 12 weeks; 30 minutes per time, 5 times per week, for 16 weeks; 30 minutes per time, 5 times per week, for 12 weeks; 60 minutes per time, 6 times per week, for 20 weeks; 90 minutes per time, 5 times per week, for 24 weeks; 60 minutes per time, 3 times per week, for 24 weeks; 15 to 20 minutes per session, twice per day, 5 times per week, for 8 weeks. | Laboratory tests: immunity; Symptom; QOL: SF-36; Psychological: anxiety, depression |
| Xiong X 2015 | SR | Chinese Mainland | Hypertension | Unspecified style; Homemade style; The style of State General Administration of Sport of Chinese Mainland in 2003; 40 minutes per time, 5 times per week, for 24 weeks; 45 minutes per session, twice per day, 5 times per week, for 24 weeks; 20 to 40 minutes per time, 3 to 5 times per week, for 12 weeks; 30 minutes per time, 5 times per week, for 12 weeks.; 30 minutes per time, 4 to 5 times per week, for 12 weeks. | Symptom; Laboratory tests: blood glucose, blood lipid; Physical performance: heart rate, blood pressure; QOL: SF-36 |
| Freire MD 2013 | SR | Brazil | Type 2 diabetes | Unspecified style; The style of State General Administration of Sport of Chinese Mainland in 2003; 30 minutes per time, 5 times per week, for 24 weeks; 60 minutes per time, 5 times per week, for 16 weeks; 60 minutes per time, 5 times per week, for 24 weeks; 20 minutes per time, 5 times per week, for 6 weeks; 30 minutes per session, twice per day, 5 times per week, for 12 weeks. | Symptom; Laboratory tests: FPG, HbA1c, blood lipid; Physical performance: BMI; Psychological: anxiety, depression |
| Sun HL 2016 | SR | Chinese Mainland | Coronary heart disease | The style of State General Administration of Sport of Chinese Mainland in 2003; Deng Tietao style; Homemade style; 40 minutes per time, 5 times per week, for 12 weeks; 20 minutes per session, twice per day, 5 times per week, for 12 weeks; 30 to 45 minutes per session, twice per day, 5 times per week, for 20 weeks. | Symptom; QOL; Psychological: anxiety, depression |
| Song G 2018 | SR+meta | Chinese Mainland | Type 2 diabetes | The style of State General Administration of Sport of Chinese Mainland in 2003; Unspecified style; 45 minutes per session, twice per day, 5 times per week, for 24 weeks; 60 minutes per time, 5 times per week, for 16 weeks; 60 minutes per time, 5 times per week, for 24 weeks; 30 minutes per time, 5 times per week, for 12 weeks; 40 minutes per time, 3 to 5 times per week, for 12 weeks; 30 minutes per time, 5 times per week, for 4 weeks; 30 minutes per time, 5 times per week, for 24 weeks; 30 minutes per session, twice per day, 5 times per week, for 12 weeks; 20 minutes per session, twice per day, 5 times per week, for 8 weeks; 20 minutes per time, 5 times per week, for 6 weeks; 3 session per day, 5 times per week, for 24 weeks; 60 minutes per time, 5 times per week, for 24 weeks. | Symptom: PSQI; Laboratory tests: FPG, HbA1c, blood lipid; Physical performance: BMI, pulmonary function; QOL; Psychological: anxiety, depression, SCL-90 |
| Jiang YH 2017 | SR+meta | Chinese Mainland | Insomnia | Homemade style; Unspecified style; The style of State General Administration of Sport of Chinese Mainland in 2003; 30 minutes per time, 3 times per week, for 12 weeks; 60 minutes per time, 5 times per week, for 16 weeks; 60 minutes per time, 5 times per week, for 12 weeks; 45 to 60 minutes per time, 5 times per week, for 10 weeks; 30 minutes per time, 5 times per week, for 4 weeks; 20 minutes per session, twice per day, 5 times per week, for 4 weeks; 45 minutes per time, 5 times per week, for 12 weeks. | Symptom: PSQI; Laboratory tests: blood glucose, blood lipid; Physical performance; Psychological: anxiety, depression, SCL-90 |
| Li H 2019 | SR+meta | Chinese Mainland | Low back pain | Unspecified style; The style of State General Administration of Sport of Chinese Mainland in 2003; 40 minutes per time, 5 times per week, for 12 weeks; 30 minutes per time, 5 times per week, for 4 weeks; 30 minutes per session, twice per day, 5 times per week, for 8 weeks; 30 minutes per time, 5 times per week, for 8 weeks; 15 minutes per session, twice per day, 5 times per week, for 4 weeks; 30 minutes per session, twice per day, 5 times per week, for 4 weeks. | Symptom: VAS, ODI, JOA, range of motion |
| Zhou L 2018 | SR+meta | Hong Kong | Stroke | The style of State General Administration of Sport of Chinese Mainland in 2003; Unspecified style; 20 minutes per session, twice per day, 5 times per week, for 6 weeks; 30 minutes per time, 4 to 5 times per week, for 12 weeks; 30 to 40 minutes per time, 5 times per week, for 12 weeks; 20 minutes per session, twice per day, 5 times per week, for 8 weeks. | Symptom; Physical performance: motor, balance; QOL: WHOQOL-BREF |
| Wen J 2017 | SR+meta | Chinese Mainland | Type 2 diabetes | Unspecified style; Deng Tietao style; The style of State General Administration of Sport of Chinese Mainland in 2003; Homemade style; 30 minutes per time, 5 times per week, for 24 weeks; 60 minutes per time, 5 times per week, for 16 weeks; 45 minutes per session, twice per day, 5 times per week, for 24 weeks; 30 minutes per session, 1 to 2 sessions per day, 5 times per week, for 12 weeks; 60 minutes per time, 5 times per week, for 24 weeks; 20 minutes per time, 5 times per week, for 6 weeks; 3 session per day, 5 times per week, for 24 weeks; 30 minutes per time, 5 times per week, for 12 weeks; 30 minutes per session, twice per day, 5 times per week, for 12 weeks. | Symptom; Laboratory tests: FPG, HbA1c, blood lipid; Physical performance: BMI; QOL; Psychological: anxiety, depression |
| Gai TT 2019 | SR+meta | Chinese Mainland | Coronary heart disease | The style of State General Administration of Sport of Chinese Mainland in 2003; Unspecified style; 45 minutes per time, twice per week, for 12 weeks; 60 minutes per time, 3 times per week, for 12 weeks; 60 minutes per time, 5 times per week, for 12 weeks; 30 minutes per time, 5 times per week, for 12 weeks; 40 minutes per time, 5 times per week, for 12 weeks. | Laboratory tests; Symptom; Physical performance: cardiopulmonary function; Physical performance; QOL; Psychological: anxiety, depression |
| Pan MX 2019 | SR+meta | Chinese Mainland | Hyperlipidemia | Unspecified style; The style of State General Administration of Sport of Chinese Mainland in 2003; 20 minutes per session, twice per day, 5 times per week, for 12 weeks; 60 minutes per time, 5 times per week, for 12 weeks; 20 minutes per time, 7 times per week, for 24 weeks; 50 to 60 minutes per time, 5 to 7 times per week, for 72 weeks; 30 minutes per session, twice per day, 5 times per week, for 12 weeks. | Laboratory tests: blood lipid, blood glucose; Physical performance: BMI |
| Zou L 2018 | SR+meta | Hong Kong | Mental illness | The style of State General Administration of Sport of Chinese Mainland in 2003; Homemade style; Unspecified style; supervised by qualified instructor, 90 minutes per time, 2 times per week, for 12 weeks; 60 minutes per time, 5 times per week, for 40 weeks; 30 minutes per time, 4 times per week, for 24 weeks; 45 minutes per time, 5 times per week, for 12 weeks; 60 minutes per time, 5 times per week, for 24 weeks; 30 minutes per time, 5 times per week, for 12 weeks. | Symptom: PSQI; QOL; Psychological: anxiety, depression |
| So WWY 2019 | SR+meta | Hong Kong | Depression | Unspecified style; 45 minutes per time, 3 times per week, for 12 weeks. | Laboratory tests; Physical performance: strength, flexibility; Psychological: depression |
| Xiong X 2015 | SR+meta | Chinese Mainland | Hypertension | Unspecified style; The style of State General Administration of Sport of Chinese Mainland in 2003; 30 minutes per session, twice per day, 5 times per week, for 12 weeks; 30 minutes per time, 5 times per week, for 12 weeks; 60 minutes per time, 7 times per week, for 24 weeks; 30 to 40 minutes per time, 5 to 7 times per week, for 72 weeks; 20 minutes per session, twice per day, 5 times per week, for 12 weeks. | Symptom; Physical performance: heart rate, blood pressure; QOL: SF-36 |
| Zou L 2017 | SR+meta | Chinese Mainland | Health promotion | The style of State General Administration of Sport of Chinese Mainland in 2003; The style of Chinese Health Qigong Association; Unspecified style; 90 minutes per time, 5 times per week, for 12 weeks; 40 minutes per time, 3 to 5 times per week, for 12 weeks; 30 to 60 minutes per time, 4 to 7 times per week, for 24 weeks; 30 to 40 minutes per time, 5 times per week, for 12 weeks; 30 minutes per session, twice per day, 5 times per week, for 8 weeks; 30 minutes per time, 5 times per week, for 4 weeks; 90 minutes per time, 2 times per week, for 12 weeks; 20 minutes per session, twice per day, 5 times per week, for 24 weeks; 50 minutes per time, 5 times per week, for 18 weeks; 40 to 50 minutes per time, 5 times per week, for 8 weeks. | Symptom: PSQI; Physical performance: balance, strength, pulmonary function, blood pressure; QOL; Psychological: anxiety, depression |
| Zou L 2018 | SR+meta | Chinese Mainland | Chronic diseases | The style of State General Administration of Sport of Chinese Mainland in 2003; The style of Chinese Health Qigong Association; Unspecified style; 60 minutes per time, 5 times per week, for 4 weeks; 40 minutes per time, 5 times per week, for 8 weeks; 60 minutes per time, 7 times per week, for 24 weeks; 40 minutes per time, 7 times per week, for 12 weeks; 30 minutes per session, twice per day, 5 times per week, for 8 weeks; 30 minutes per time, 5 times per week, for 12 weeks; 90 minutes per time, 2 times per week, for 12 weeks; 40 to 50 minutes per time, 5 times per week, for 8 weeks. | Symptom: VAS, JOA Physical performance; QOL |
| Tong H 2019 | SR+meta | Chinese Mainland | COPD | Unspecified style; The style of State General Administration of Sport of Chinese Mainland in 2003; 30 minutes per time, 4 times per week, for 24 weeks; 30 minutes per time, 4 times per week, for 36 weeks. | Symptom; Physical performance: pulmonary function; QOL: SF-36 |
| Yuan QL 2015 | SR+meta | Chinese Mainland | Neck pain; Low back pain | Unspecified style; The style of State General Administration of Sport of Chinese Mainland in 2003; 30 minutes per time, 5 times per week, for 12 weeks; 60 minutes per time, 5 times per week, for 8 weeks; 30 minutes per session, twice per day, 5 times per week, for 8 weeks; 15 minutes per session, twice per day, 5 times per week, for 4 weeks. | Symptom: pain and disability |
| Chan JSY 2019 | SR+meta | Chinese Mainland | Cognitive impairment | Unspecified style; The style of State General Administration of Sport of Chinese Mainland in 2003; 20 minutes per session, twice per day, 5 times per week, for 12 weeks; 60 minutes per time, 5 times per week, for 12 weeks; 40 minutes per time, 7 times per week, for 72 weeks. | Physical performance: attentional ability, memory function, cognitive function |
| Zeng ZP 2019 | SR+meta | Chinese Mainland | Knee osteoarthritis | Unspecified style, 30 minutes per time, 5 times per week, for 12 weeks; 30 to 45 minutes per time, 6 times per week, for 48 weeks. | Symptom: VAS, WOMAC; Physical performance |
| Lauche R 2017 | SR+meta | Australia | Stroke | The style of State General Administration of Sport of Chinese Mainland in 2003; Unspecified style; 30 minutes per session, twice per day, 5 times per week, for 8 weeks; 30 minutes per time, 4 to 5 times per week, for 12 weeks; 40 minutes per time, 5 times per week, for 12 weeks. | Laboratory tests: blood glucose, blood lipid; Physical performance: blood pressure; Safety |
| Farhang M 2019 | SR+meta | Chile | Cognitive impairment | Unspecified style; The style of State General Administration of Sport of Chinese Mainland in 2003; 60 minutes per time, 5 times per week, for 12 weeks; 45 minutes per time, 3 times per week, for 12 weeks; 60 minutes per time, 3 times per week, for 24 weeks. | Incidence: falls; Physical performance: cognitive function; Psychological: fear of falling; |
| Liu X 2015 | SR+meta | Chinese Mainland | Depression | Unspecified styl; The style of State General Administration of Sport of Chinese Mainland in 2003; 40 minutes per time, 7 times per week, for 12 weeks; 30 minutes per session, twice per day, 5 times per week, for 8 weeks; 30 minutes per time, 5 times per week, for 12 weeks; 90 minutes per time, 2 times per week, for 12 weeks. | Psychological: depression |
| Wayne PM 2018 | SR+meta | USA | Cancer | Unspecified style; The style of State General Administration of Sport of Chinese Mainland in 2003; 30 minutes per time, 3 times per week, for 12 weeks; 60 minutes per time, 5 times per week, for 12 weeks; 30 minutes per time, 5 times per week, for 4 weeks; 45 minutes per time, 5 times per week, for 12 weeks. | Symptom: fatigue, PSQI; QOL; Psychological: anxiety, depression |
| Zou Y 2019 | SR+meta | Chinese Mainland | Type 2 diabetes | The style of State General Administration of Sport of Chinese Mainland in 2003; Unspecified style; 30 minutes per time, 5 times per week, for 24 weeks; 40 minutes per time, 3 to 5 times per week, for 12 weeks; 60 minutes per time, 5 times per week, for 16 weeks; 60 minutes per time, 7 times per week, for 12 weeks; 45 minutes per time, 5 times per week, for 8 weeks; 30 minutes per session, twice per day, 5 times per week, for 12 weeks. | Laboratory tests: FPG, HbA1c; QOL; Psychological: anxiety, depression, SCL-90 |
| Chen XY 2018 | SR+meta | Chinese Mainland | Hypertension | The style of Chinese Health Qigong Association; The style of State General Administration of Sport of Chinese Mainland in 2003; Homemade style; Unspecified style; Other style; 40 minutes per time, 5 times per week, for 24 weeks; 45 minutes per session, twice per day, 5 times per week, for 24 weeks; 30 minutes per session, twice per day, 5 times per week, for 12 weeks; 20 minutes per session, twice per day, 5 times per week, for 24 weeks; 30 minutes per time, 5 times per week, for 12 weeks; 60 minutes per time, 5 times per week, for 12 weeks. | Physical performance: blood pressure |
| Li XY 2019 | SR+meta | Chinese Mainland | Osteoporosis | The style of Chinese Health Qigong Association; The style of State General Administration of Sport of Chinese Mainland in 2003; Unspecified style; 40 to 60 minutes per time, 5 times per week, for 12 weeks; 50 minutes per session, twice per day, 5 times per week, for 16 weeks; 15 minutes per session, twice per day, 5 times per week, for 48 weeks; 20 minutes per session, twice per day, 5 times per week, for 24 weeks; 30 minutes per time, 5 times per week, for 12 weeks; 60 minutes per time, 5 times per week, for 48 weeks. | Laboratory tests: bone glaprotein, alkaline phosphatase, Ca2^+^; Physical performance: bone density; QOL |
| Yang XL 2019 | SR+meta | Chinese Mainland | Coronary heart disease | The style of State General Administration of Sport of Chinese Mainland in 2003; Homemade style; Unspecified style; 40 minutes per session, twice per day, 3 to 5 times per week, for 24 weeks; 60 minutes per time, 3 times per week, for 12 weeks; 30 minutes per time, 5 times per week, for 12 weeks; 40 minutes per time, 6 times per week, for 4 weeks; 45 minutes per time, 5 times per week, for 12 weeks; 20 minutes per time, 5 times per week, for 8 weeks. | Laboratory tests: cardiac function; Physical performance: cardiac function |
| Wang Y 2019 | SR+meta | Chinese Mainland | Heart failure | The style of State General Administration of Sport of Chinese Mainland in 2003; The style of Chinese Traditional Sport Health Preservation; Unspecified style, 30 minutes per time, 5 times per week, for 12 weeks; 20 to 30 minutes per time, 3 to 4 times per week, for 12 weeks; 35 minutes per time, 3 times per week, for 12 weeks; 45 minutes per time, 5 times per week, for 12 weeks. | Laboratory tests: cardiac function; Physical performance: cardiopulmonary function; QOL; adverse reactions |
| Zheng QX 2017 | SR+meta | Chinese Mainland | Stroke | The style of State General Administration of Sport of Chinese Mainland in 2003; Unspecified style, 20 minutes per session, twice per day, 5 times per week, for 8 weeks; 20 minutes per session, twice per day, 5 times per week, for 6 weeks; 40 minutes per session, twice per day, 5 times per week, for 10 weeks; 30 minutes per time, 4 to 5 times per week, for 12 weeks. | Symptom; Physical performance: motor, balance；QOL |
| Yu TT 2014 | SR+meta | Chinese Mainland | Type 2 diabetes | The style of State General Administration of Sport of Chinese Mainland in 2003; Unspecified style; 20 minutes per time, 5 times per week, for 8 weeks; 45 minutes per session, twice per day, 5 times per week, for 24 weeks; 60 minutes per time, 5 times per week, for 16 weeks; 60 minutes per time, 5 times per week, for 24 weeks; 30 to 60 minutes per time, 5 times per week, for 8 weeks; 60 minutes per time, 4 times per week, for 24 weeks. | Laboratory tests: FPG, HbA1c, blood lipid; Laboratory tests: FPG, HbA1c, blood lipid |
| Pan PQ 2018 | SR+meta | Chinese Mainland | Type 2 diabetes | The style of State General Administration of Sport of Chinese Mainland in 2003; Unspecified style; supervised by qualified instructor, 45 minutes per time, 5 times per week, for 8 week; 30 minutes per session, twice per day, 5 times per week, for 12 weeks; 60 minutes per time, 5 times per week, for 16 weeks; 60 minutes per time, 3 times per week, for 16 weeks; 45 minutes per time, 3 to 5 times per week, for 12 weeks; 20 minutes per session, twice per day, 5 times per week, for 12 weeks; 20 to 30 minutes per time, 5 times per week, for 12 weeks; 30 minutes per time, 5 times per week, for 24 weeks. | Laboratory tests: FPG, HbA1c; QOL; Psychological: anxiety, depression, SCL-90 |
| Li H 2017 | SR+meta | Chinese Mainland | COPD | The style of State General Administration of Sport of Chinese Mainland in 2003; Unspecified style; 30 minutes per time, 4 times per week, for 24 weeks; 30 minutes per time, 5 times per week, for 12 weeks; 60 minutes per time, 5 times per week, for 48 weeks; 40 minutes per time, 5 times per week, for 4 weeks; 30 minutes per session, twice per day, 5 times per week, for 24 weeks. | Physical performance: pulmonary function |
| Han Y 2017 | SR+meta | Chinese Mainland | COPD | The style of State General Administration of Sport of Chinese Mainland in 2003; Unspecified style, 30 minutes per time, 5 times per week, for 12 weeks; 30 minutes per session, twice per day, 5 times per week, for 24 weeks; 30 minutes per time, 4 times per week, for 36 weeks; 30 minutes per time, 4 times per week, for 24 weeks. | Physical performance: pulmonary function |
| Chen YH 2018 | SR+meta | Chinese Mainland | COPD | The style of State General Administration of Sport of Chinese Mainland in 2003; Unspecified style; 30 minutes per time, 4 times per week, for 24 weeks; 30 minutes per time, 5 times per week, for 12 weeks; 60 minutes per time, 5 times per week, for 48 weeks; 40 minutes per time, 5 times per week, for 4 weeks; 30 minutes per session, twice per day, 5 times per week, for 24 weeks. | Physical performance: pulmonary function; QOL |
| Wang JY 2016 | SR+meta | Chinese Mainland | Health promotion | The style of State General Administration of Sport of Chinese Mainland in 2003; Unspecified style; 40 to 50 minutes per time, 5 times per week, for 8 weeks; 40 to 50 minutes per time, 5 times per week, for 12 weeks; 30 minutes per session, twice per day, 5 times per week, for 12 weeks; 45 to 60 minutes per time, 5 to 7 times per week, for 48 weeks; 60 minutes per time, 6 times per week, for 12 weeks; 45 minutes per session, twice per day, 5 times per week, for 24 weeks. | Laboratory tests: blood lipid; Physical performance: blood pressure, pulmonary function |
| Qin J 2018 | SR+meta | Chinese Mainland | Hypertension | The style of State General Administration of Sport of Chinese Mainland in 2003; Homemade style; Unspecified style; Other style; 15 to 20 minutes per session, twice per day, 5 times per week, for 3 weeks; 45 minutes per session, twice per day, 5 times per week, for 24 weeks; 30 minutes per session, twice per day, 5 times per week, for 12 weeks; 20 to 40 minutes per time, 3 to 5 times per week, for 12 weeks; 60 minutes per session, twice per day, 3 to 4 times per week, for 12 weeks; 20 minutes per session, twice per day, 5 times per week, for 24 weeks; 30 to 40 minutes per time, 5 times per week, for 48 weeks; 60 minutes per time, 5 times per week, for 12 weeks; 20 minutes per session, twice per day, 5 times per week, for 24 weeks. | Laboratory tests: blood lipid; Physical performance: blood pressure, BMI |
| Qiu DR 2019 | SR+meta | Chinese Mainland | Depression | The style of State General Administration of Sport of Chinese Mainland in 2003; Homemade style; Unspecified style; 30 minutes per time, 4 times per week, for 24 weeks; 40 minutes per time, 3 to 5 times per week, for 12 weeks; 60 minutes per time, 5 times per week, for 24 weeks; 30 minutes per time, 5 times per week, for 4 weeks; 20 minutes per session, twice per day, 5 times per week, for 8 weeks; 30 minutes per session, twice per day, 5 times per week, for 6 weeks. | Psychological: anxiety, depression |
| Lin JH 2017 | SR+meta | Chinese Mainland | Hypertension | The style of State General Administration of Sport of Chinese Mainland in 2003; Homemade style; Unspecified style; Other style; 30 minutes per time, 5 times per week, for 12 weeks; 30 minutes per session, twice per day, 5 times per week, for 12 weeks; 40 minutes per time, 5 times per week, for 24 weeks; 45 minutes per session, twice per day, 5 times per week, for 24 weeks; 60 minutes per session, twice per day, 3 to 4 times per week, for 12 weeks; 20 minutes per session, twice per day, 5 times per week, for 24 weeks. | Physical performance: blood pressure |
| Chen ZS 2018 | SR+meta | Chinese Mainland | Hypertension | The style of State General Administration of Sport of Chinese Mainland in 2003; Homemade style; Unspecified style; Other style; 15 to 20 minutes per session, twice per day, 5 times per week, for 3 weeks; 45 minutes per session, twice per day, 5 times per week, for 24 weeks; 30 minutes per session, twice per day, 5 times per week, for 12 weeks; 30 minutes per time, 5 times per week, for 12 weeks; 60 minutes per session, twice per day, 3 to 4 times per week, for 12 weeks; 20 minutes per session, twice per day, 5 times per week, for 24 weeks; 30 to 40 minutes per time, 5 times per week, for 48 weeks; 60 minutes per time, 5 times per week, for 12 weeks; 20 minutes per session, twice per day, 5 times per week, for 24 weeks. | Laboratory tests: blood lipid; Physical performance: blood pressure, BMI |
| Yang JP 2015 | SR+meta | Chinese Mainland | Type 2 diabetes | The style of State General Administration of Sport of Chinese Mainland in 2003; Unspecified style, 30 minutes per time, 5 times per week, for 12 weeks; 45 minutes per session, twice per day, 5 times per week, for 24 weeks; 60 minutes per time, 5 times per week, for 16 weeks. | Laboratory tests: blood glucose, blood lipid |
| Zhang BZ 2019 | SR+meta | Chinese Mainland | Hyperlipidemia | The style of State General Administration of Sport of Chinese Mainland in 2003; Deng Tietao style; Unspecified style; 30 minutes per session, twice per day, 5 times per week, for 12 weeks; 60 minutes per time, 5 times per week, for 24 weeks; 60 minutes per time, 5 times per week, for 12 weeks; 20 minutes per session, twice per day, 5 times per week, for 24 weeks. | Laboratory tests: blood lipid; adverse reactions |

***Notes:*** AIDS, acquired immunodeficiency syndrome; BMD, Bone Mass Density; CCS, non-randomized controlled clinical studies (quasi-randomized clinical trial or observational studies such as cohort or case-control study); CS, case series; COPD, chronic obstructive pulmonary disease; CR, case report; HAMD, Hamilton Depression Scale; JOA Score, Japanese Orthopaedic Association scores; MoCA, Montreal Cognitive Assessment; NDI, Neck Disabilitv Index; PCI, percutaneous coronary intervention; PSQI, Pittsburgh Sleep Quality Index; QOL, quality of life; RCT, randomized clinical trials; SCL-90, Symptom Checklist 90; SF-36: the Medical Outcomes Study 36-Item Short-Form Health Survey; SR, systematic review; USA, United States of America; WHOQOL-BREF, World Health Organization Quality of Life Brief Scale; WOMAC, The Western Ontario and McMaster Universities Arthritis Index.
